# Supplementary material for: Stress-induced tyrosine phosphorylation of RtcB modulates IRE1 activity and signaling outputs
Source: Life Sci Alliance. 2022 Feb 22;5(5):e202201379. doi: 10.26508/lsa.202201379 (PMC8899846; doi:10.26508/lsa.202201379)

J.Repeat n.1

Supplementary Figure 1.

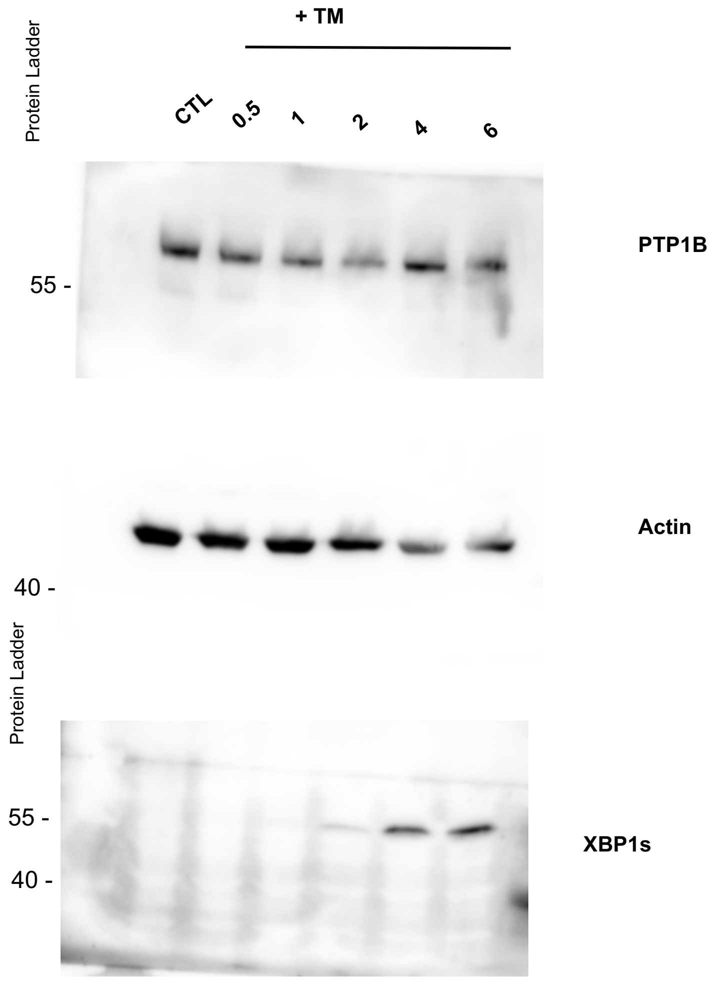

Supplementary Figure 1.

J.Repeat n.2

In Fig.S1J

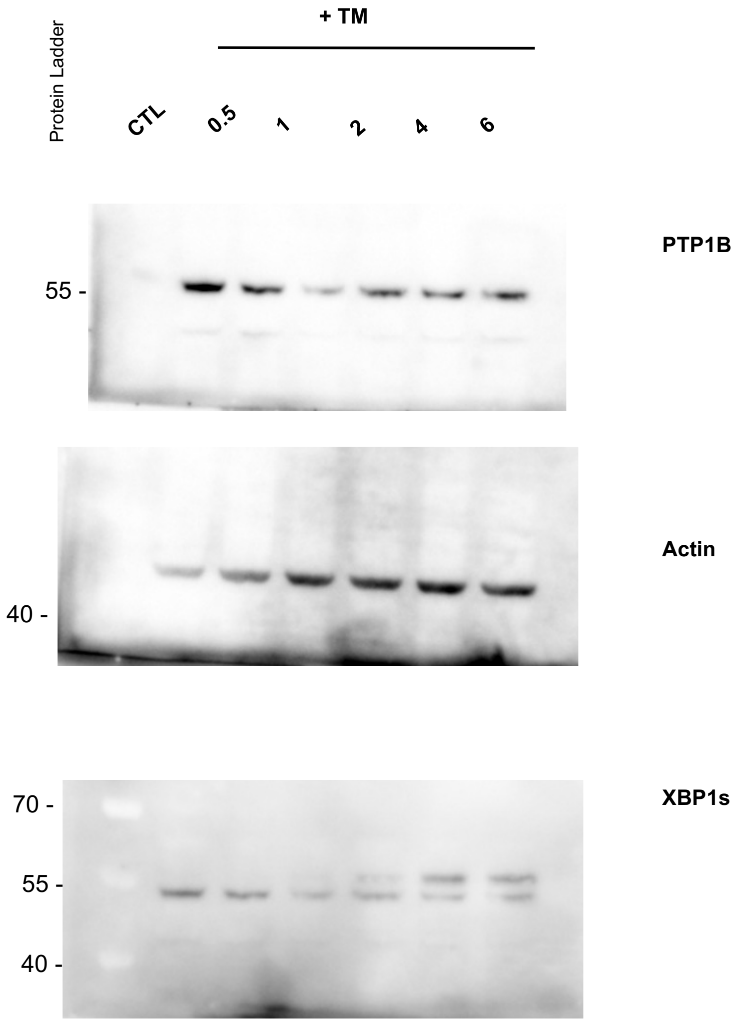

J.Repeat n.3

Supplementary Figure 1.

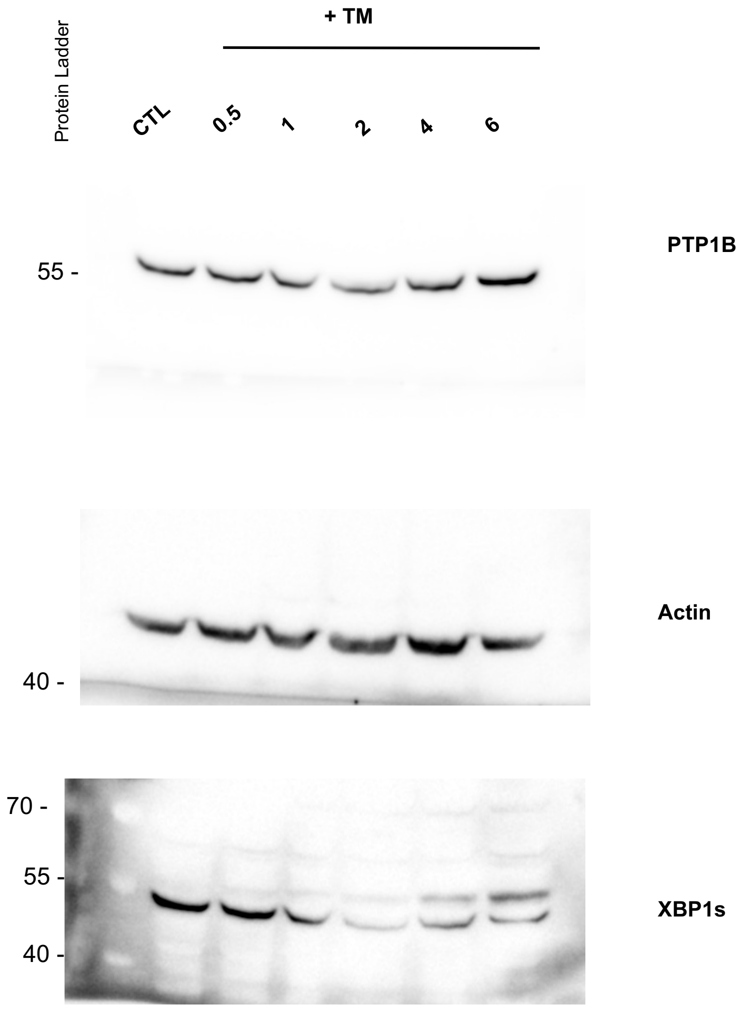

Supplement: Supplementary file 1 [file LSA-2022-01379_SdataFS1.zip › Source data FigS1/Source blots figS1.pdf]
