# Supplementary material for: Stress-induced tyrosine phosphorylation of RtcB modulates IRE1 activity and signaling outputs
Source: Life Sci Alliance. 2022 Feb 22;5(5):e202201379. doi: 10.26508/lsa.202201379 (PMC8899846; doi:10.26508/lsa.202201379)
Supplement: Supplementary file 2 [file LSA-2022-01379_TableS1.pdf]

Supplementary Table 1.

Table S1: RtcB tyrosine residues found to be phosphorylated in mass spectrometry studies.

Tyrosine	Phosphorylated	Source
5	✗	-
37	✗	-
92	✗	-
184	✗	-
196	✗	-
228	✓	Bian et. al., 2016
241	✓	Bian et. al., 2016
306	✓	PhosphositePlus: Tsai et. al., 2015
316	✓	Bian et. al., 2016
349	✓	PhosphositePlus, Bian et. al., 2016
392	✗	-
410	✗	-
475	✓	Bian et. al., 2016
