# Supplementary material for: Stress-induced tyrosine phosphorylation of RtcB modulates IRE1 activity and signaling outputs
Source: Life Sci Alliance. 2022 Feb 22;5(5):e202201379. doi: 10.26508/lsa.202201379 (PMC8899846; doi:10.26508/lsa.202201379)
Supplement: Supplementary file 3 [file LSA-2022-01379_SdataFS2.zip › Source data FigS2/Source blots figS2.pdf]

B.Repeat n.1

Supplementary Figure 2.

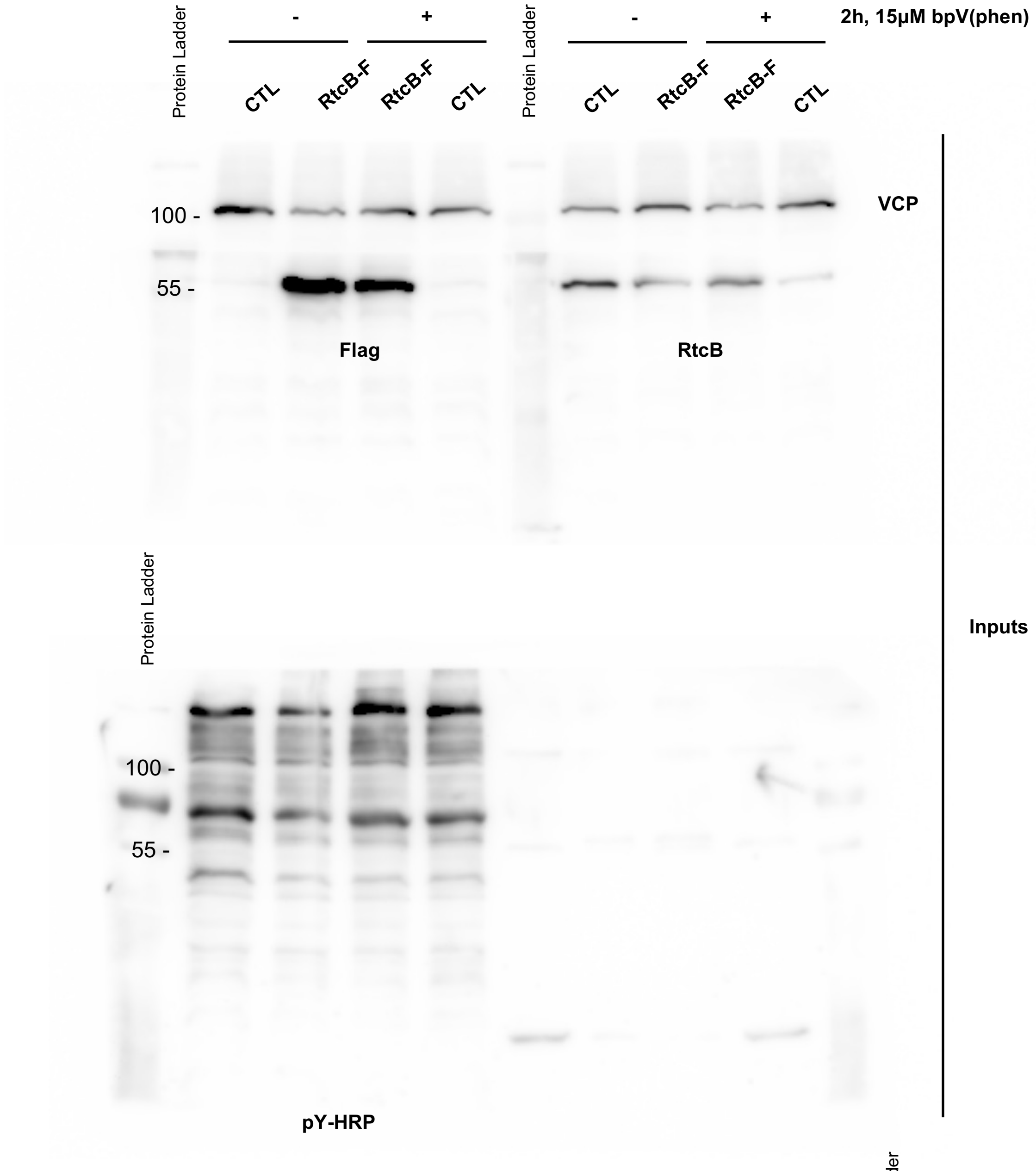

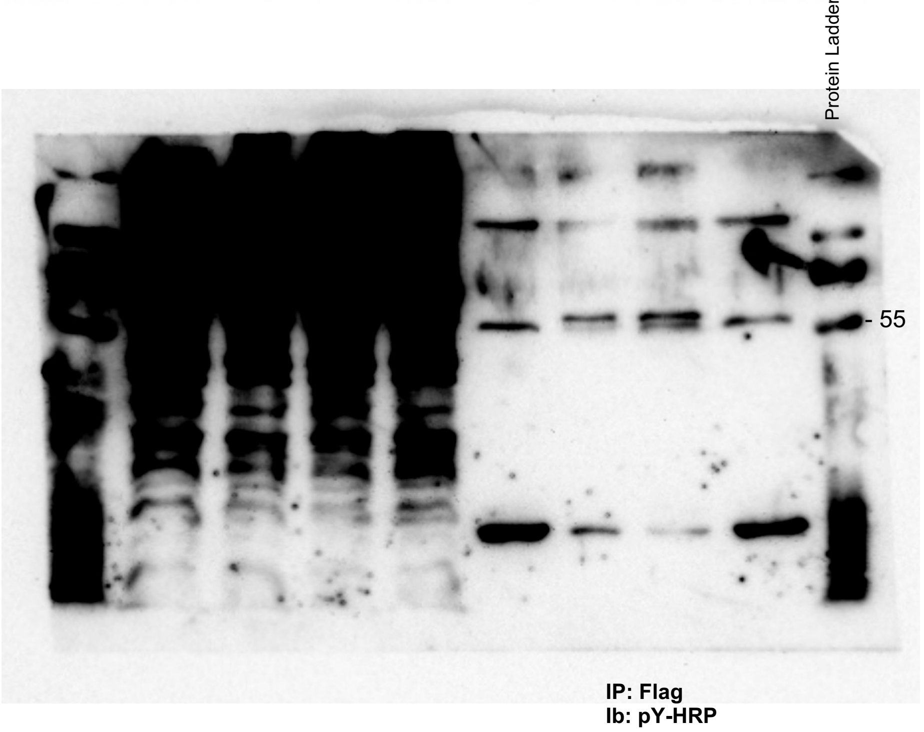

B.Repeat n.2

Supplementary Figure 2.

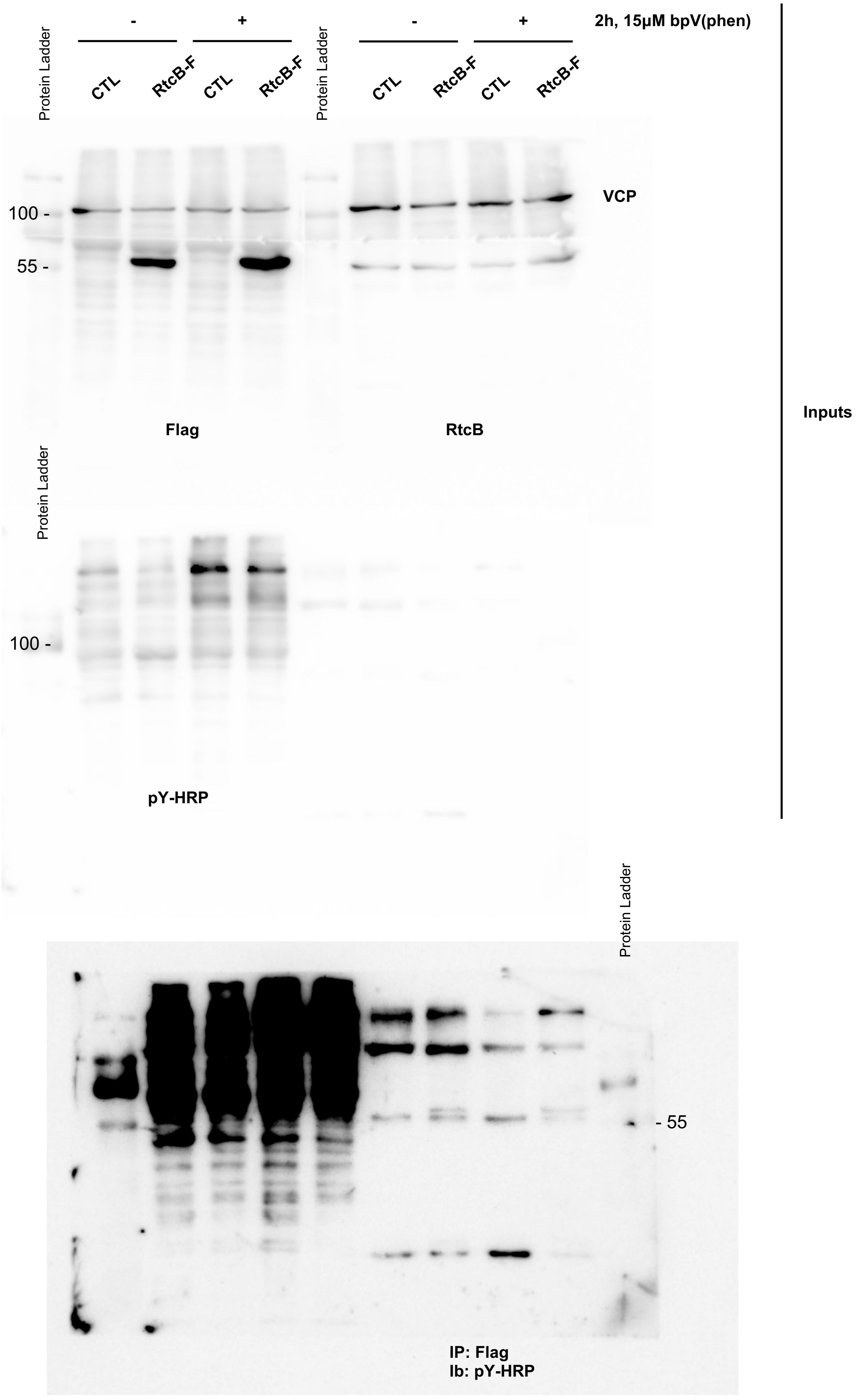

B.Repeat n.3

Supplementary Figure 2.

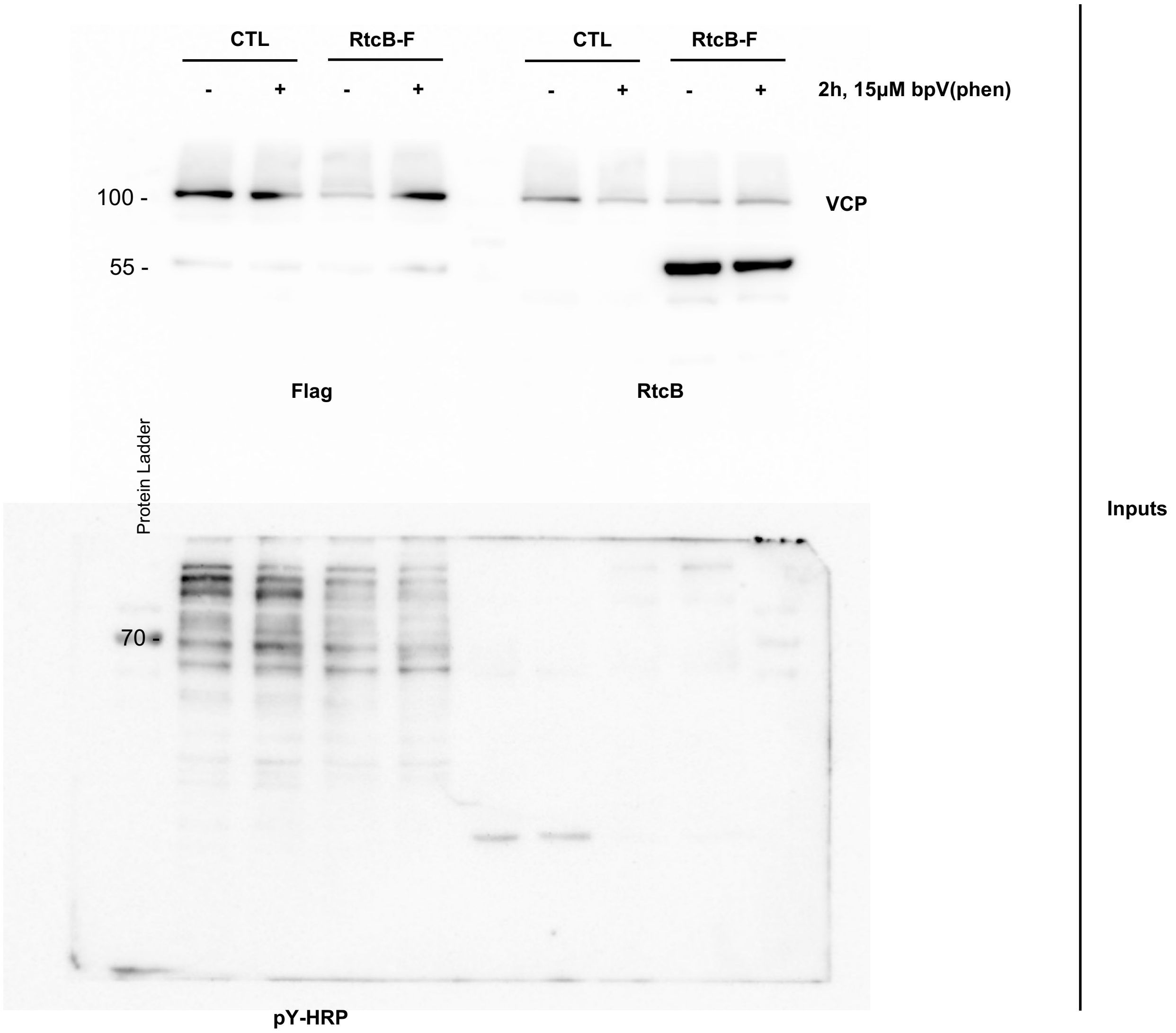

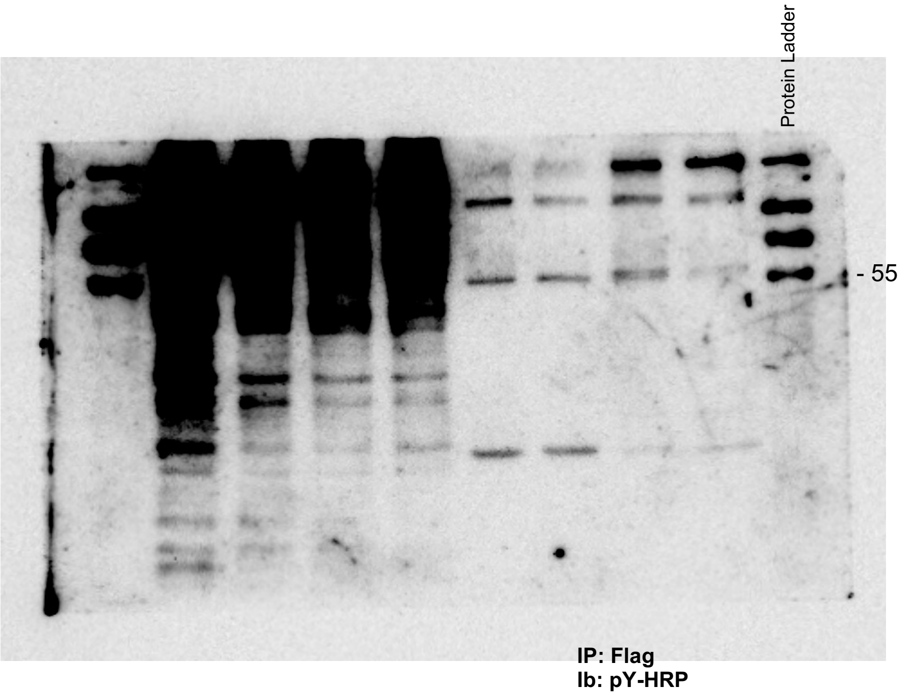

B.Repeat n.4

In Fig.S2B

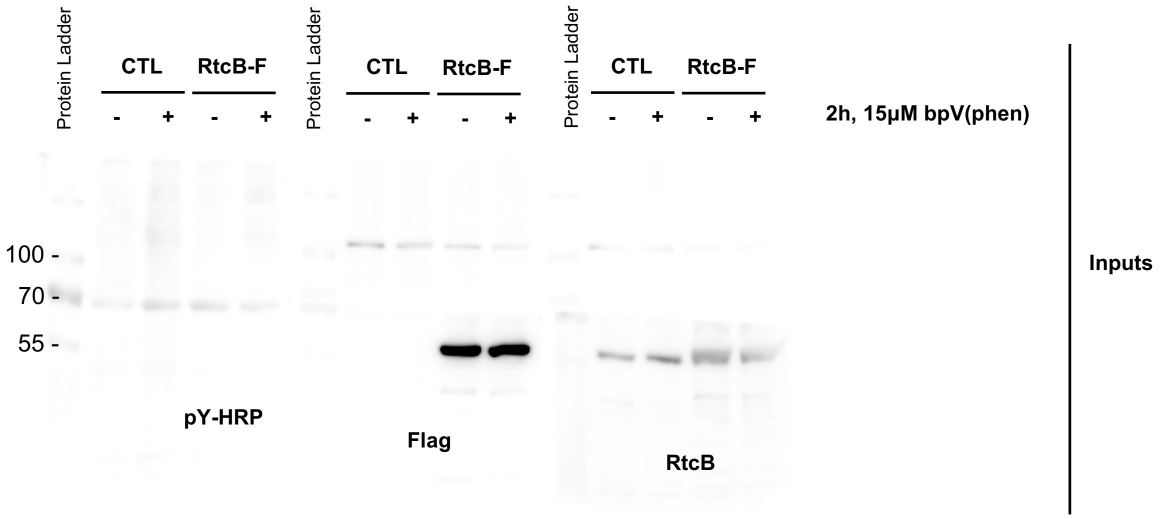

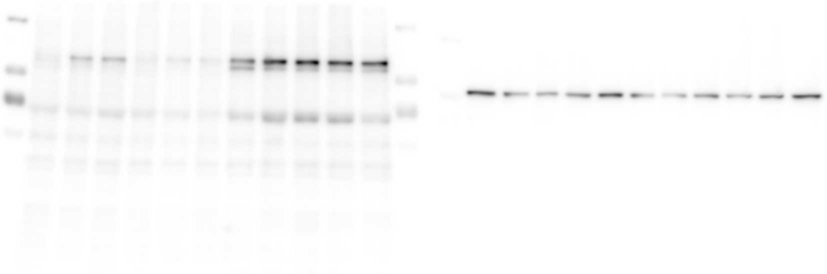

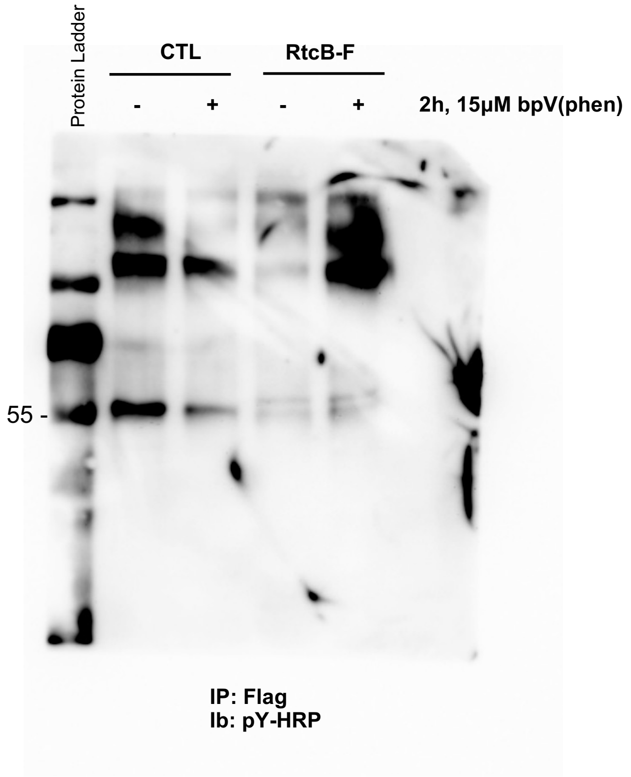

C

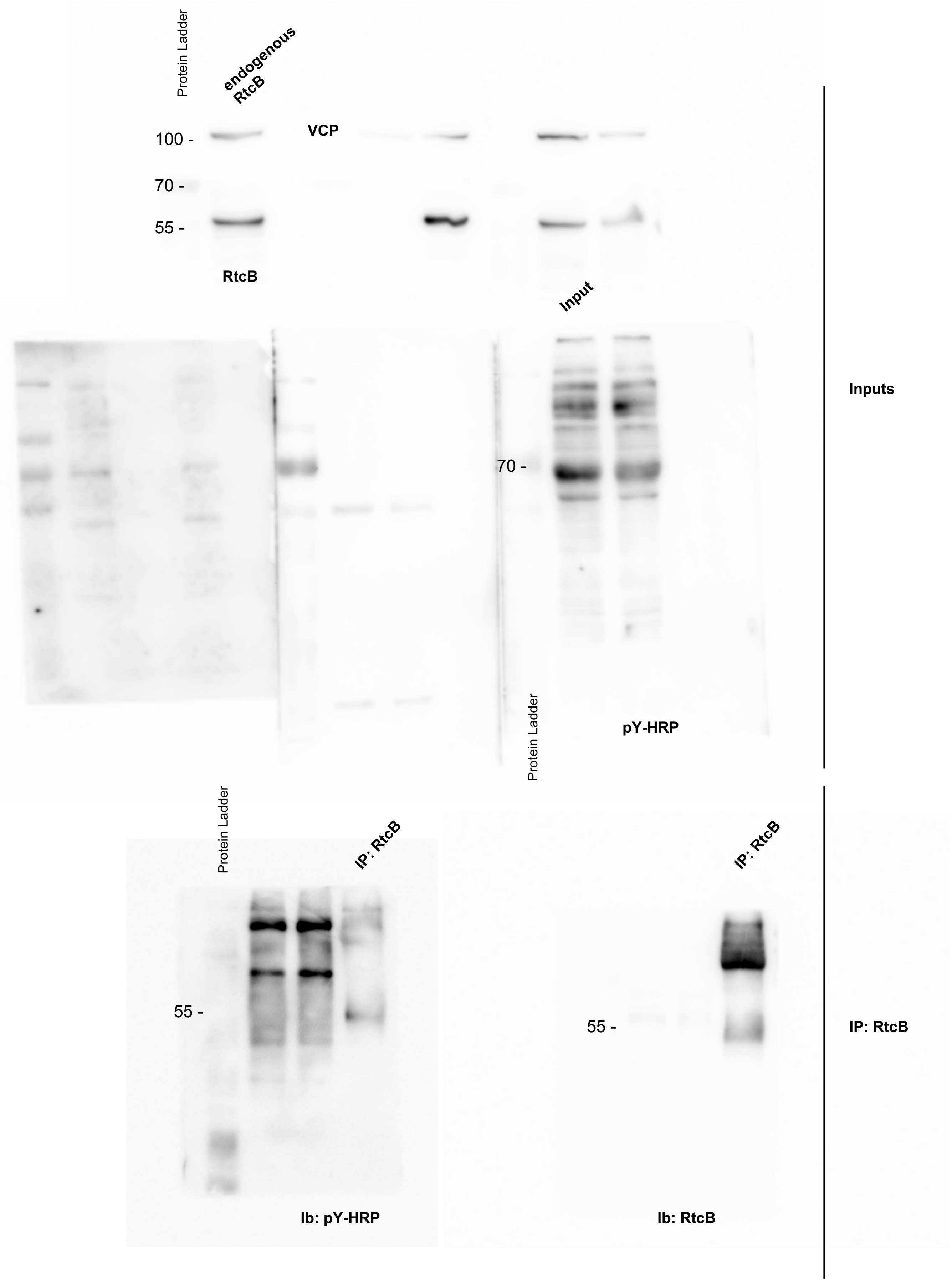
Protein Ladder
endogenous RtcB
100 -
VCP
70 -
55 -
RtcB
Input
70 -
Protein Ladder
pY-HRP
Inputs
Protein Ladder
IP: RtcB
55 -
Ib: pY-HRP
IP: RtcB
55 -
Ib: RtcB
IP: RtcB

D

In Fig.S2D

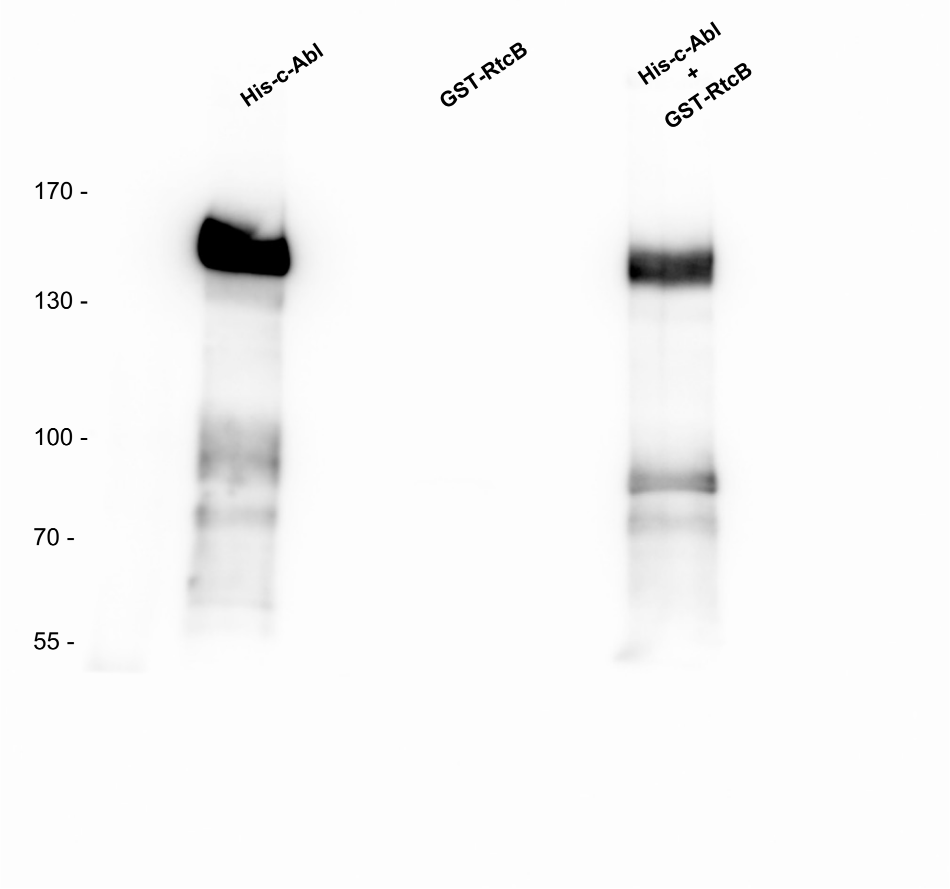

pY-HRP

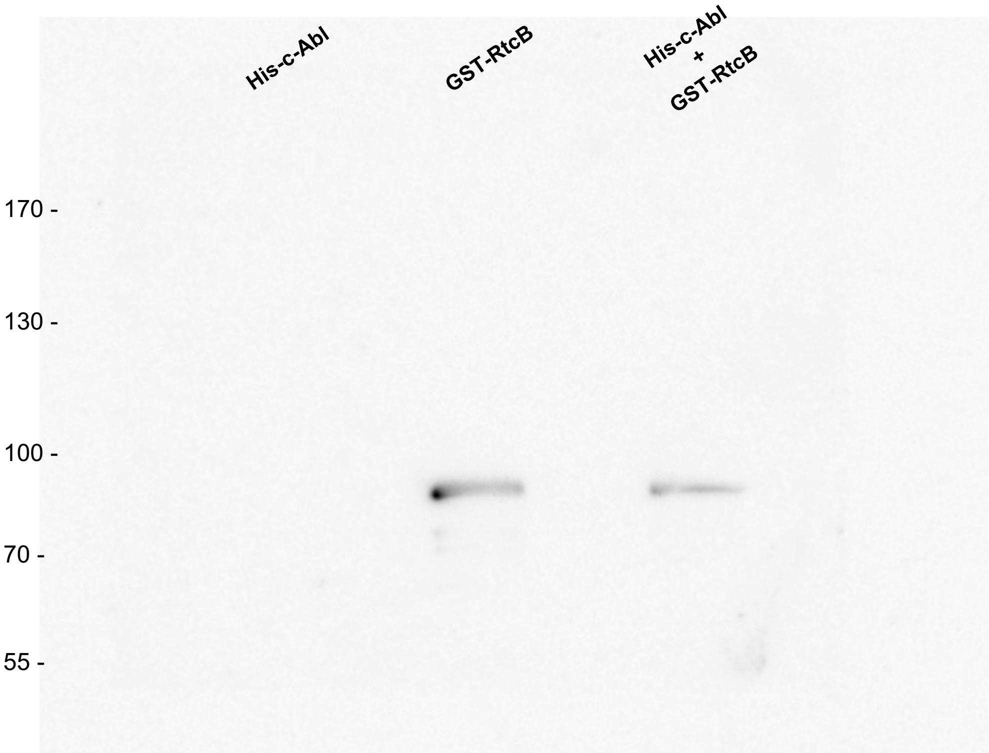

RtcB
