# Supplementary material for: Stress-induced tyrosine phosphorylation of RtcB modulates IRE1 activity and signaling outputs
Source: Life Sci Alliance. 2022 Feb 22;5(5):e202201379. doi: 10.26508/lsa.202201379 (PMC8899846; doi:10.26508/lsa.202201379)

A.Repeat n.1

Figure 2.

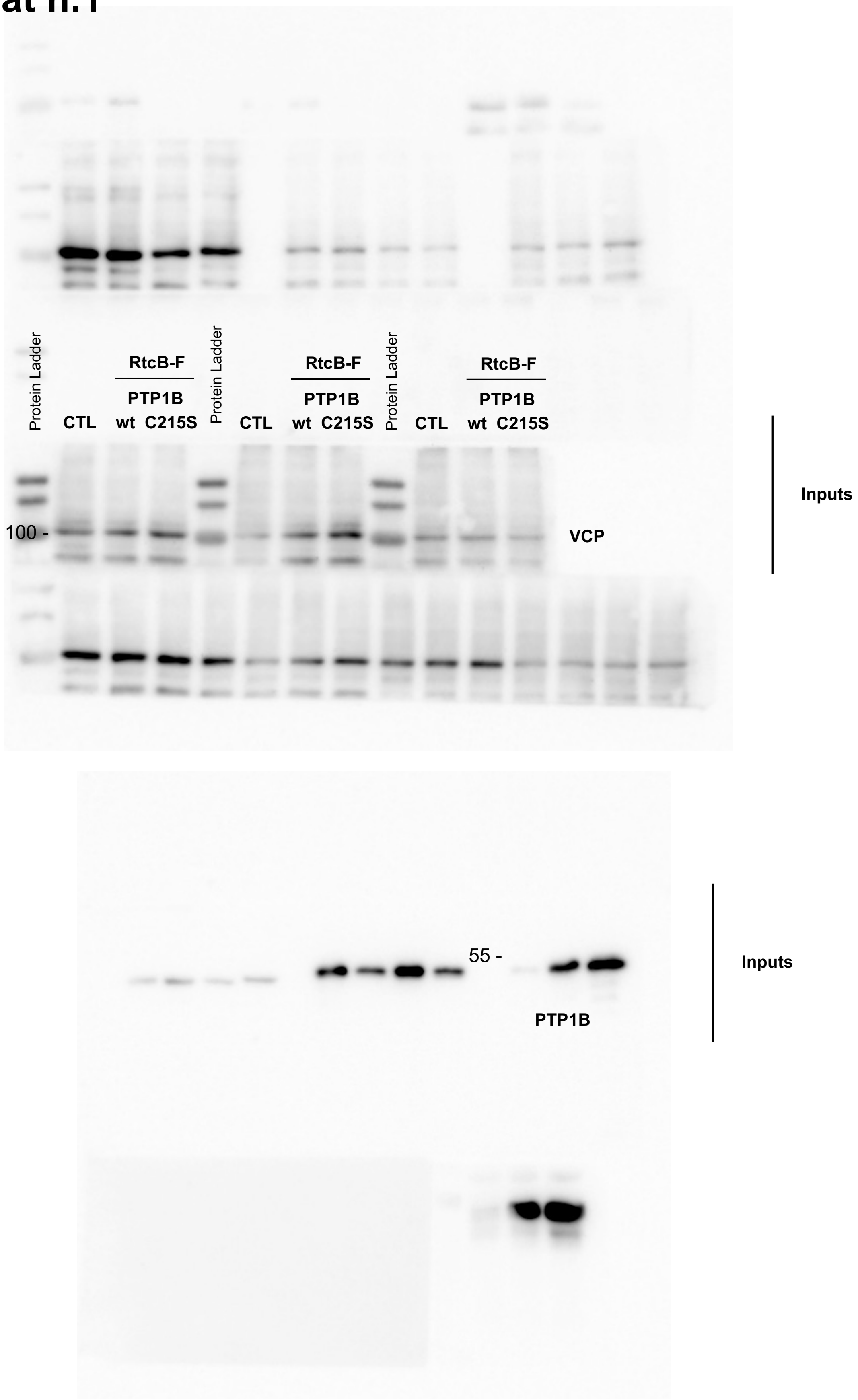

Figure 2.

A.Repeat n.1

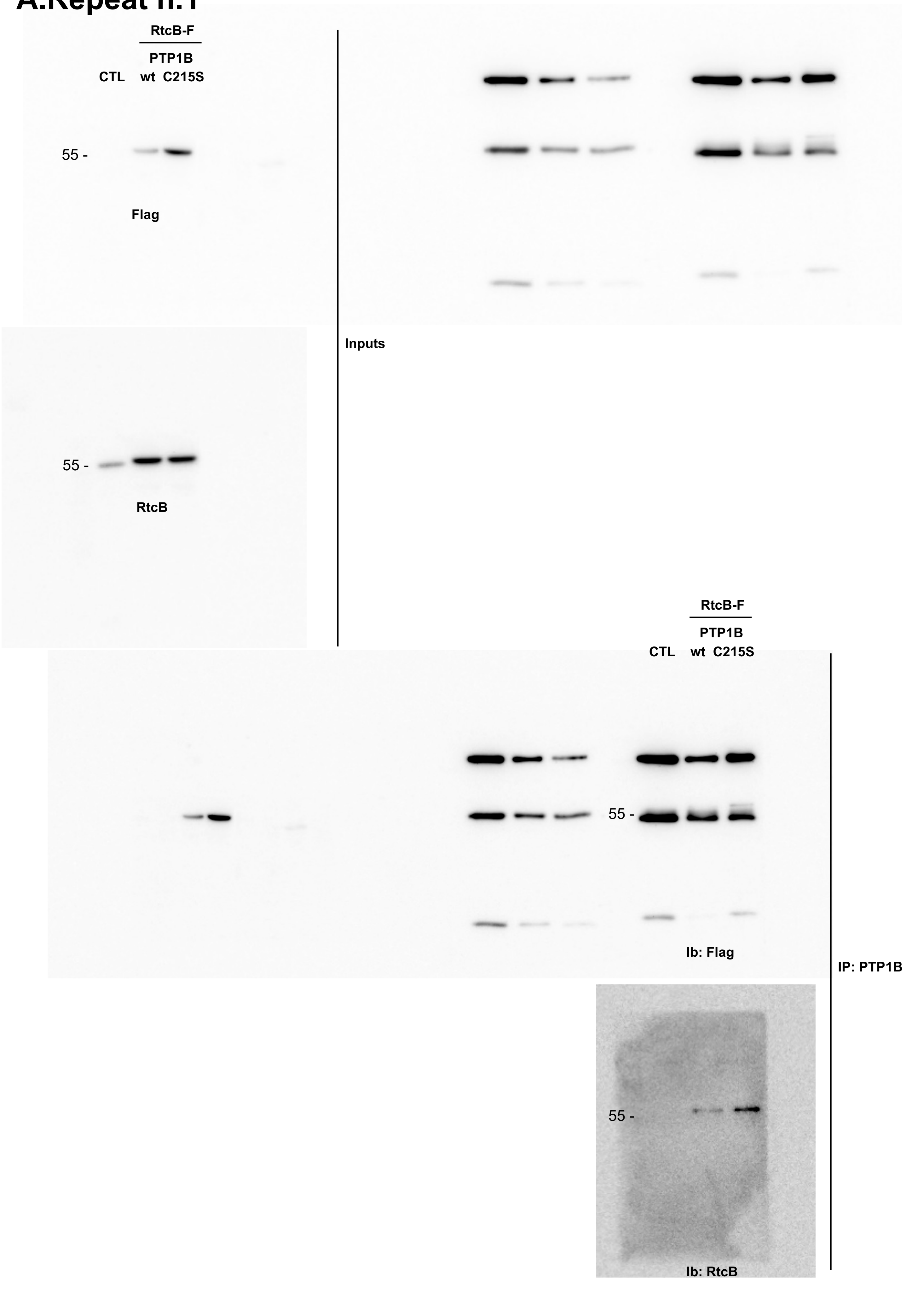

Figure 2.

A.Repeat n.2
In Fig.2A

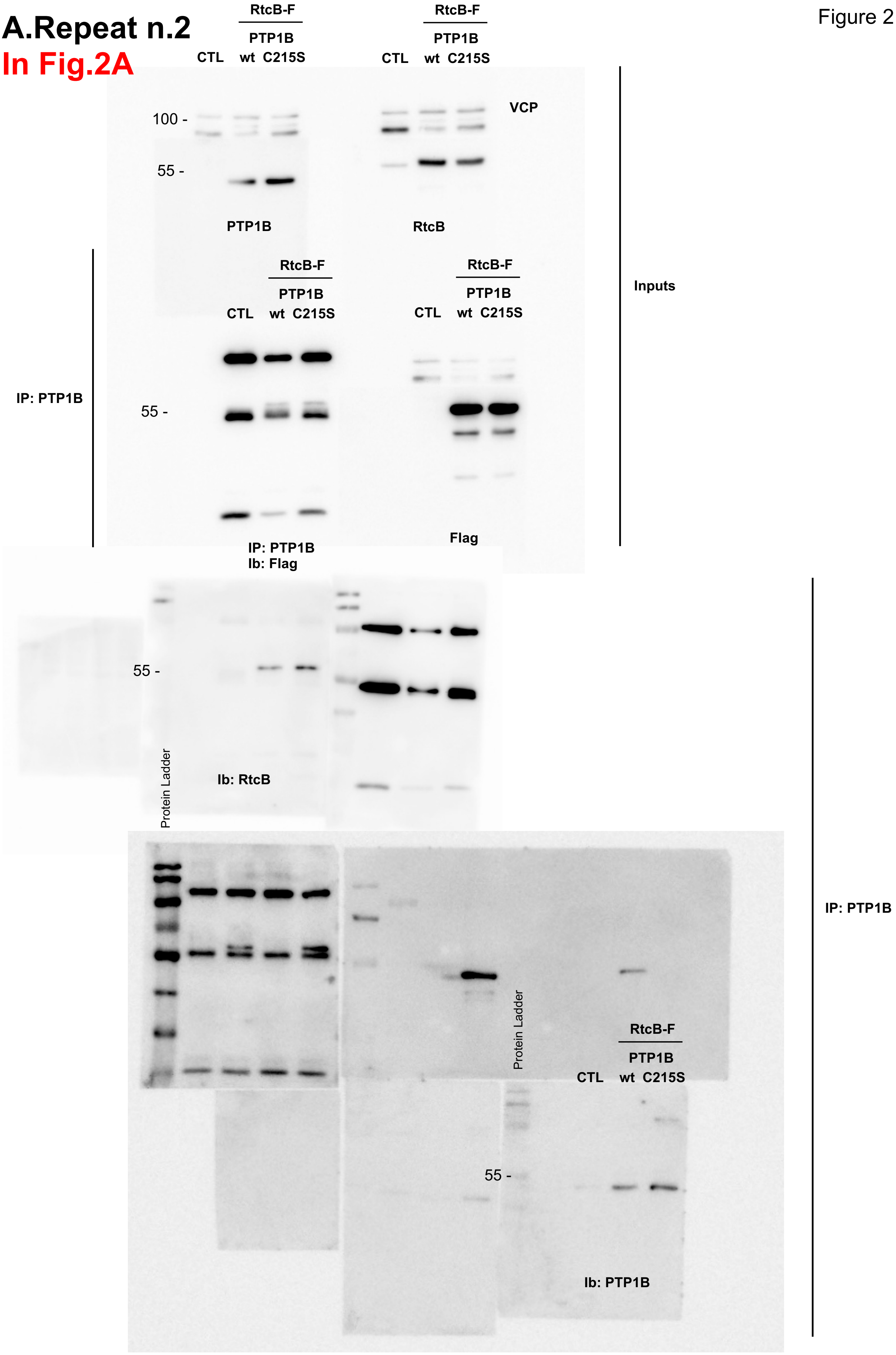

A.Repeat n.3

Figure 2.

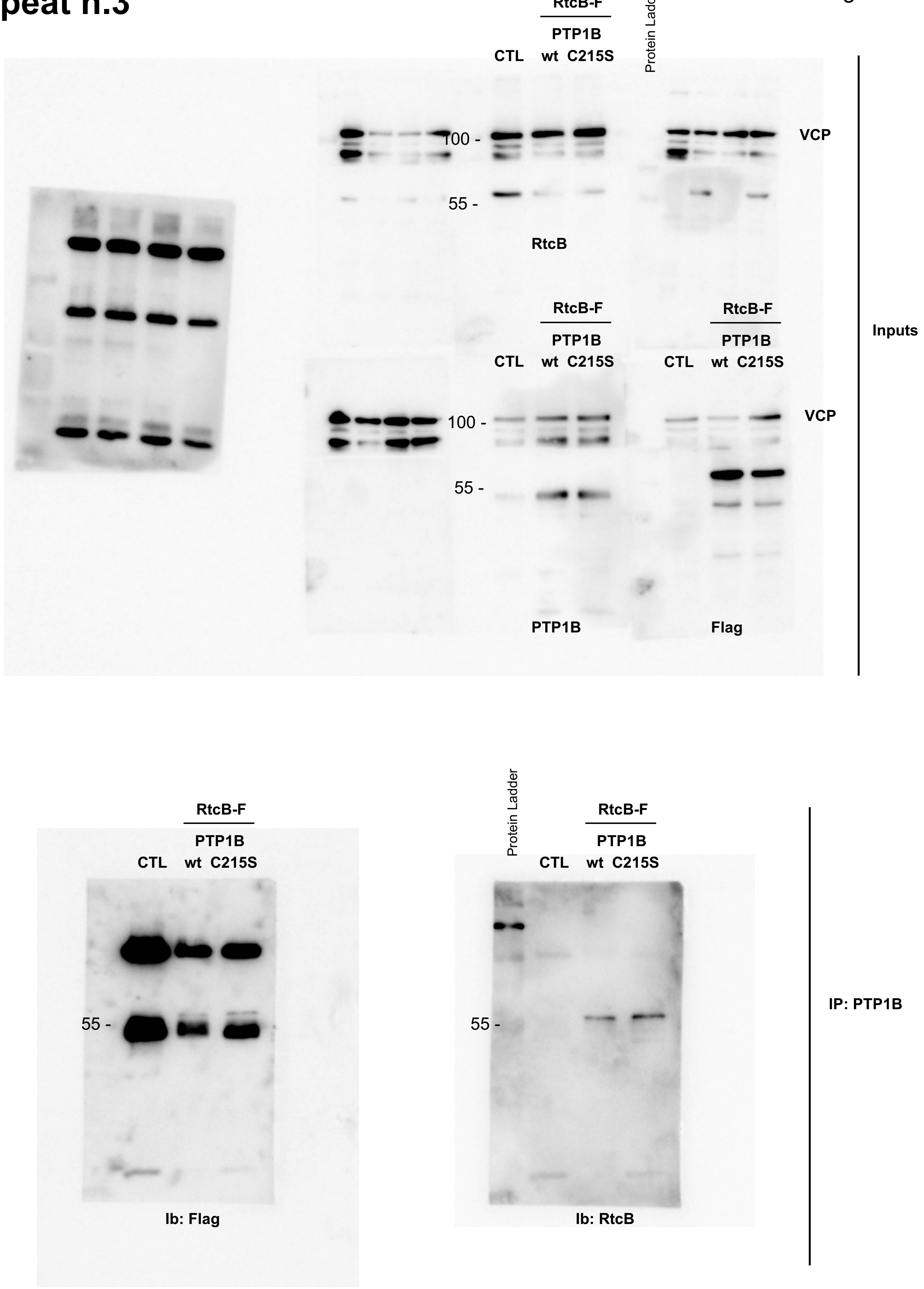

Figure 2.

B.Repeat n.1

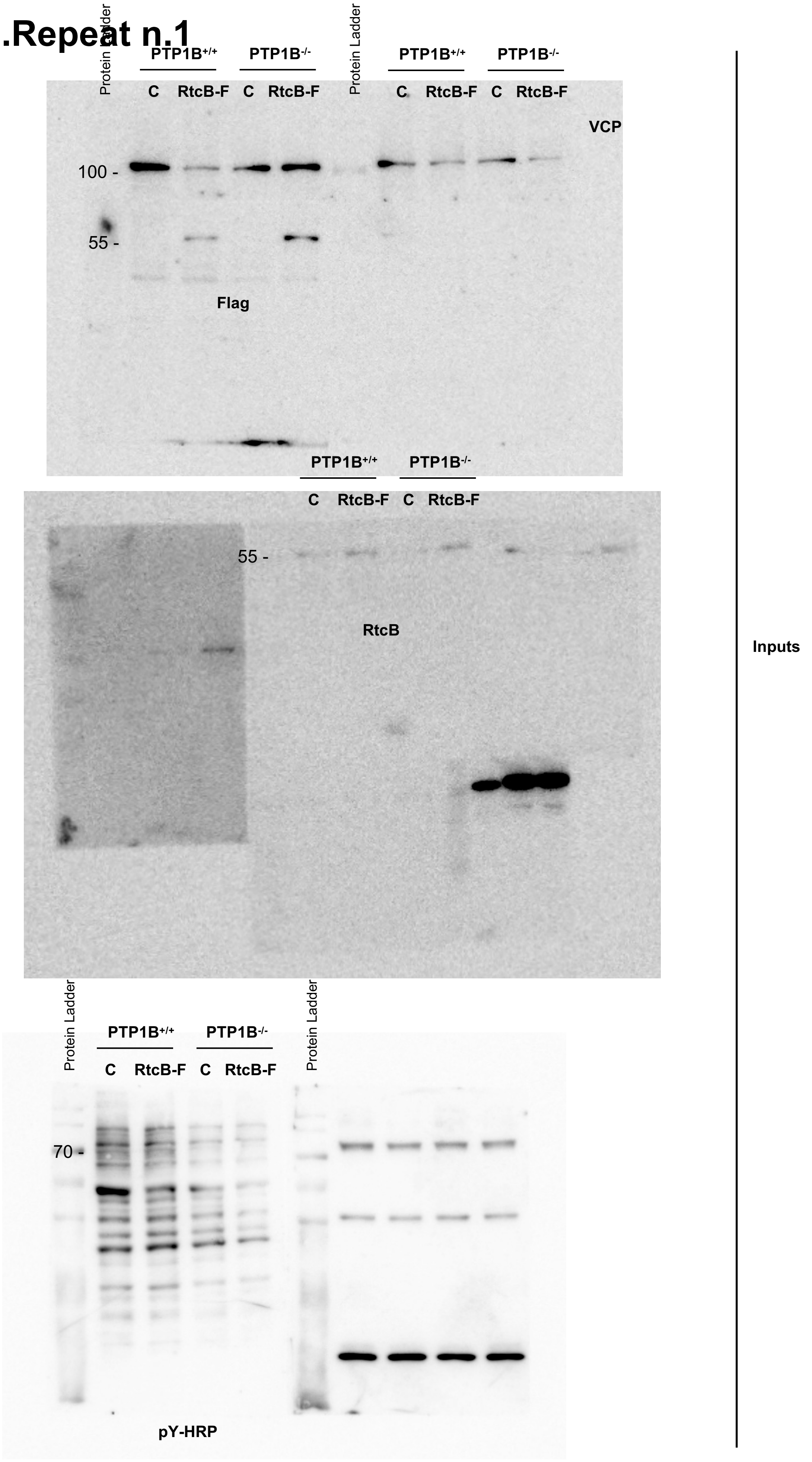

B.Repeat n.1

Figure 2.

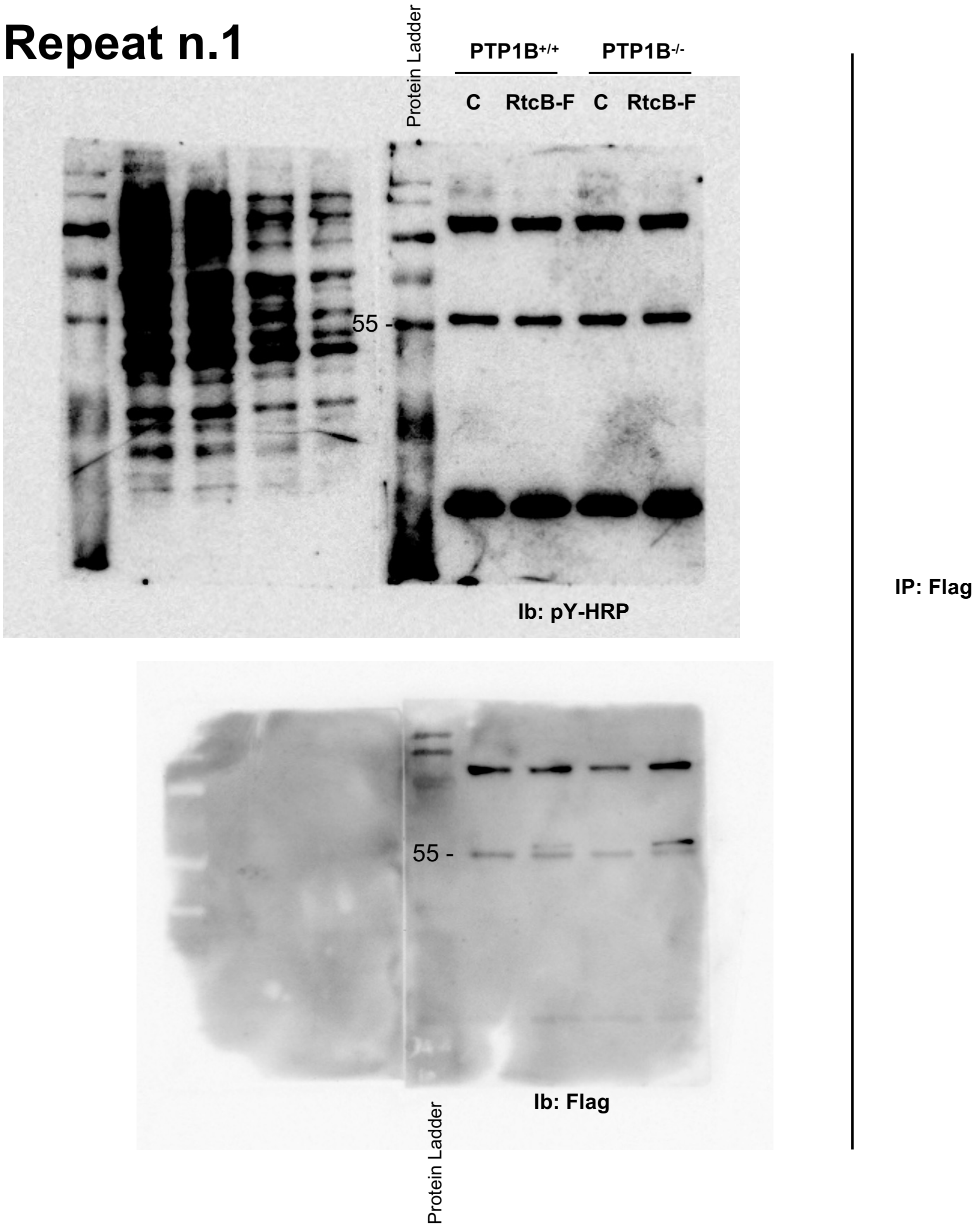

B.Repeat n.2

In Fig.2B

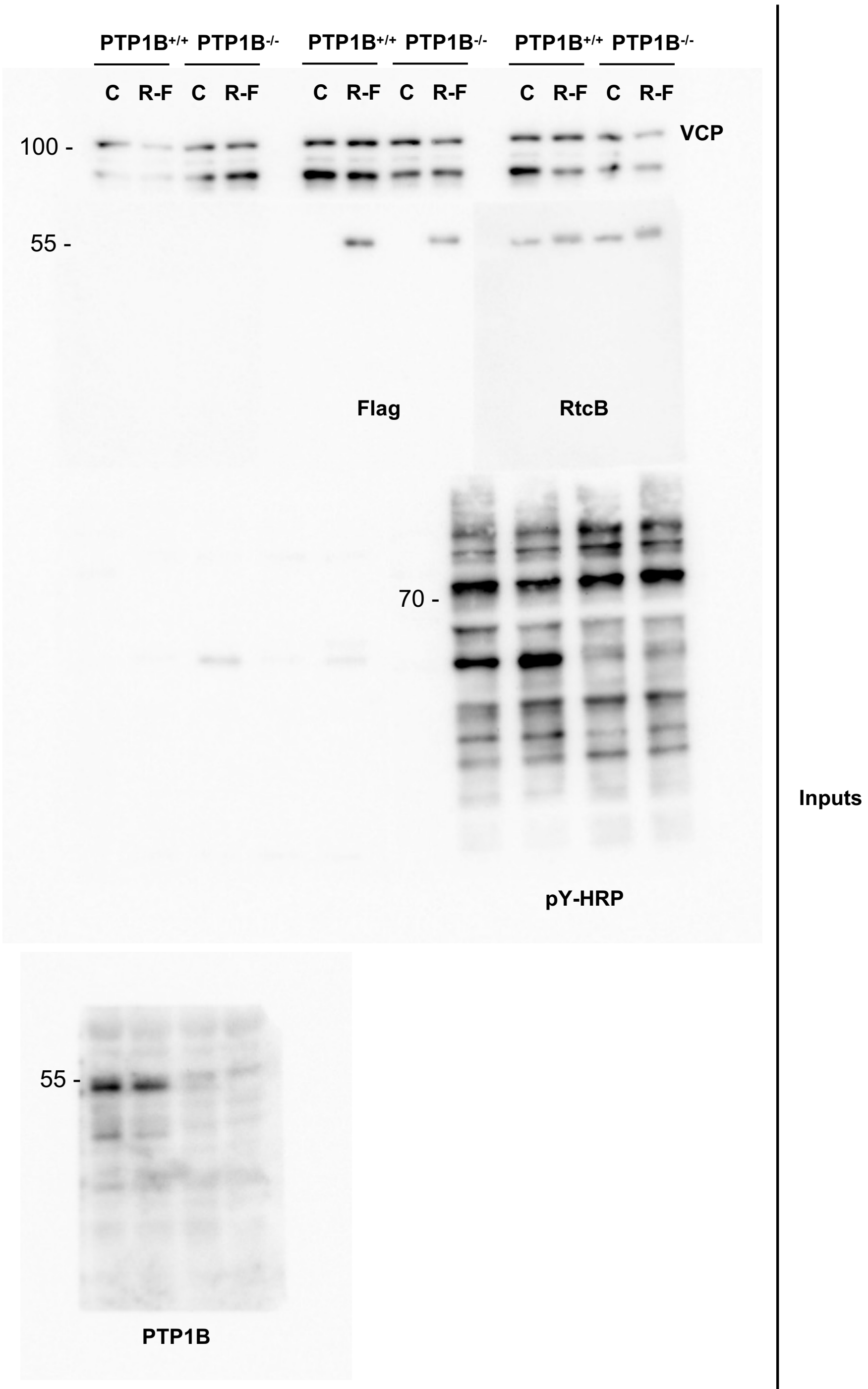

B.Repeat n.2
In Fig.2B

Figure 2.

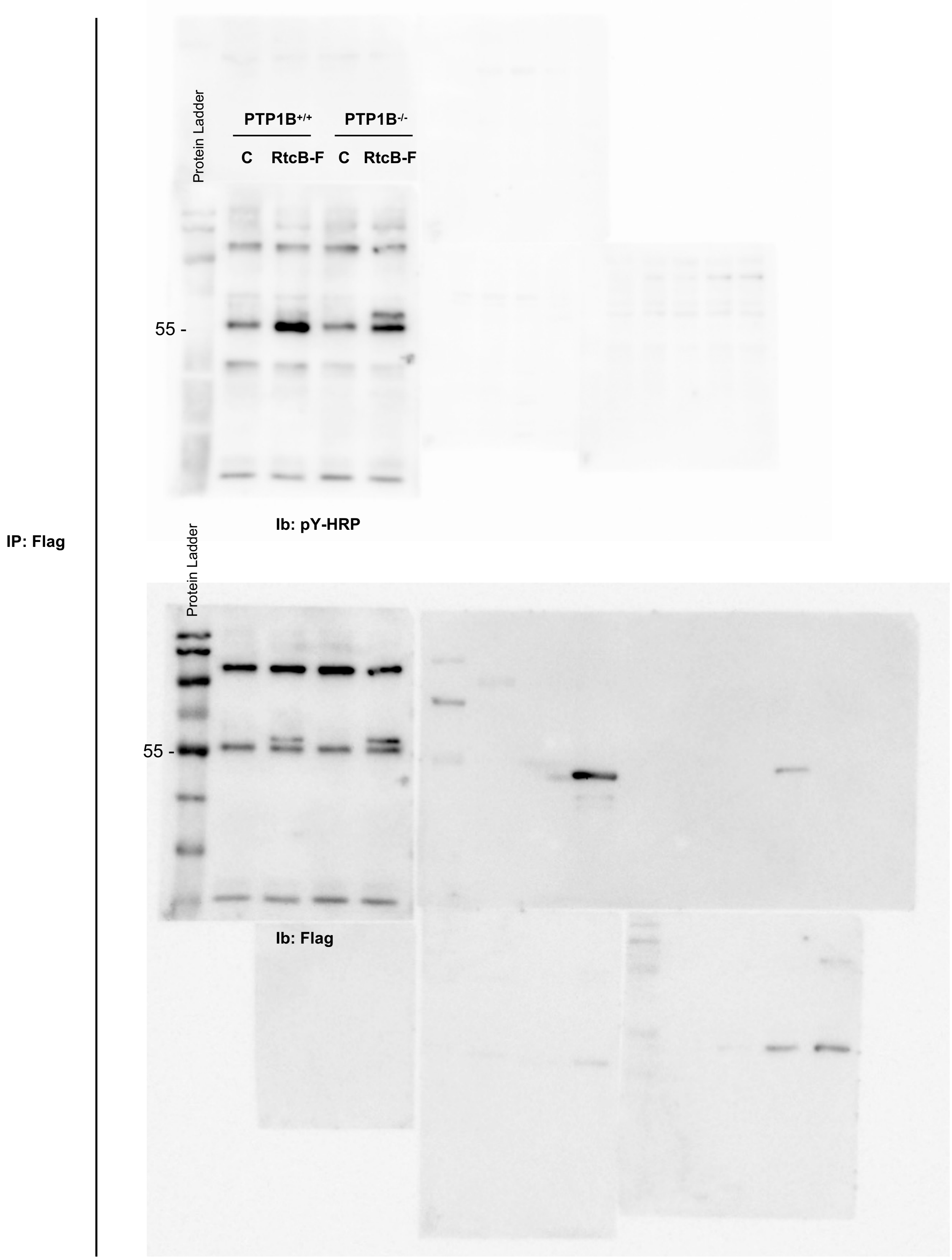

B.Repeat n.3

Figure 2.

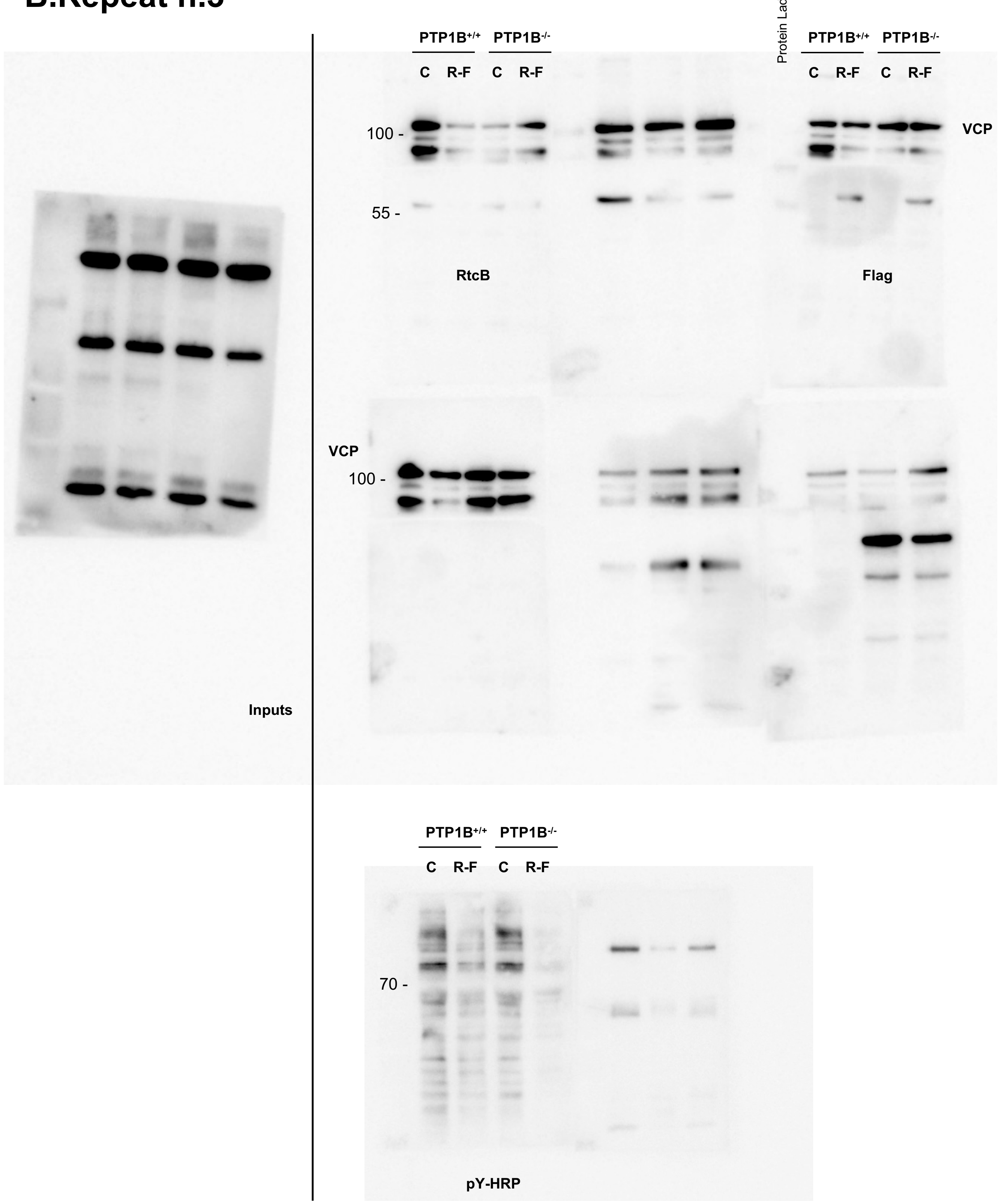

B.Repeat n.3

Figure 2.

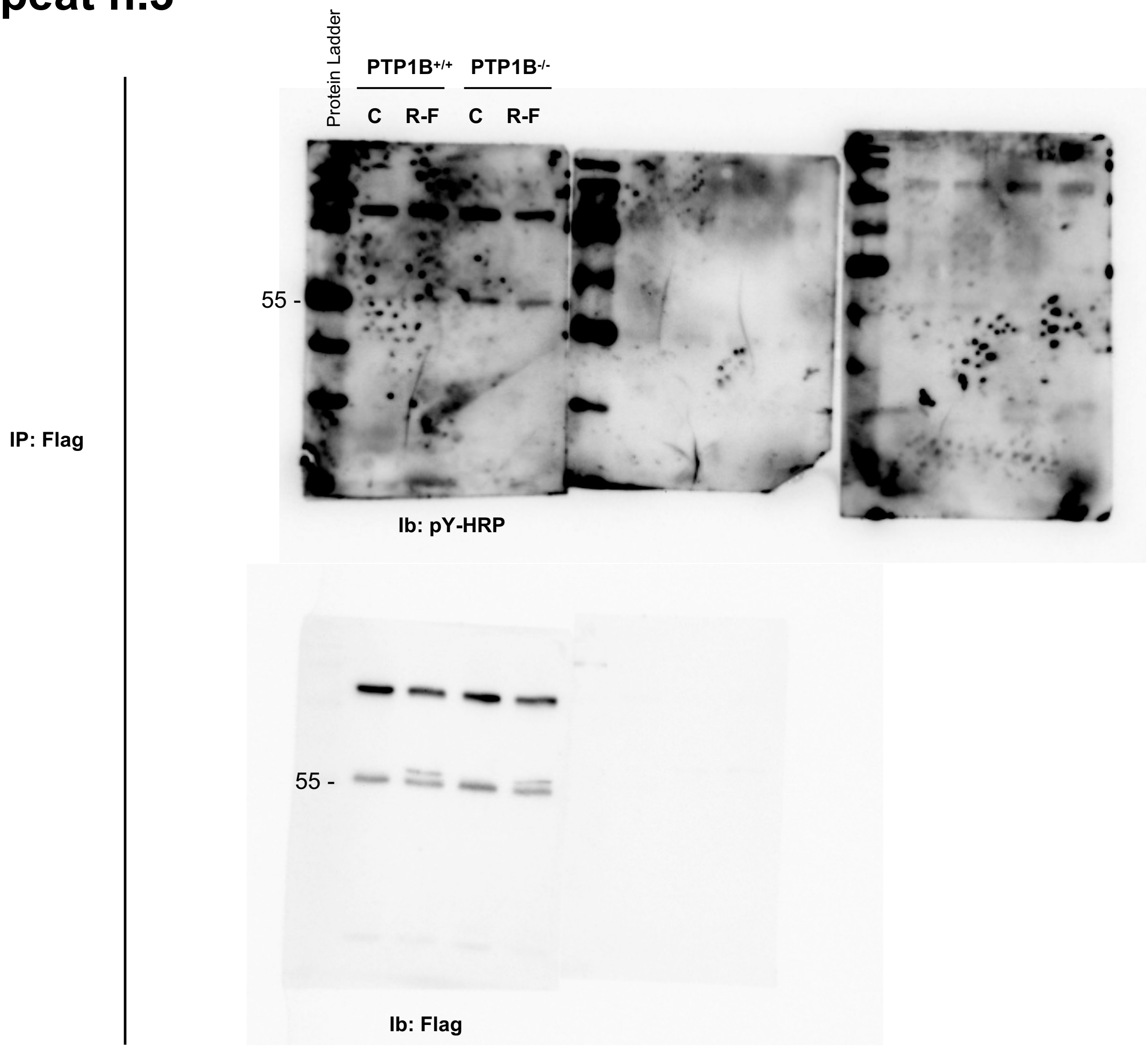

B.Repeat n.4

Figure 2.

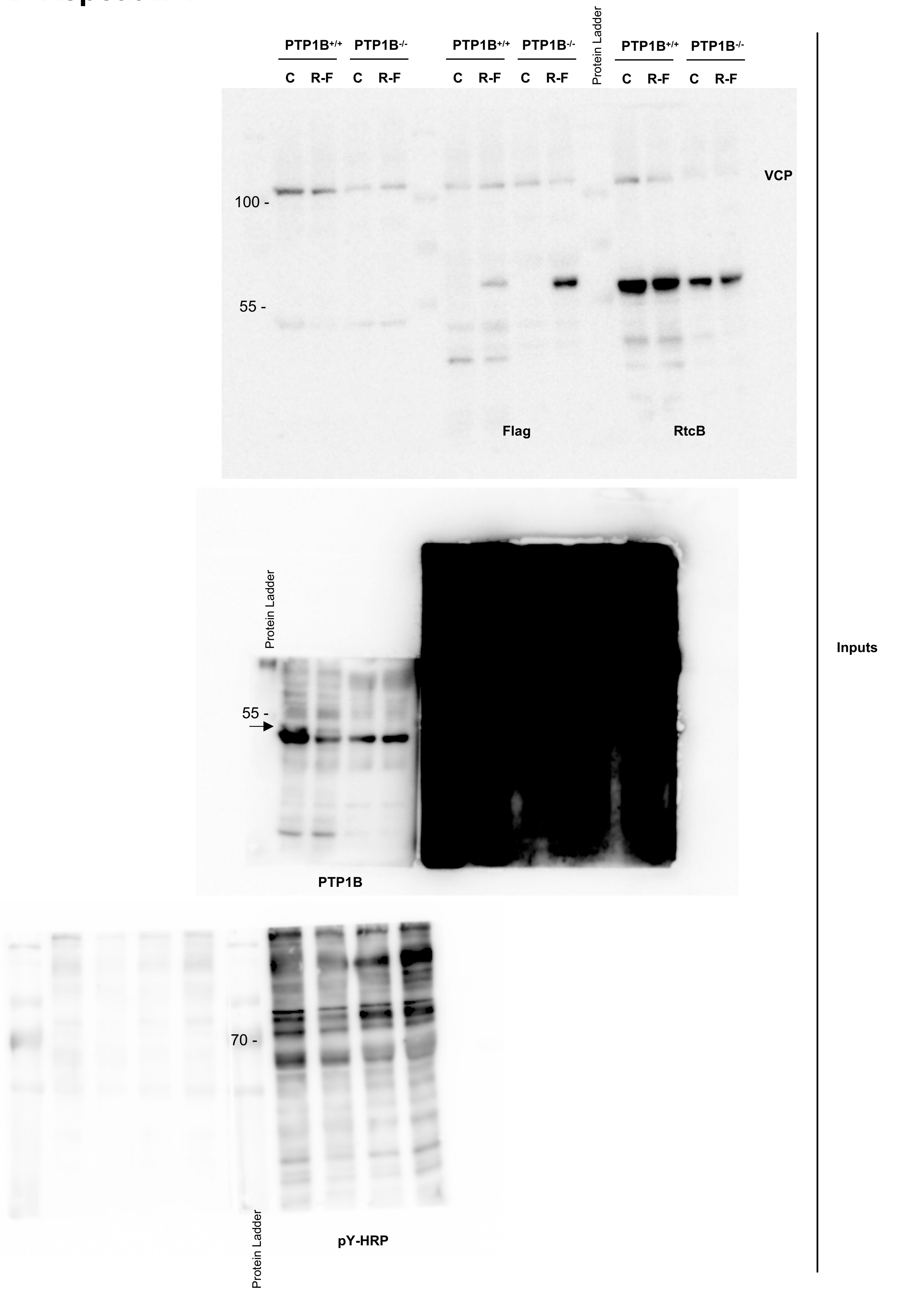

B.Repeat n.4

Figure 2.

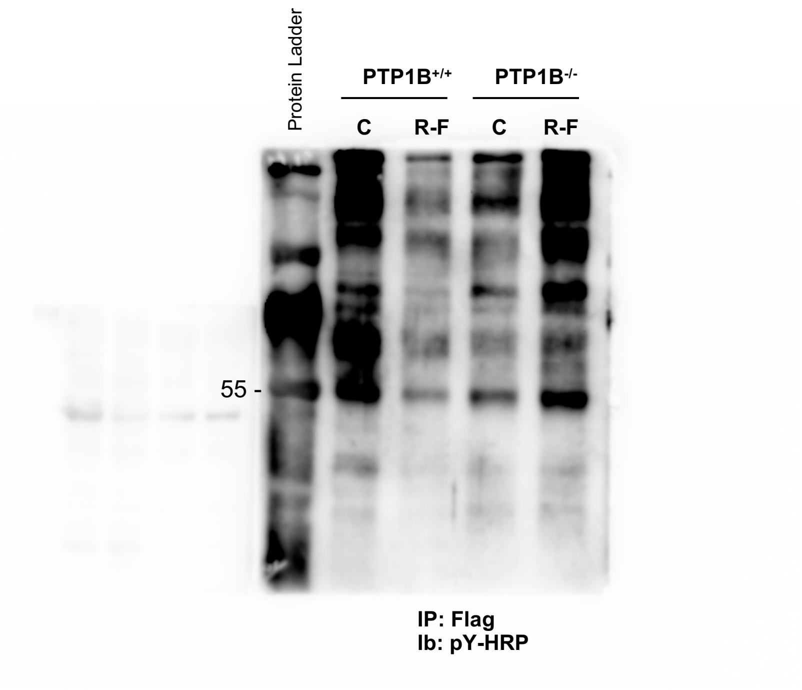

Figure 2.

D.Repeat n.1

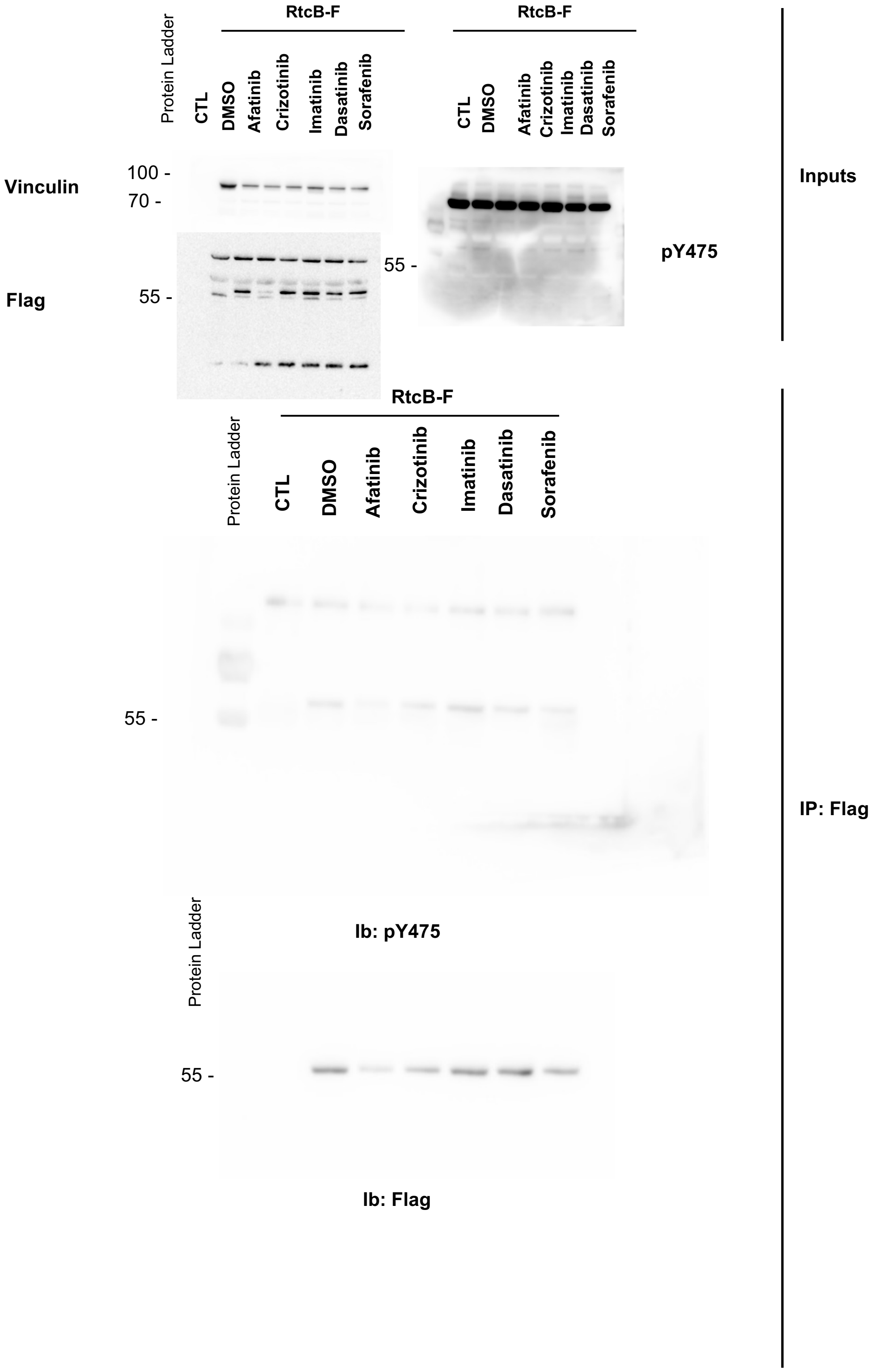

D.Repeat n.2

Figure 2.

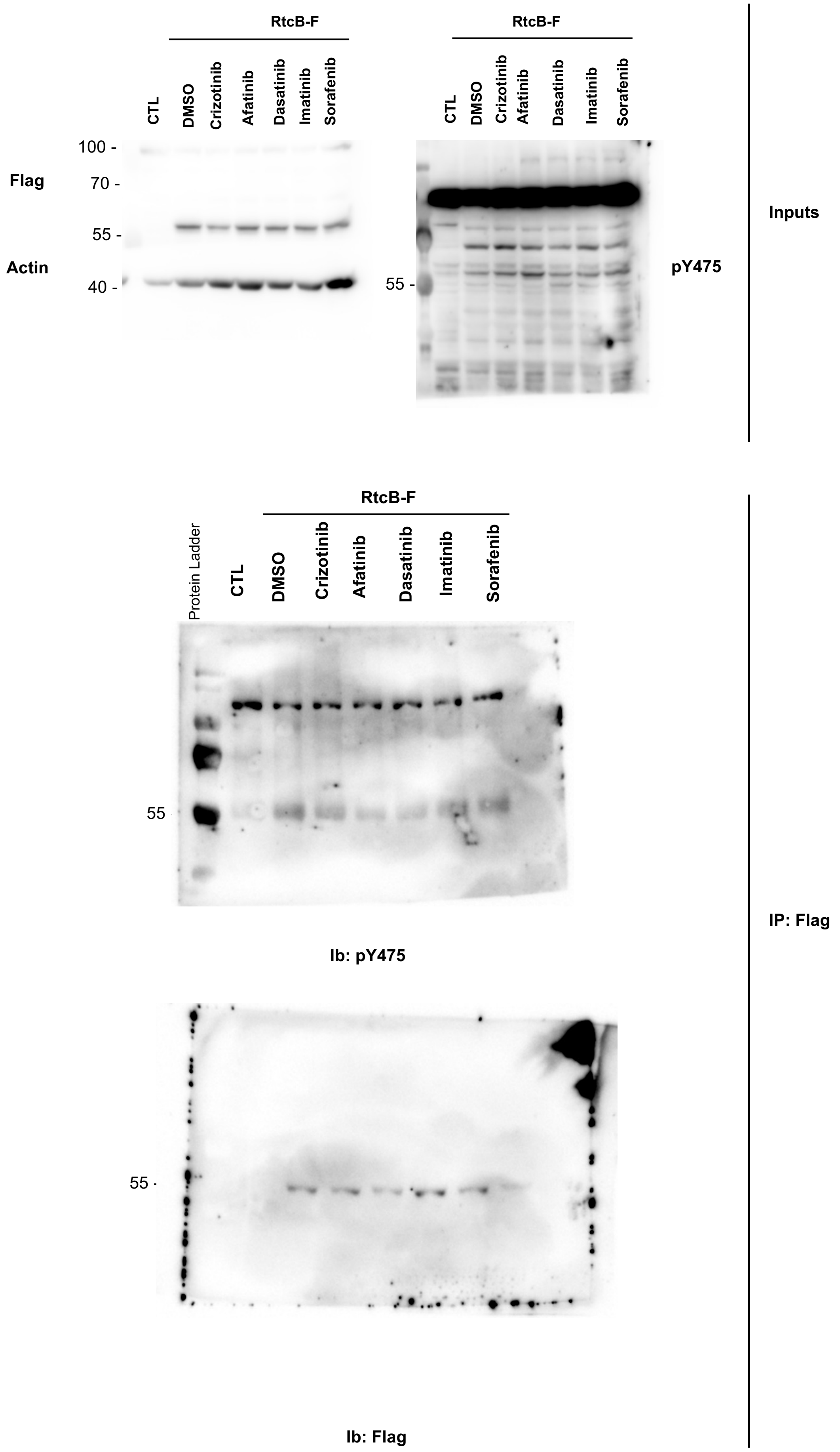

Figure 2.

D.Repeat n.3

In Fig.2D

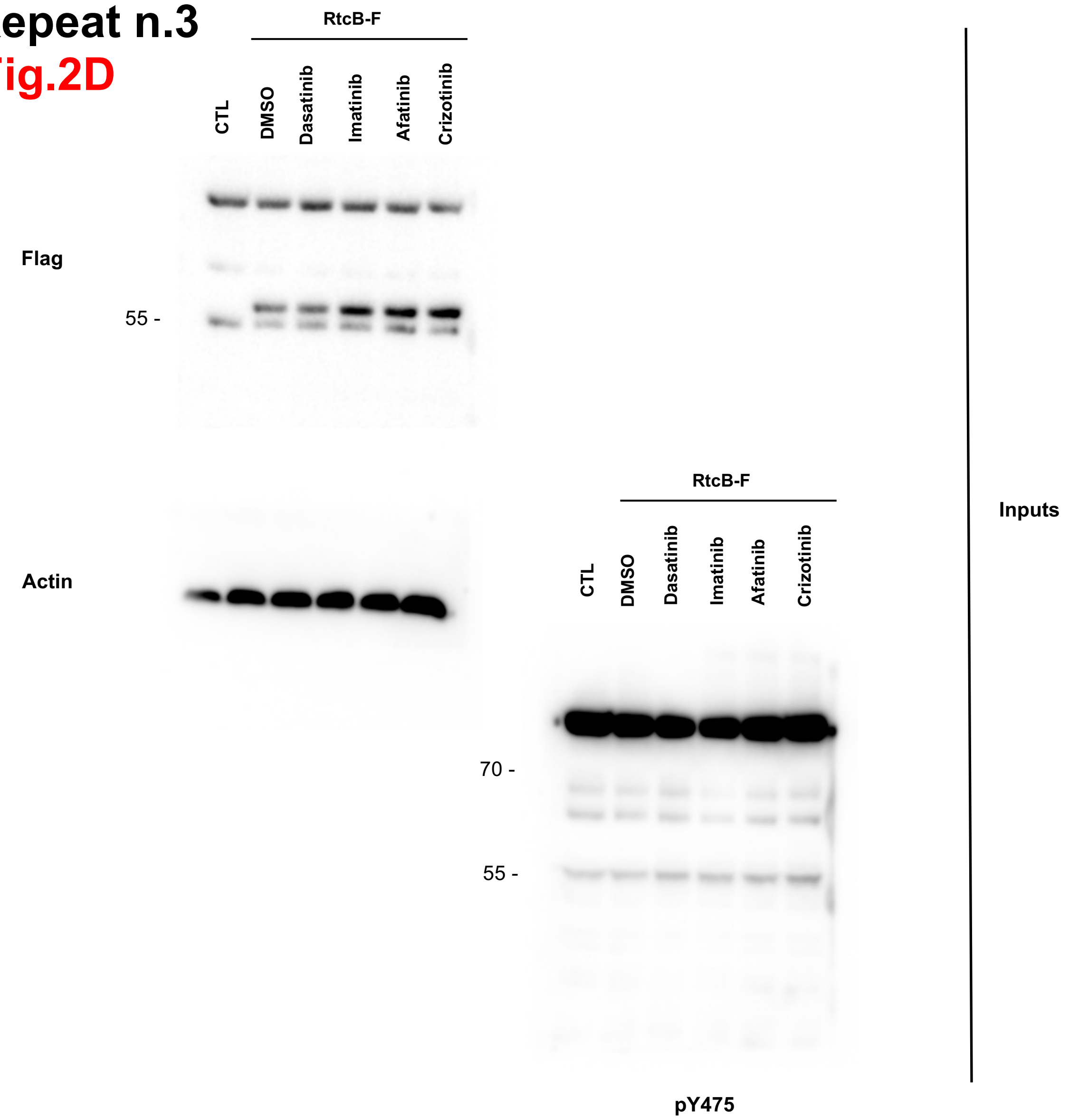

D.Repeat n.3

In Fig.2D

Figure 2.

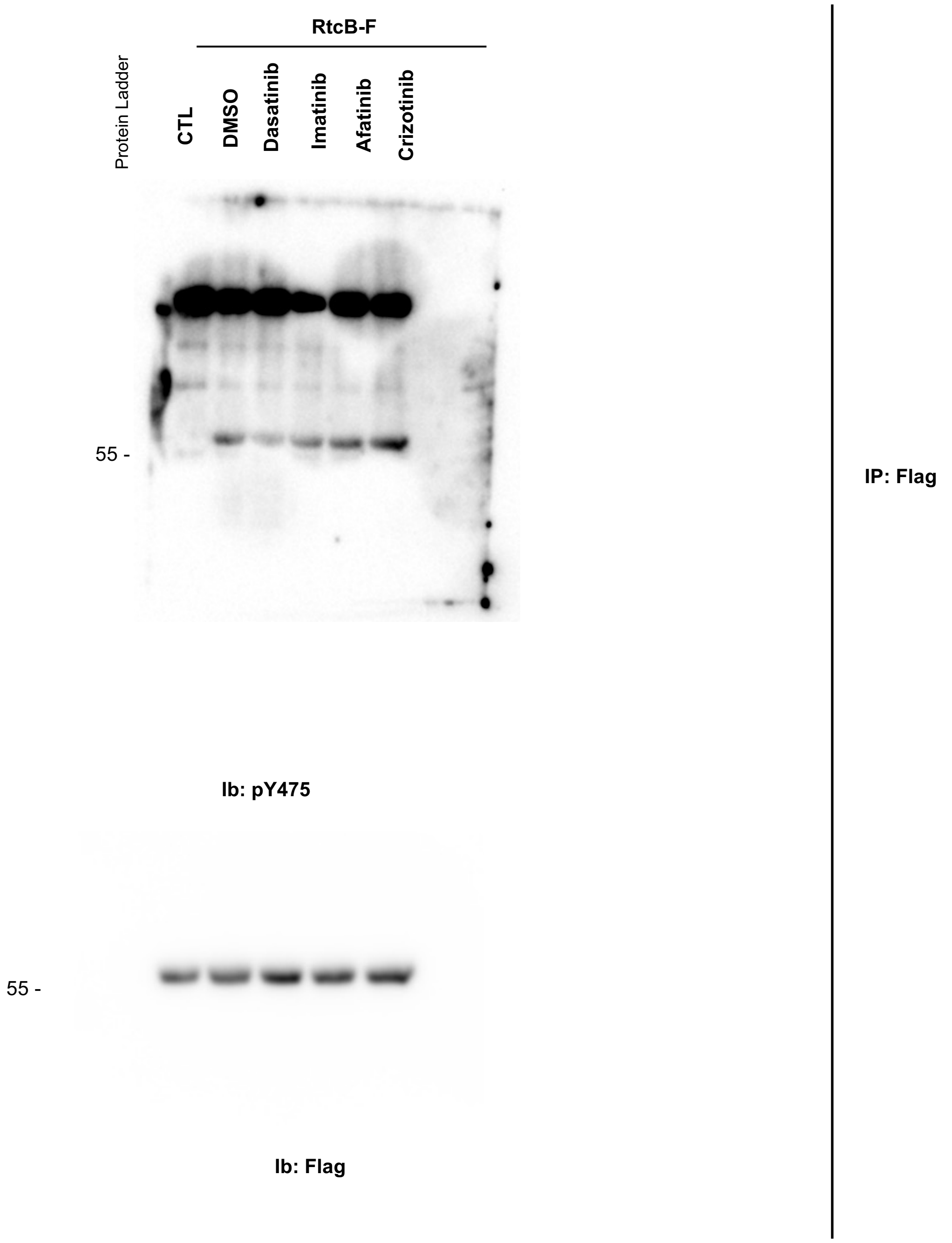

F.Repeat n.1
In Fig.2F

Figure 2.

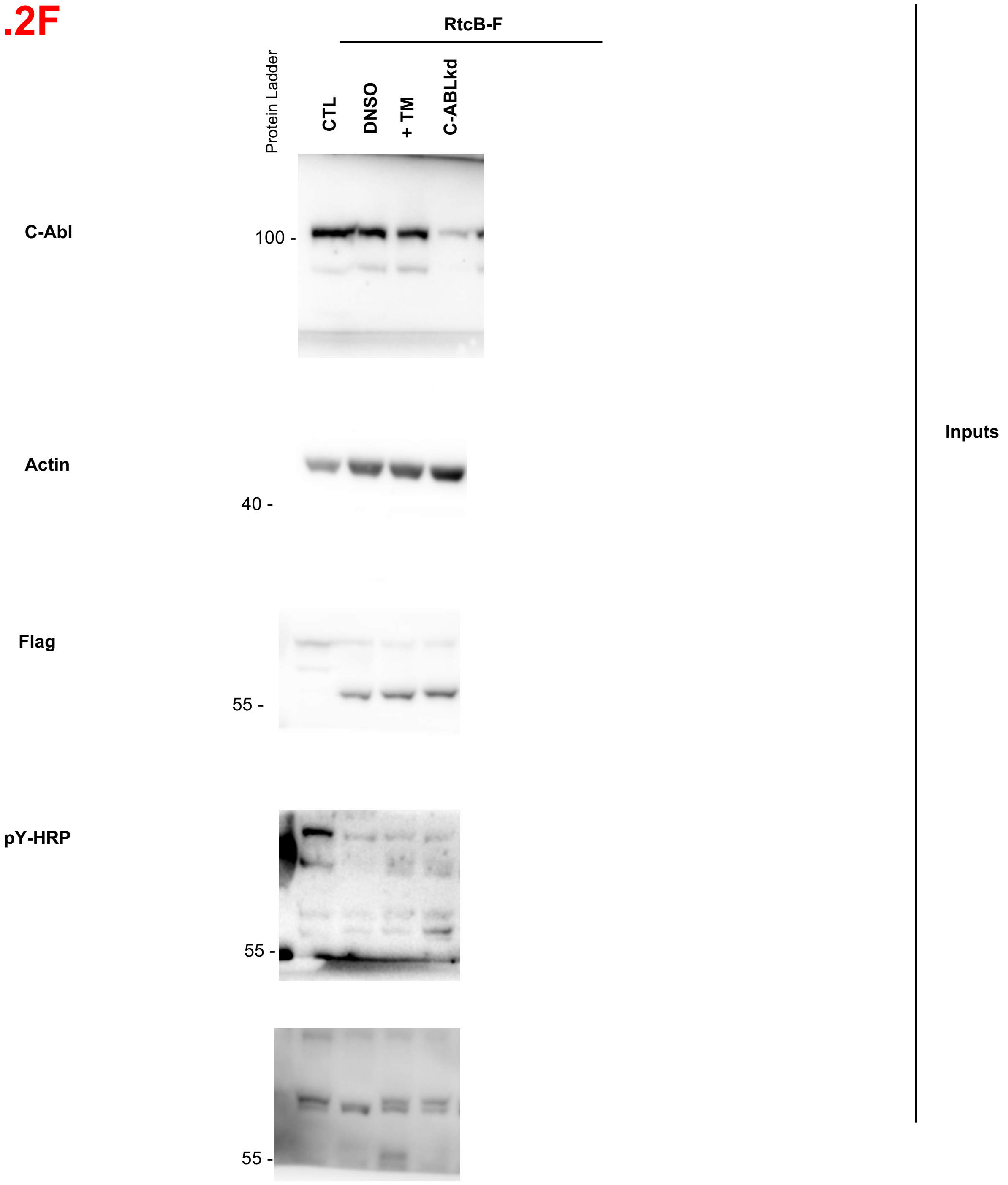

F.Repeat n.1

In Fig.2F

Figure 2.

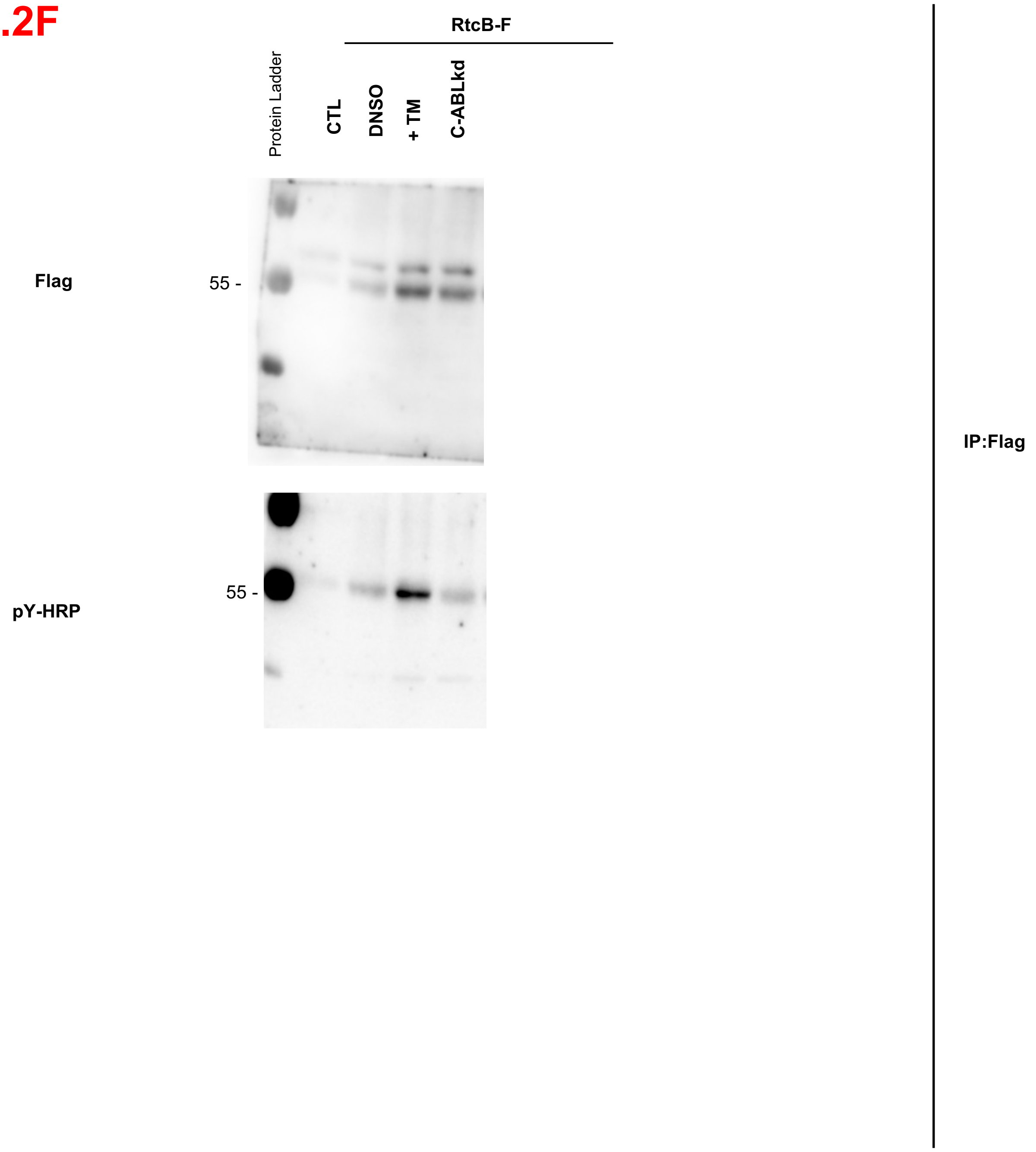

F.Repeat n.2

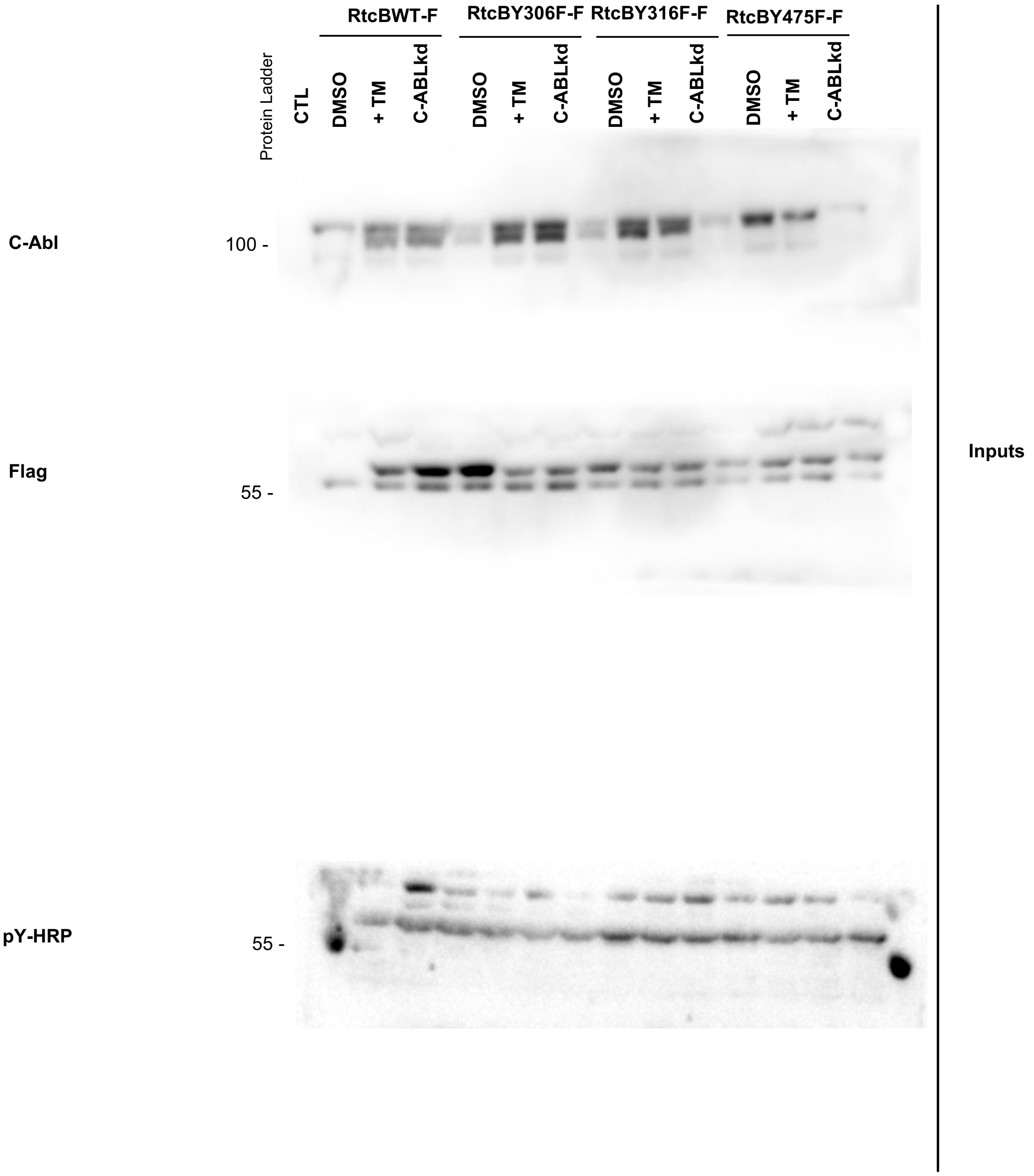
RtcBWT-F
RtcBY306F-F
RtcBY316F-F
RtcBY475F-F
Protein Ladder
CTL
DMSO
+ TM
C-ABLkd
C-Abl
100 -
Flag
55 -
pY-HRP
55 -
Inputs

F.Repeat n.2

Figure 2.

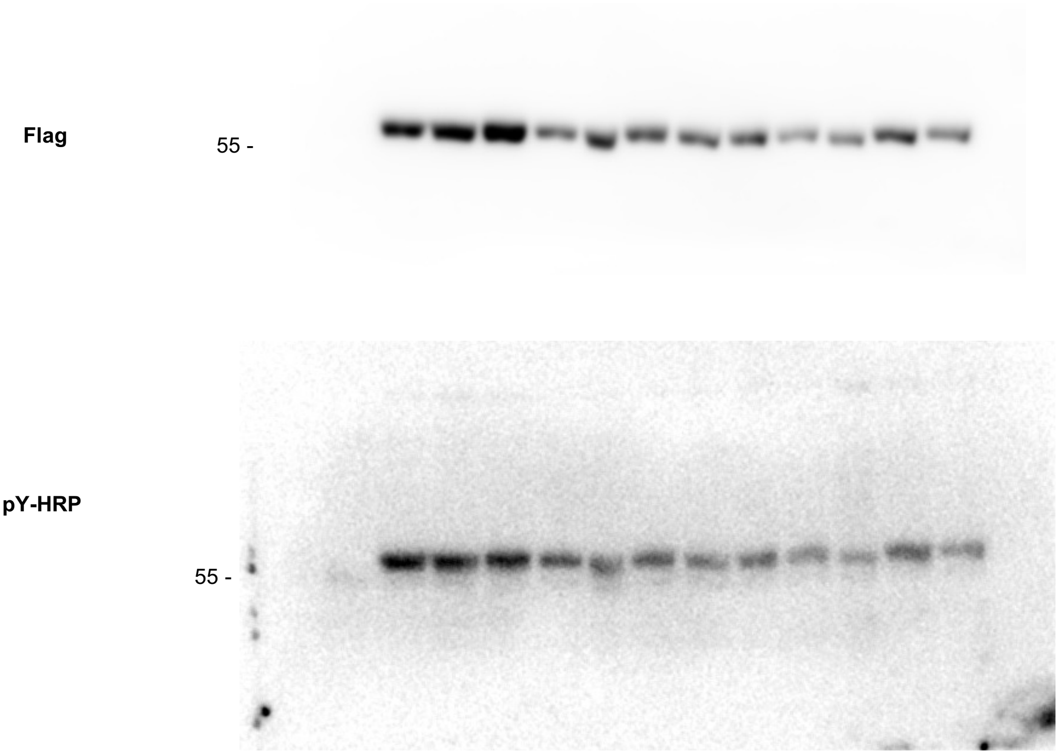

55 -

Figure 2.

F.Repeat n.3

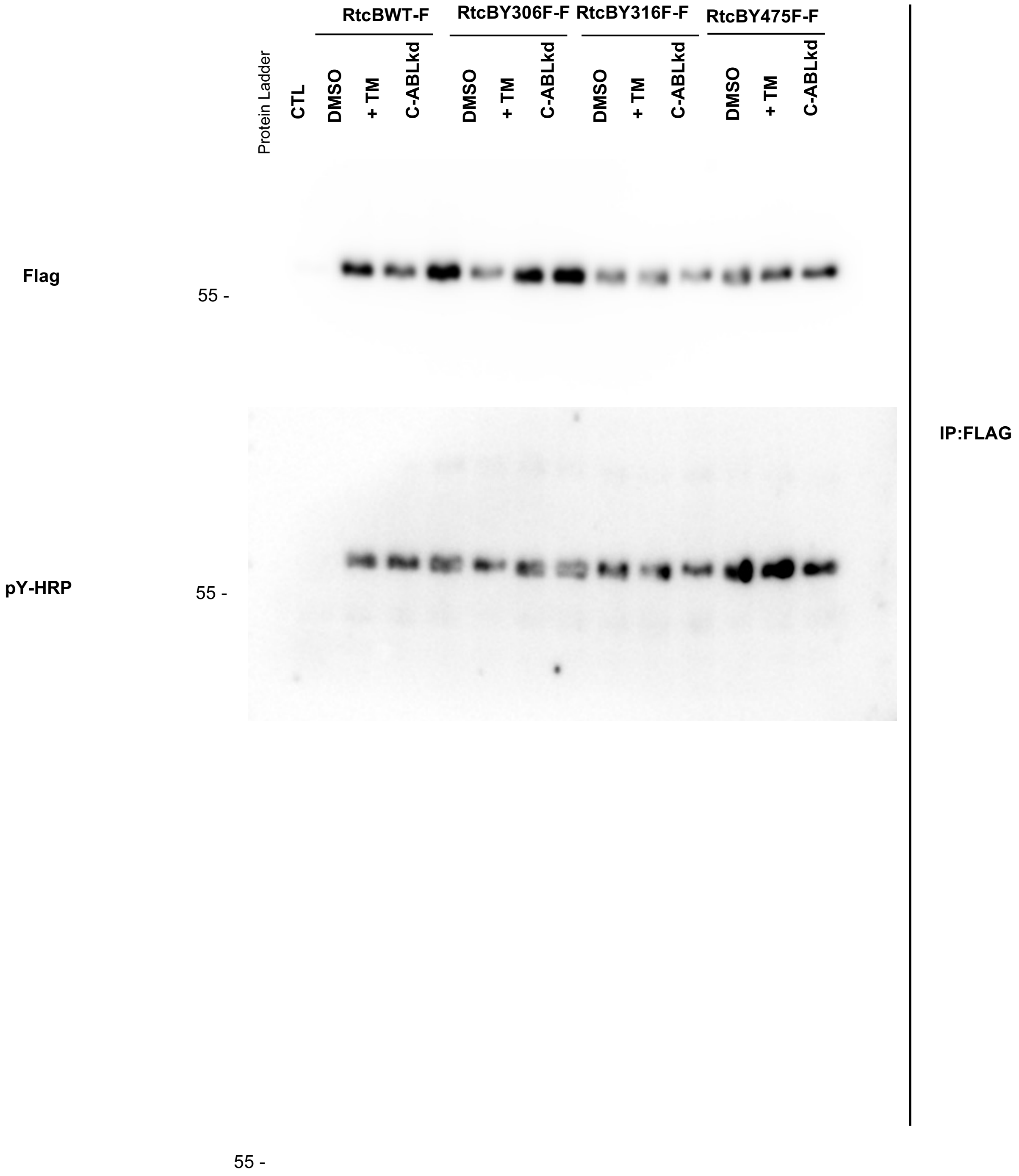

Supplement: Supplementary file 4 [file LSA-2022-01379_SdataF2.zip › Source data Fig2/Source blots fig2.pdf]
