# Supplementary figures and images for: Stress-induced tyrosine phosphorylation of RtcB modulates IRE1 activity and signaling outputs
Source: Life Sci Alliance. 2022 Feb 22;5(5):e202201379. doi: 10.26508/lsa.202201379 (PMC8899846; doi:10.26508/lsa.202201379)

**B**  
**In Fig.S3B**

Supplementary Figure 3.

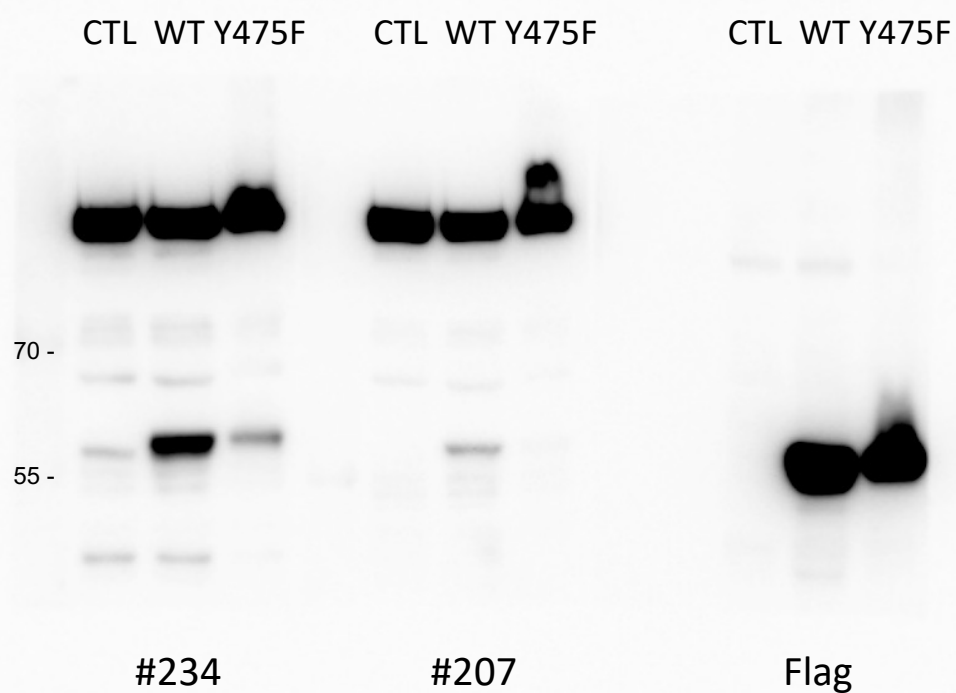

# C

## In Fig.S3C

Supplementary Figure 3.

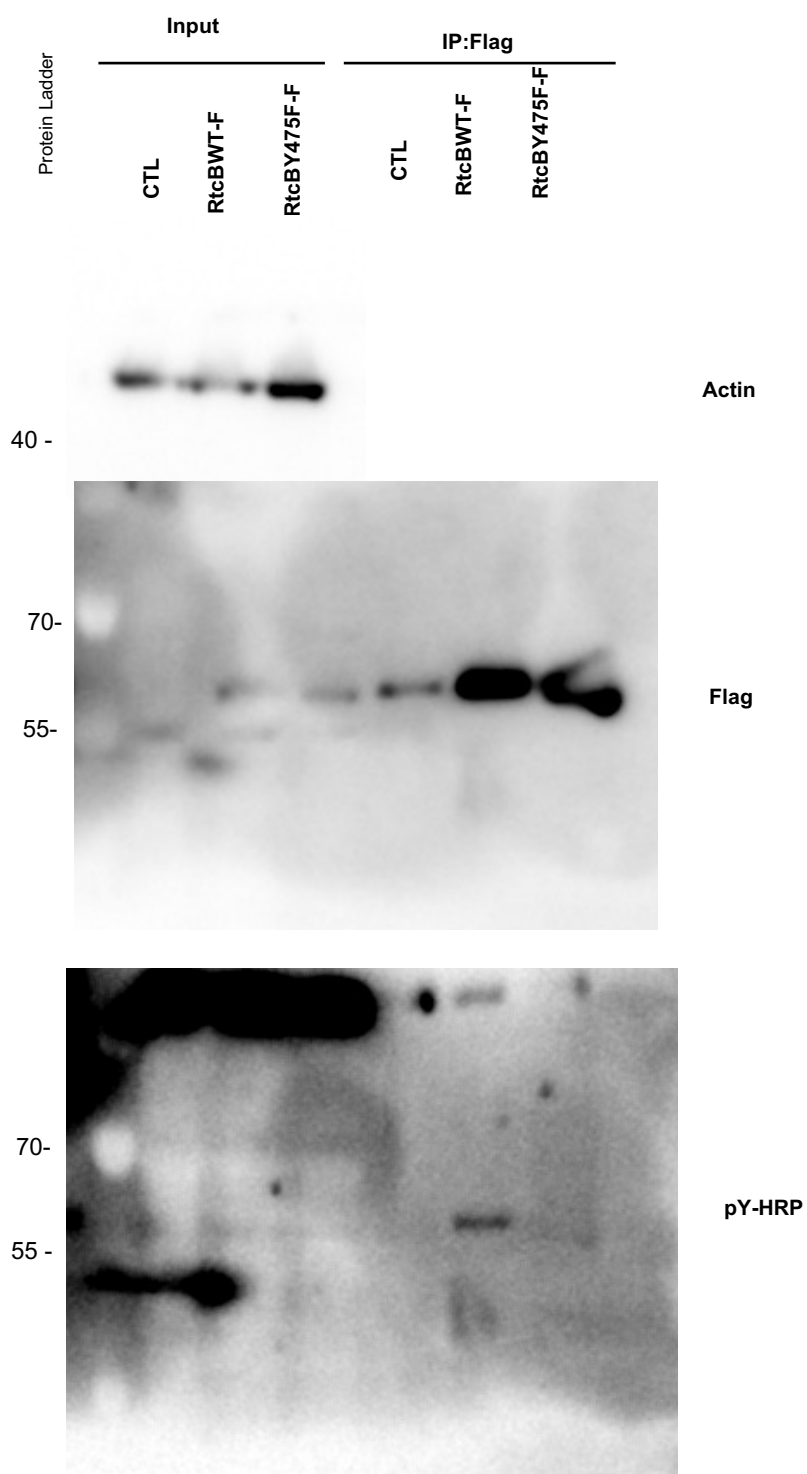

Supplement: Supplementary file 5 [file LSA-2022-01379_SdataFS3.zip › Source data FigS3/Source blots figS3.pdf]

F

Supplementary Figure 7.

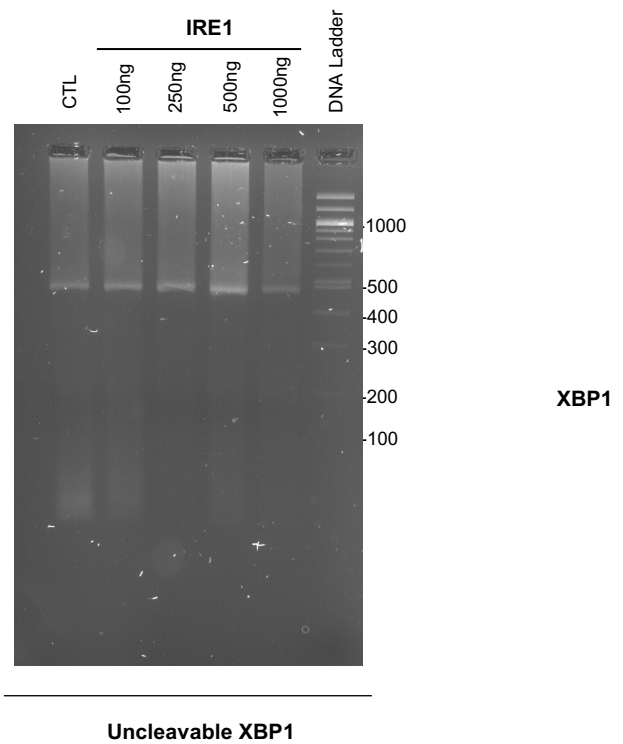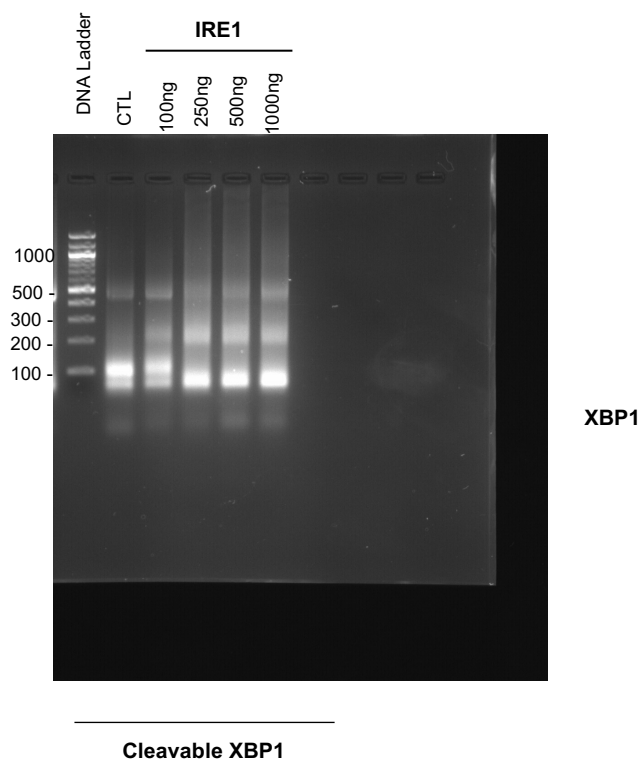

Supplement: Supplementary file 10 [file LSA-2022-01379_SdataFS7.zip › Source data FigS7/Source gels figS7.pdf]

**A In Fig.6A**

Figure 6.

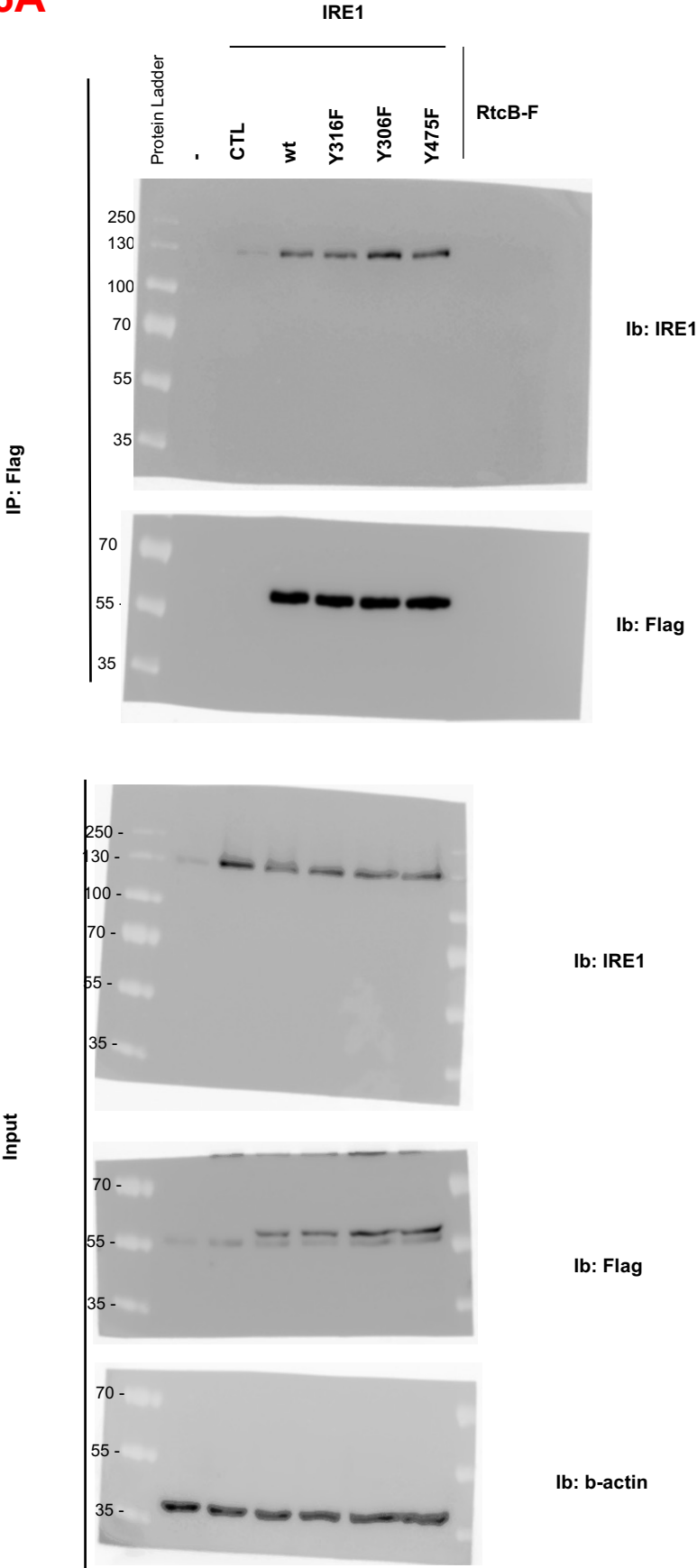

**C.Repeat n.1**  
**In Fig.6C-D**

Figure 6.

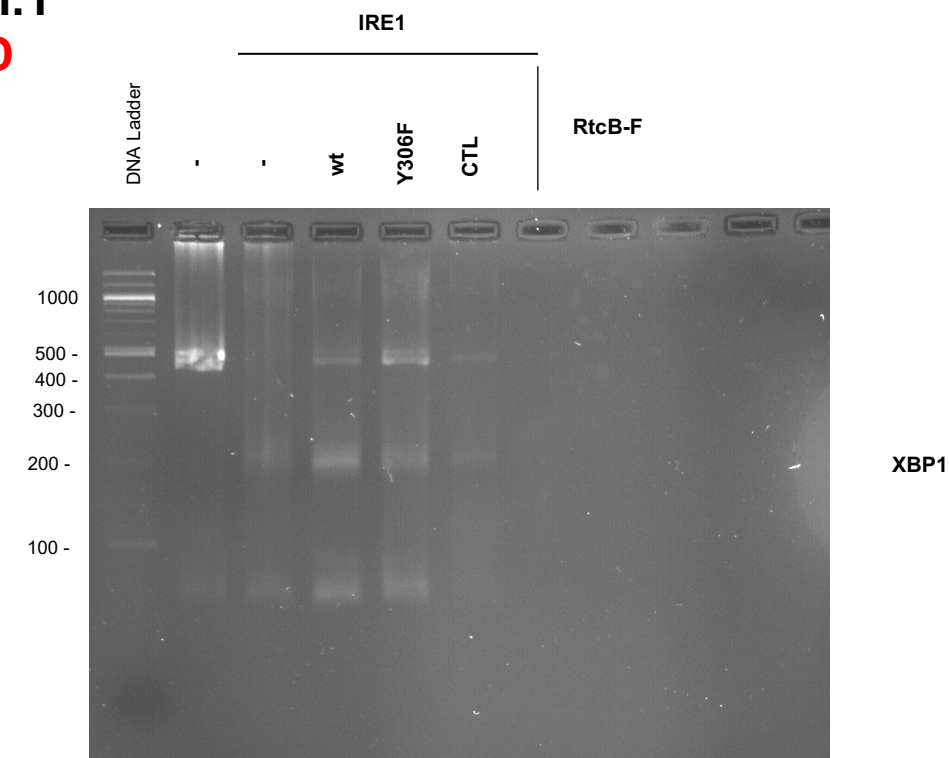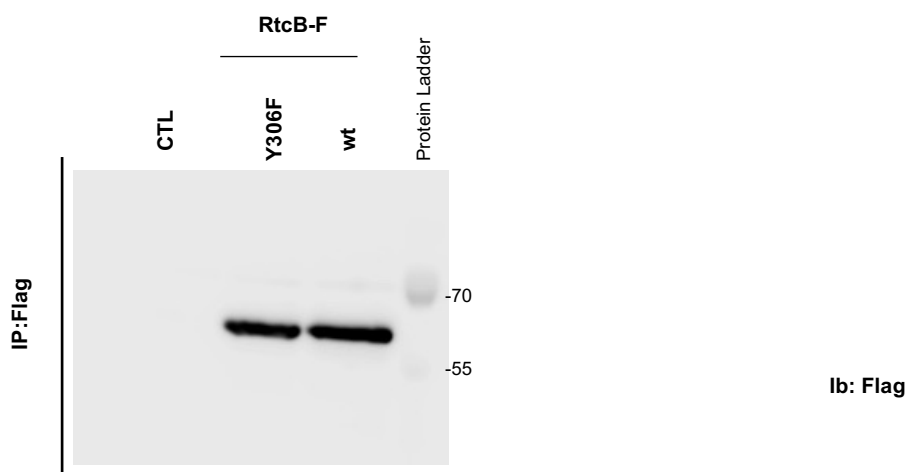

**Flipped orizontally:**

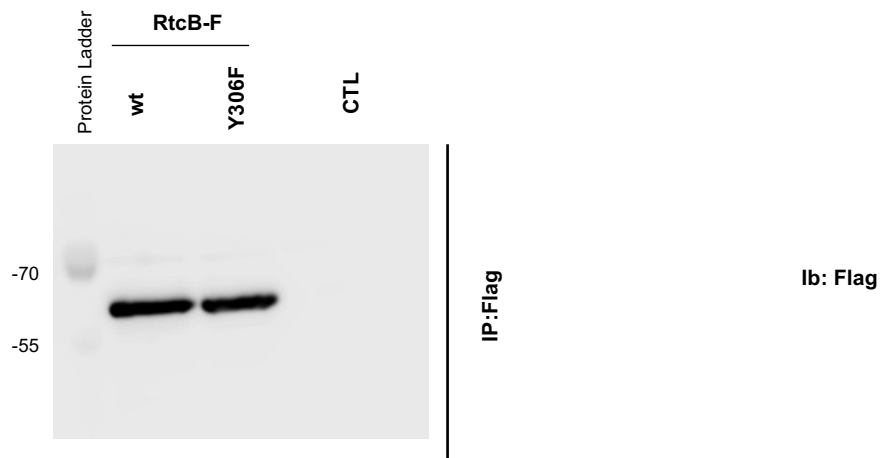

C.Repeat n.2

Figure 6.

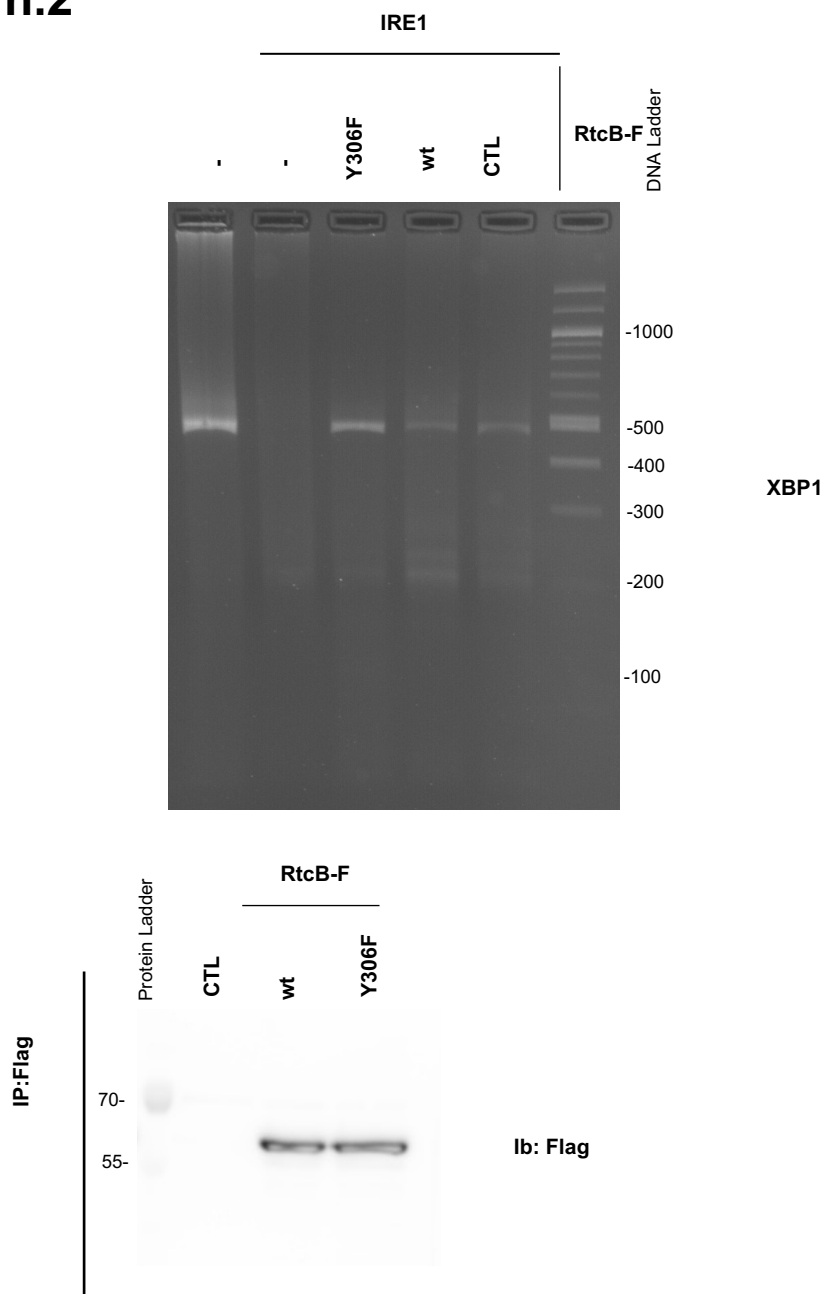

F

Figure 6.

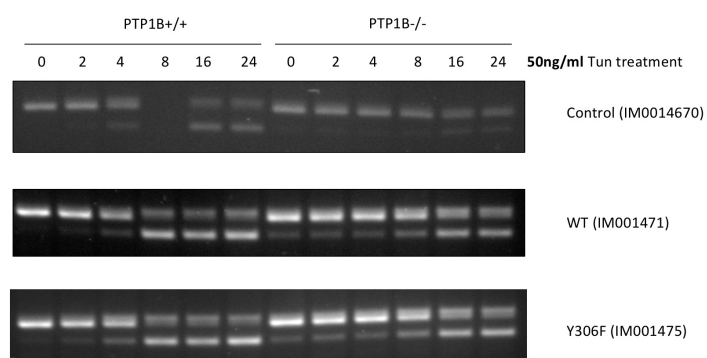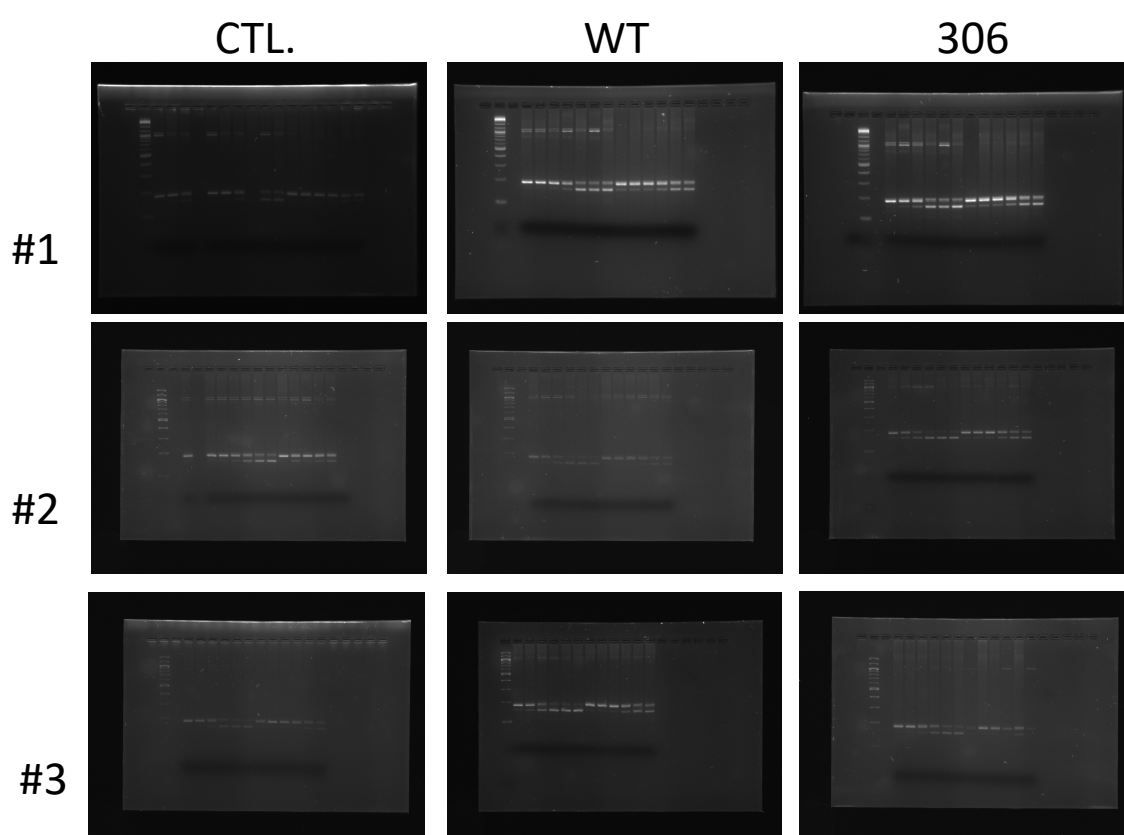

Supplement: Supplementary file 12 [file LSA-2022-01379_SdataF6.zip › Source data Fig6/Source blots&gels fig6.pdf]

**A**

Supplementary Figure 8.

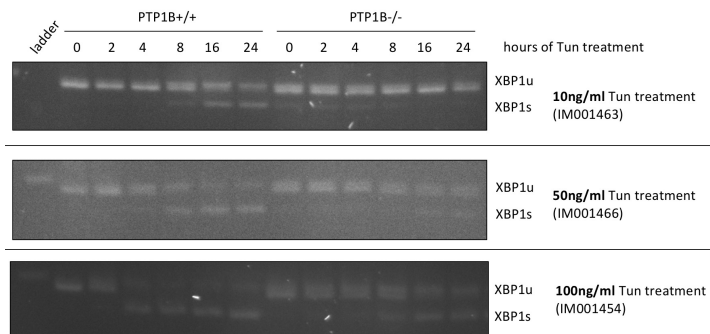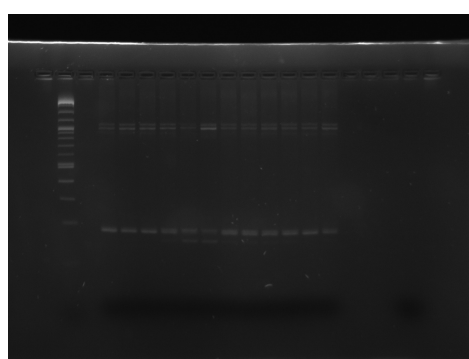

10 ng/ml

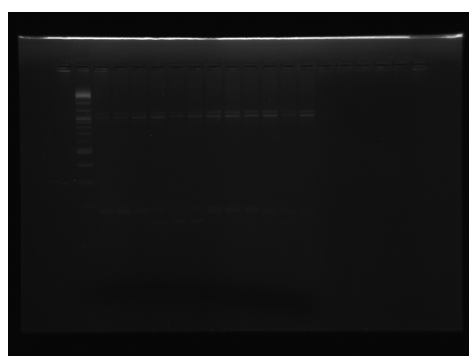

50 ng/ml

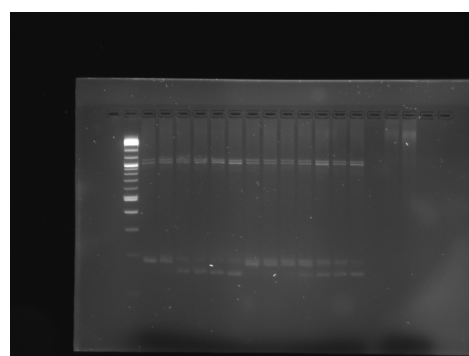

100 ng/ml

D

Supplementary Figure 8.

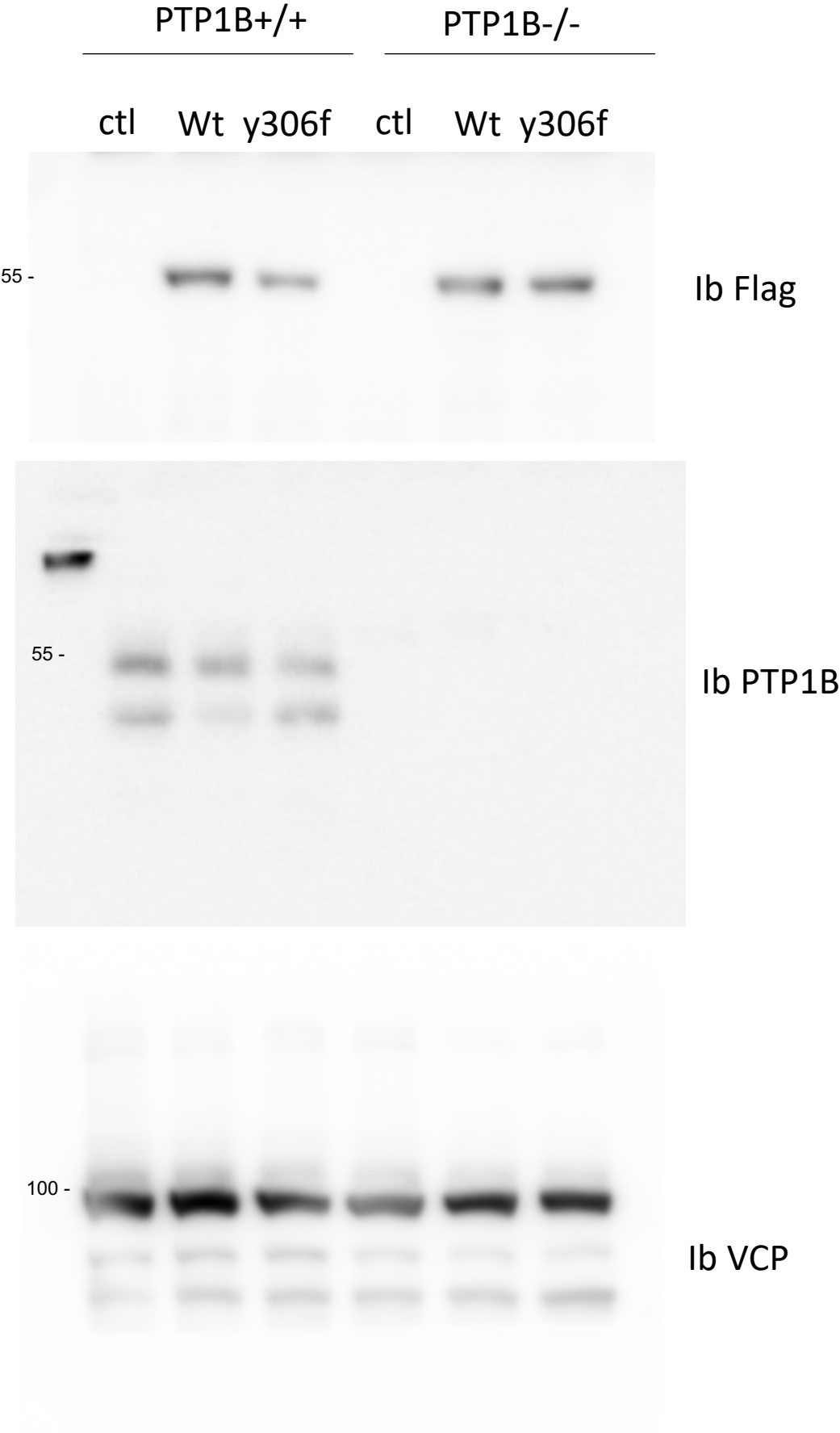

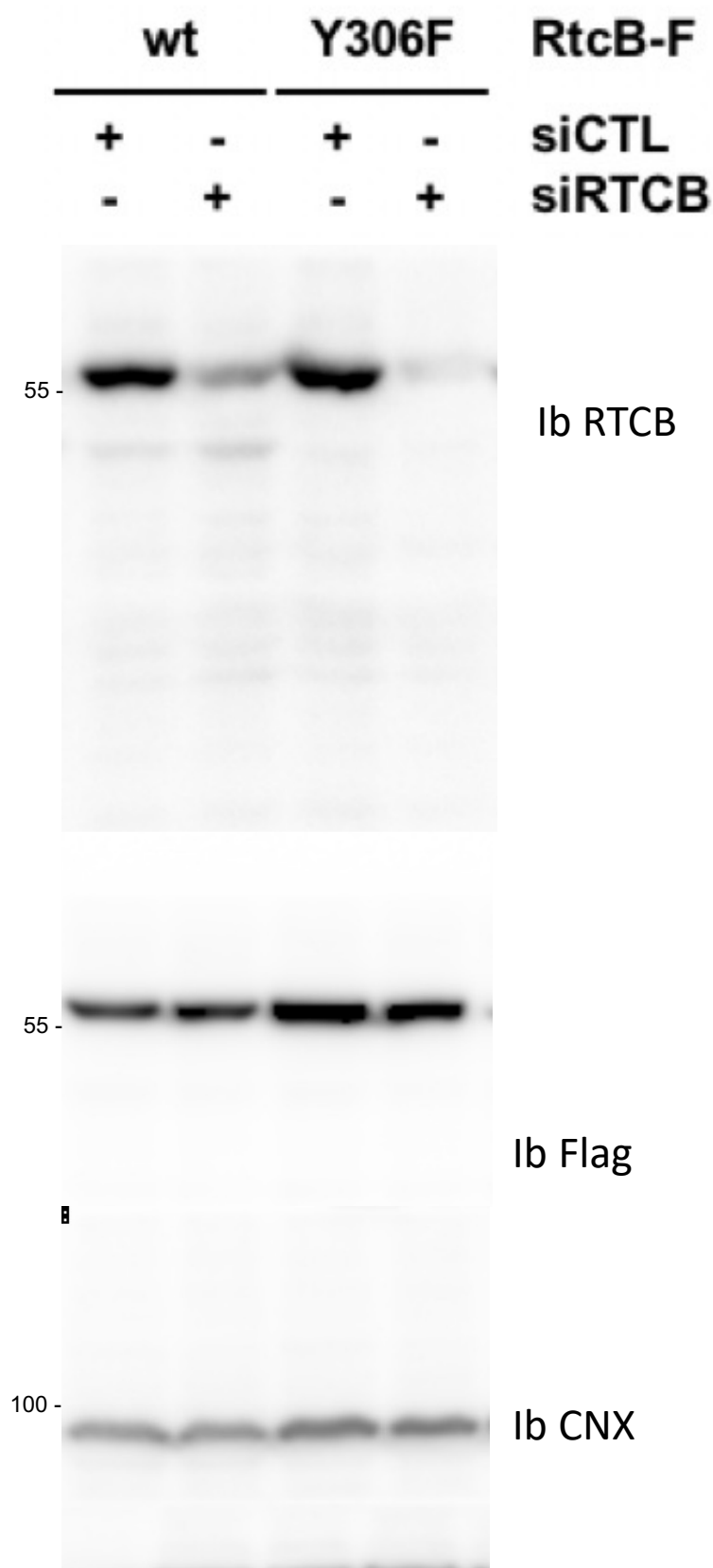

Supplement: Supplementary file 13 [file LSA-2022-01379_SdataFS8.zip › Source data FigS8/Source blots&gels figS8.pdf]
