# Supplementary material for: Stress-induced tyrosine phosphorylation of RtcB modulates IRE1 activity and signaling outputs
Source: Life Sci Alliance. 2022 Feb 22;5(5):e202201379. doi: 10.26508/lsa.202201379 (PMC8899846; doi:10.26508/lsa.202201379)
Supplement: Supplementary file 6 [file LSA-2022-01379_TableS2.pdf]

Table S2: pKa calculations and percentage of a residue being "buried" within the protein for the tyrosines 306, 316 and 475. For each RtcB system, the final structure after the 200ns simulation was analyzed using the online server PDB2PQR - PROPKA. "Buried" residue is the opposite term of the "surface" residue, that is solvent-exposed.

		RtcB system							
	Tyrosine	non-pY	pY306	pY316	pY475	pY306-316	pY306-475	pY316-475	pY306-316-475
pKa	306	13,55	12,8	13,66	13,51	-	11,13	13,42	10,17
	316	12,96	13,34	12,13	13,04	-	13,53	12	12,03
	475	13,51	13,24	13,63	9,53	13,35	9,71	9,81	10,42
% "buried" residue	306	100%	100%	100%	100%	-	85%	100%	15%
	316	100%	100%	93%	100%	-	100%	100%	100%
	475	99%	84%	92%	100%	96%	85%	67%	77%
