# Supplementary material for: Stress-induced tyrosine phosphorylation of RtcB modulates IRE1 activity and signaling outputs
Source: Life Sci Alliance. 2022 Feb 22;5(5):e202201379. doi: 10.26508/lsa.202201379 (PMC8899846; doi:10.26508/lsa.202201379)
Supplement: Supplementary file 7 [file LSA-2022-01379_TableS3.pdf]

Supplementary Table 3.

Table S3: For each RtcB system, solvent accessibility surface area (SASA) in nm^2 of each tyrosine residue (306, 316, 475) was calculated throughout the 200ns simulation using the Gromacs 3.0 package. The values at the starting point of 0ns and the final point of 200ns are presented in the table.

		non-pY		pY306		pY316		pY475		pY306-316		pY306-475		pY316-475		pY306-316-475	
	Tyr	0ns	200ns	0ns	200ns	0ns	200ns	0ns	200ns	0ns	200ns	0ns	200ns	0ns	200ns	0ns	200ns
SASA (nm^2)	**306**	3,473	3,606	4,277	4,444	3,541	3,548	3,658	3,443	4,28	4,3	4,419	4,093	3,547	3,716	4,219	4,541
	**316**	3,593	4,077	3,443	3,591	4,43	4,396	3,747	3,258	4,371	4,37	3,351	3,604	4,529	4,222	4,504	4,198
	**475**	3,534	3,543	3,901	3,738	3,592	3,865	4,273	4,19	3,604	3,753	4,389	4,415	4,317	4,391	4,509	4,292
