# Supplementary material for: Stress-induced tyrosine phosphorylation of RtcB modulates IRE1 activity and signaling outputs
Source: Life Sci Alliance. 2022 Feb 22;5(5):e202201379. doi: 10.26508/lsa.202201379 (PMC8899846; doi:10.26508/lsa.202201379)

Figure 4.

B **In Fig.4B**

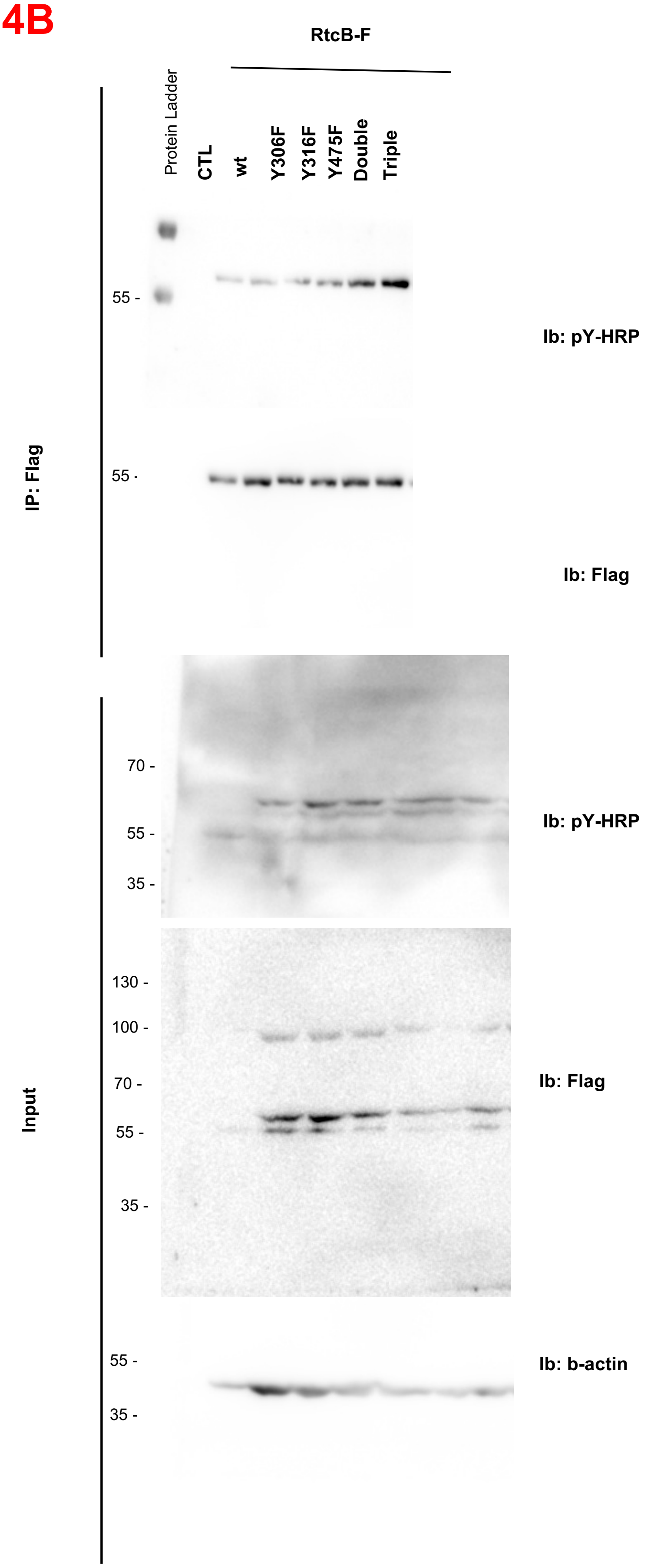

C.Repeat n.1

Figure 4.

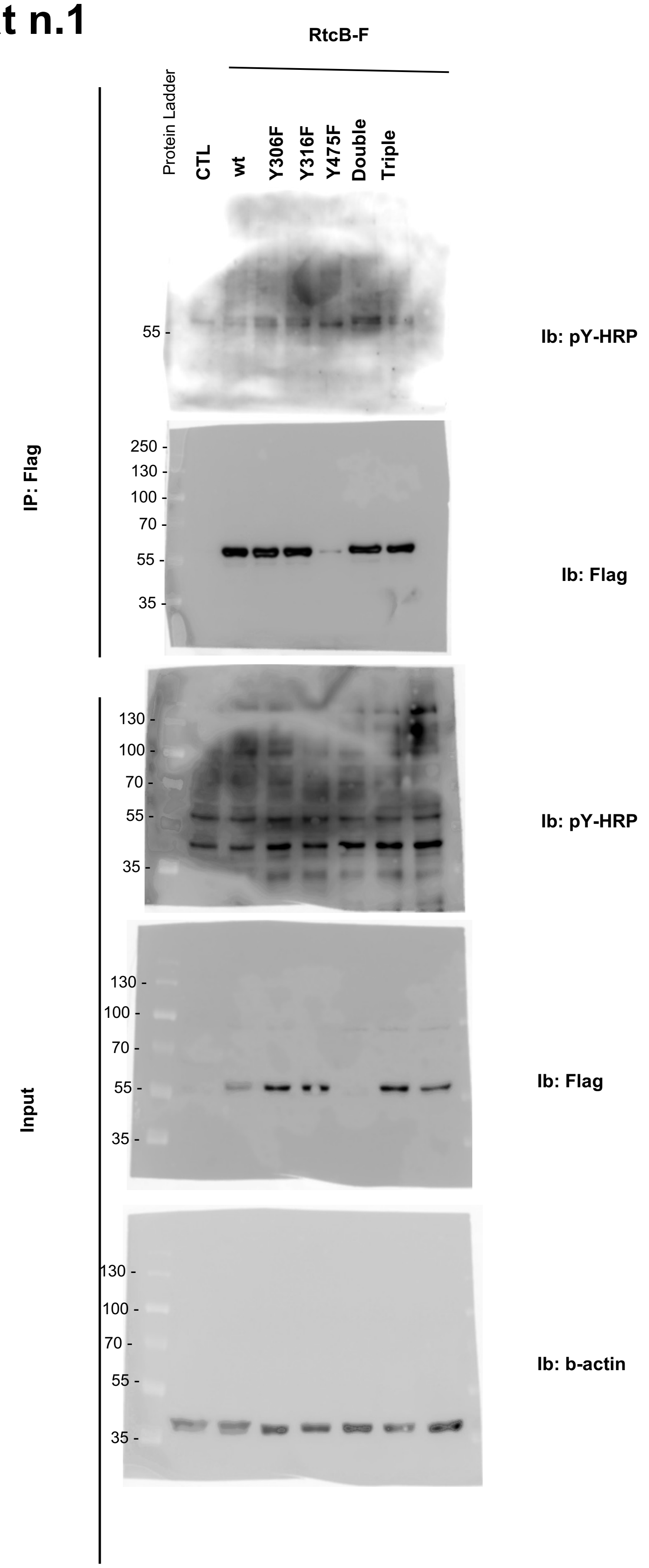

C.Repeat n.2

Figure 4.

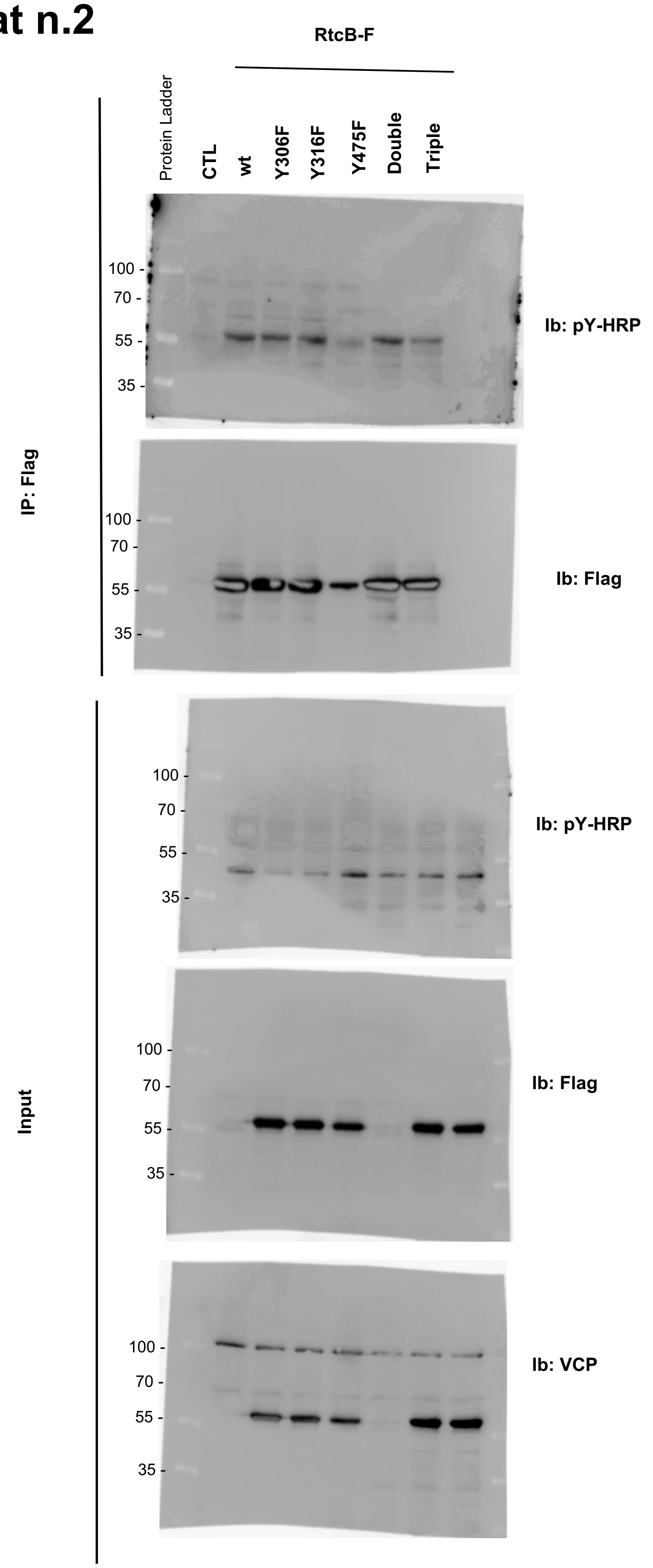

C.Repeat n.3

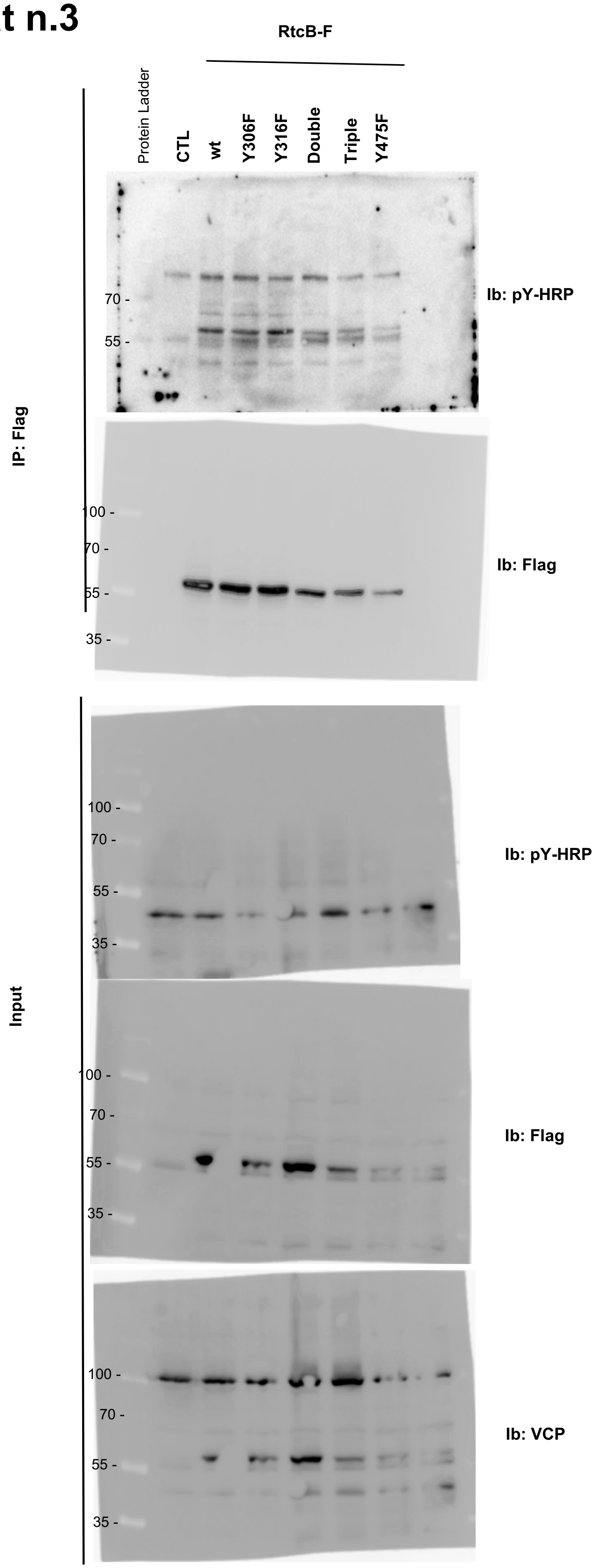
RtcB-F
Protein Ladder
CTL
wt
Y306F
Y316F
Double
Triple
Y475F
IP: Flag
Input
70 -
55 -
100 -
35 -
Ib: pY-HRP
Ib: Flag
Ib: pY-HRP
Ib: Flag
Ib: VCP

D.Repeat n.1.1 In Fig.4D and S7A

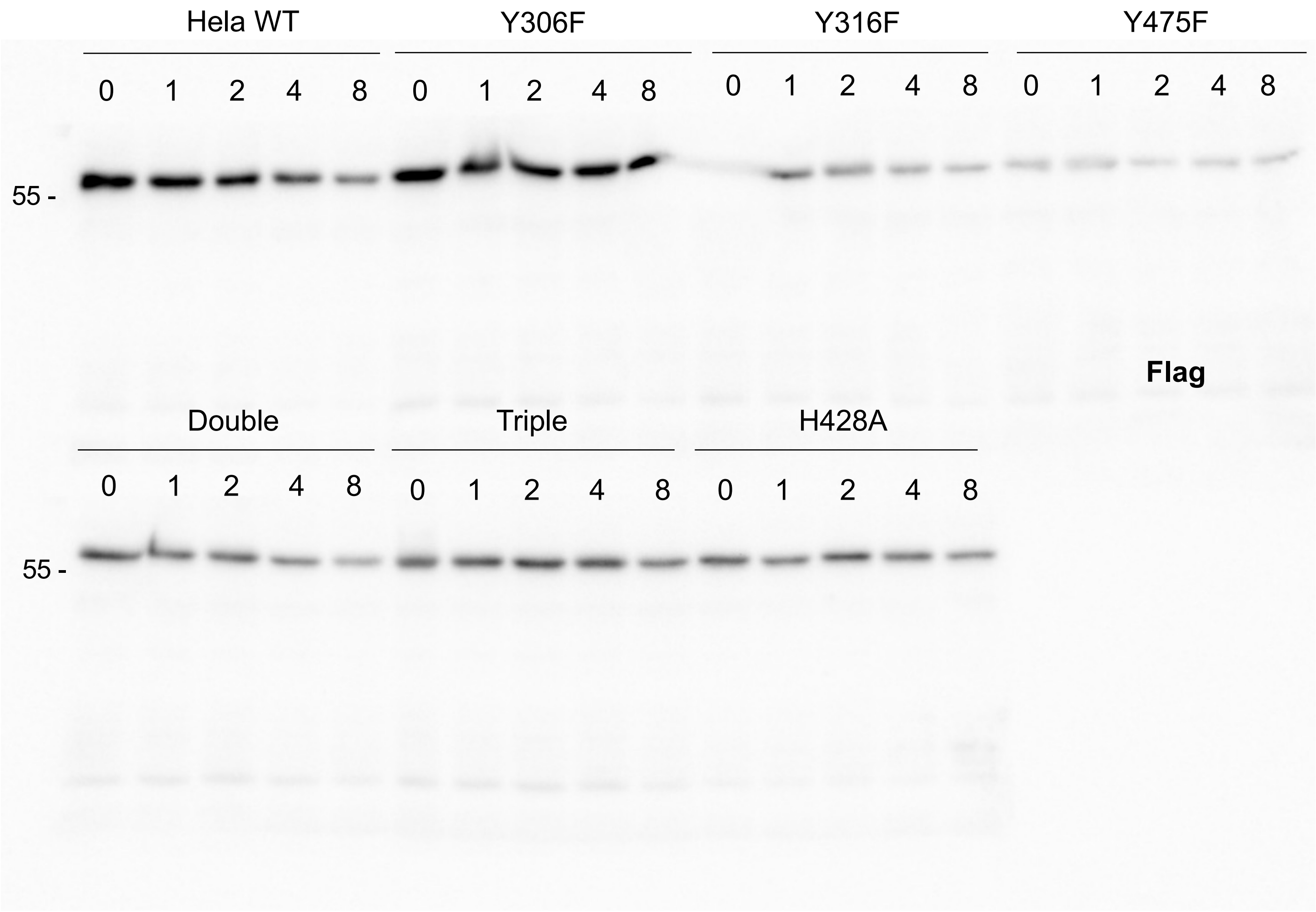

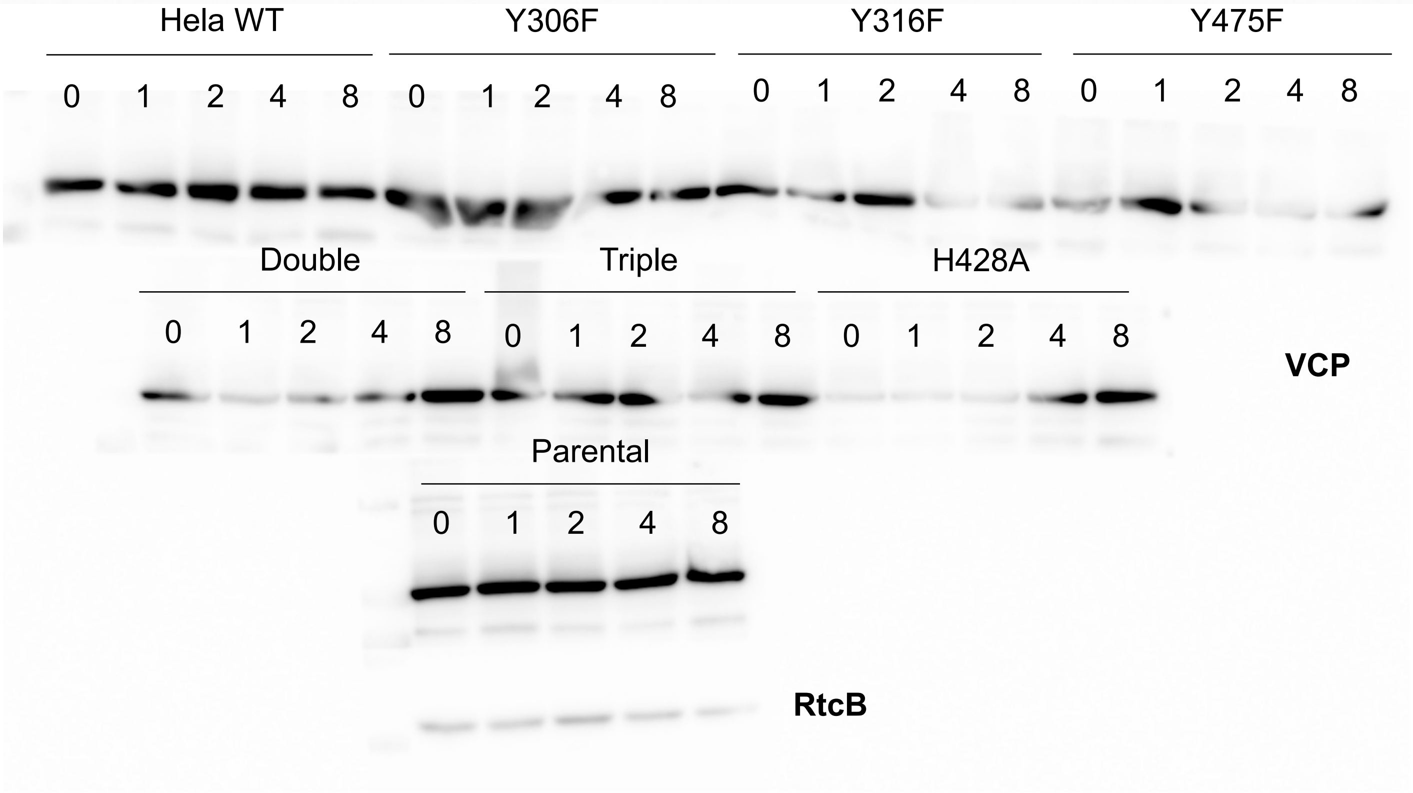

Figure 4.

D.Repeat n.1.2 In Fig.4D and S7A

Run a second time

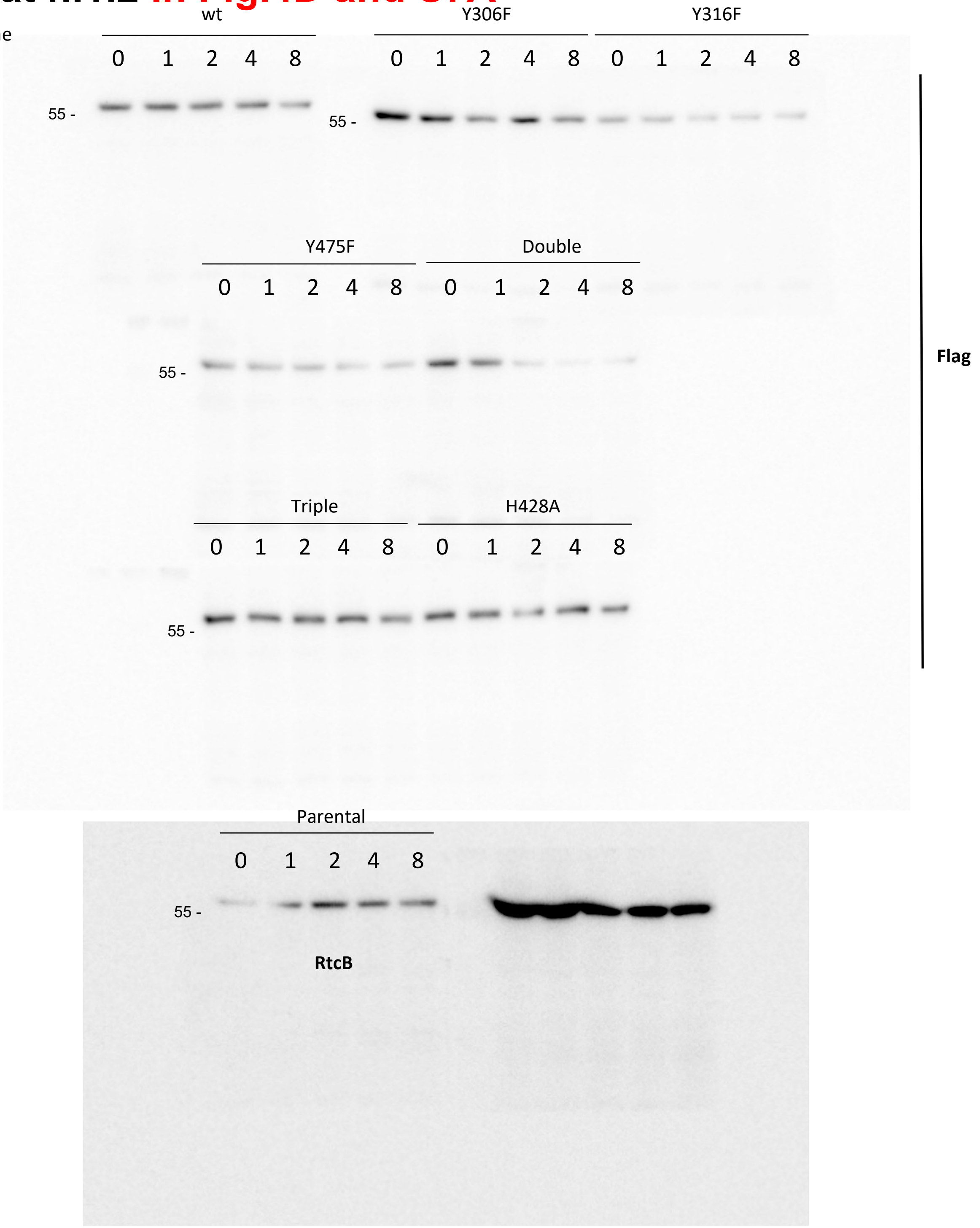

D.Repeat n.1.2 In Fig.4D and S7A

Figure 4.

Run a second time

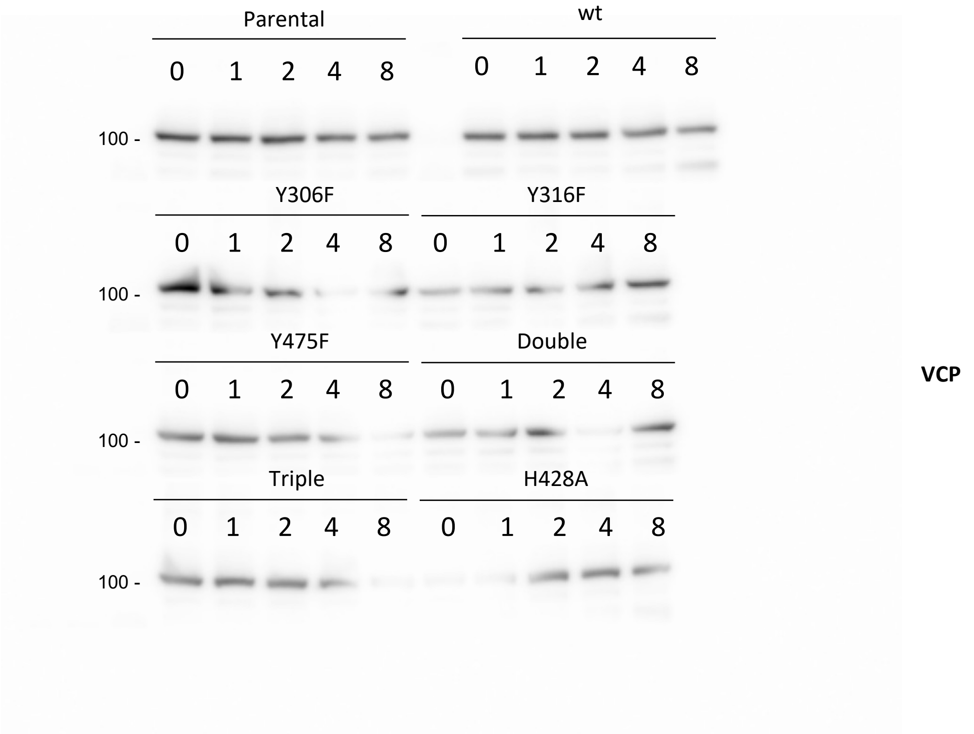

D.Repeat n.2

Figure 4.

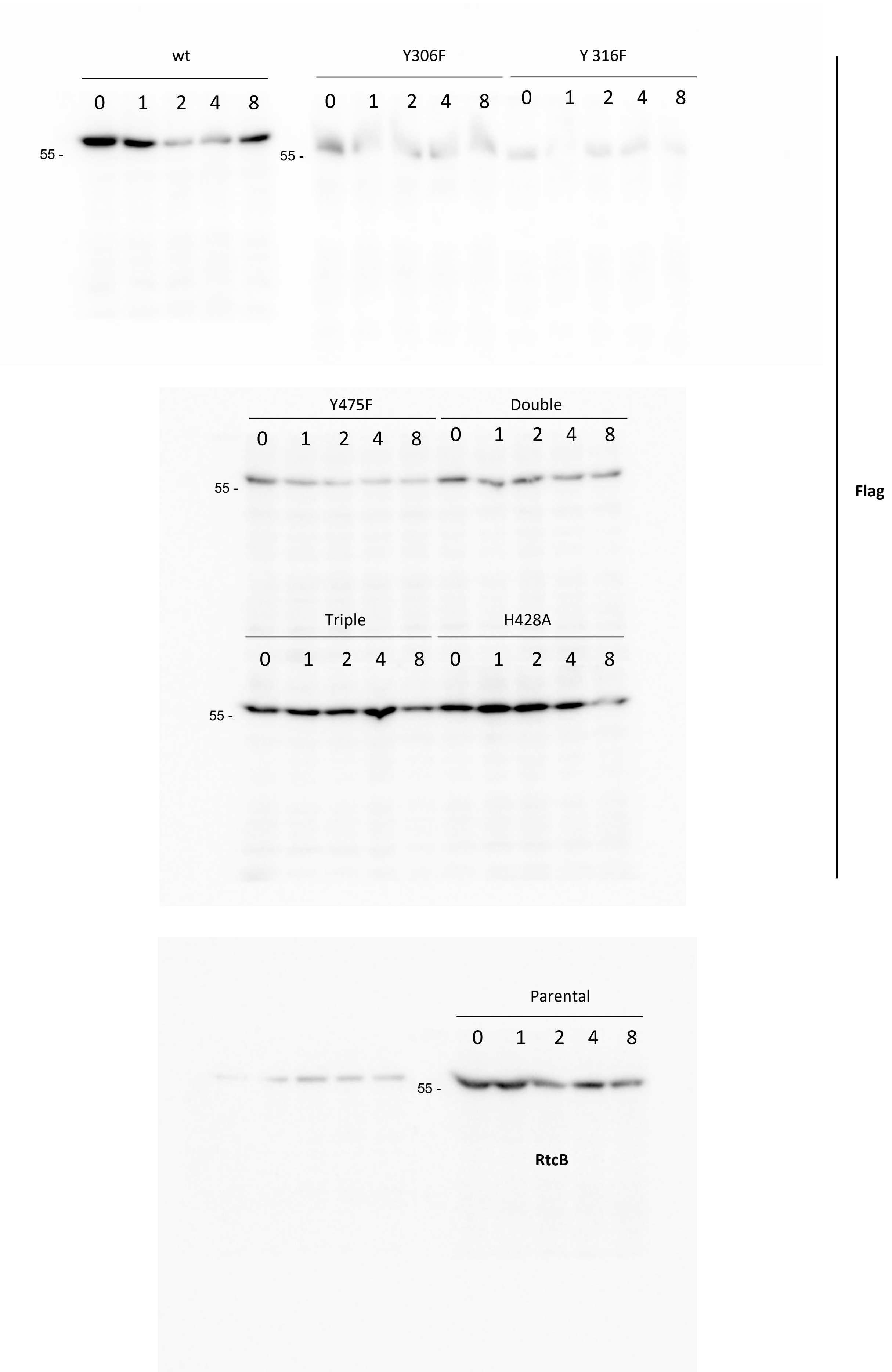

D.Repeat n.2

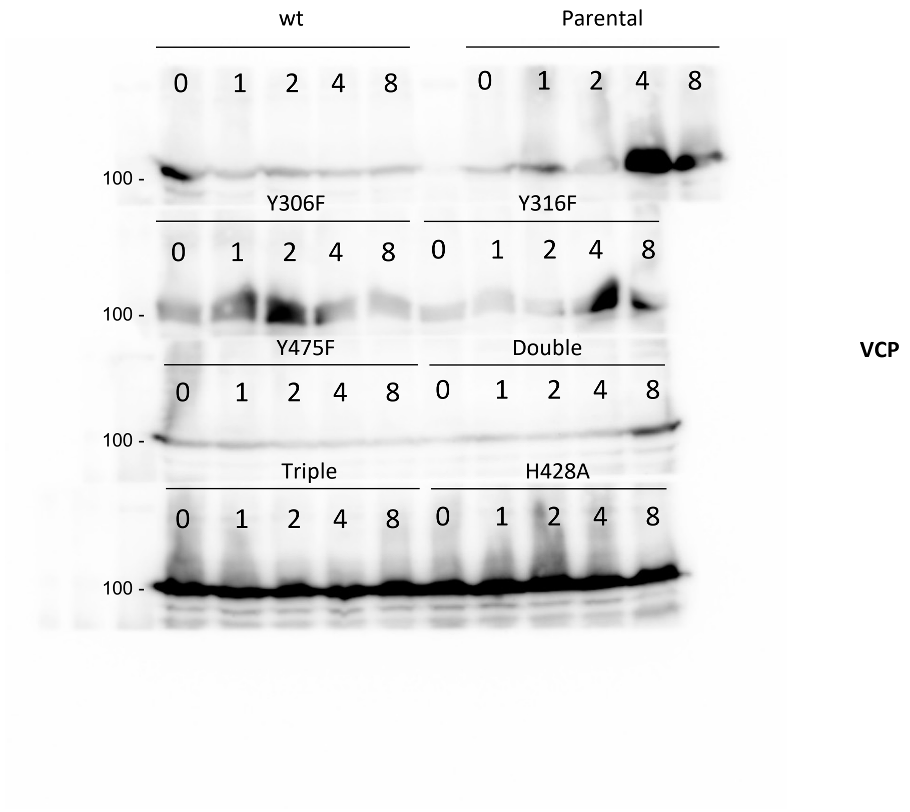
wt
Parental
0 1 2 4 8
0 1 2 4 8
100 -
Y306F
Y316F
0 1 2 4 8
0 1 2 4 8
100 -
Y475F
Double
0 1 2 4 8
0 1 2 4 8
100 -
Triple
H428A
0 1 2 4 8
0 1 2 4 8
100 -
VCP

D.Repeat n.3

In Fig.4D and S7A

Figure 4.

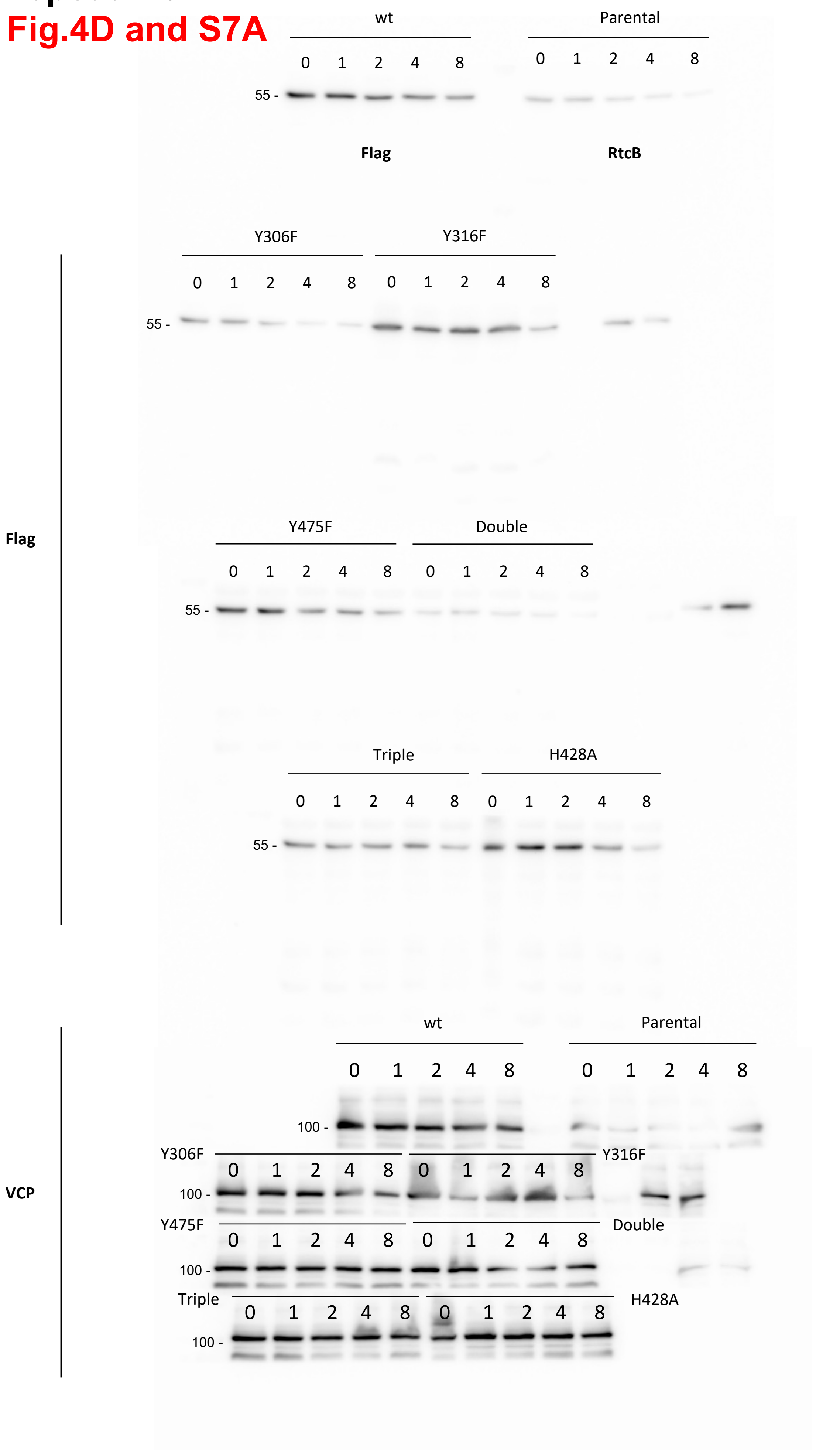

Supplement: Supplementary file 8 [file LSA-2022-01379_SdataF4.zip › Source data Fig4/Source blots fig4.pdf]
