# Supplementary material for: Stress-induced tyrosine phosphorylation of RtcB modulates IRE1 activity and signaling outputs
Source: Life Sci Alliance. 2022 Feb 22;5(5):e202201379. doi: 10.26508/lsa.202201379 (PMC8899846; doi:10.26508/lsa.202201379)

A.Repeat n.1

Supplementary Figure 6.

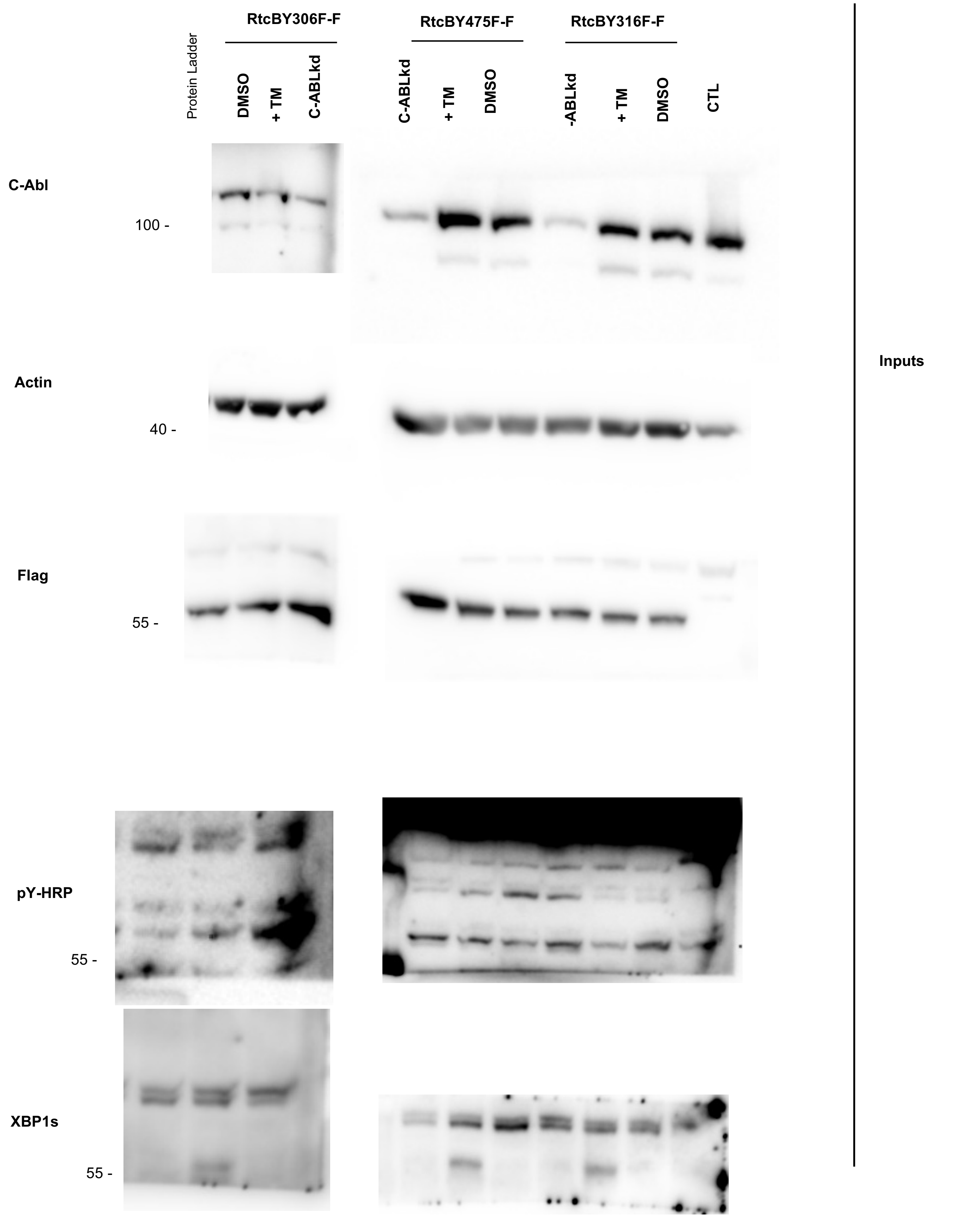

A.Repeat n.1

Supplementary Figure 6.

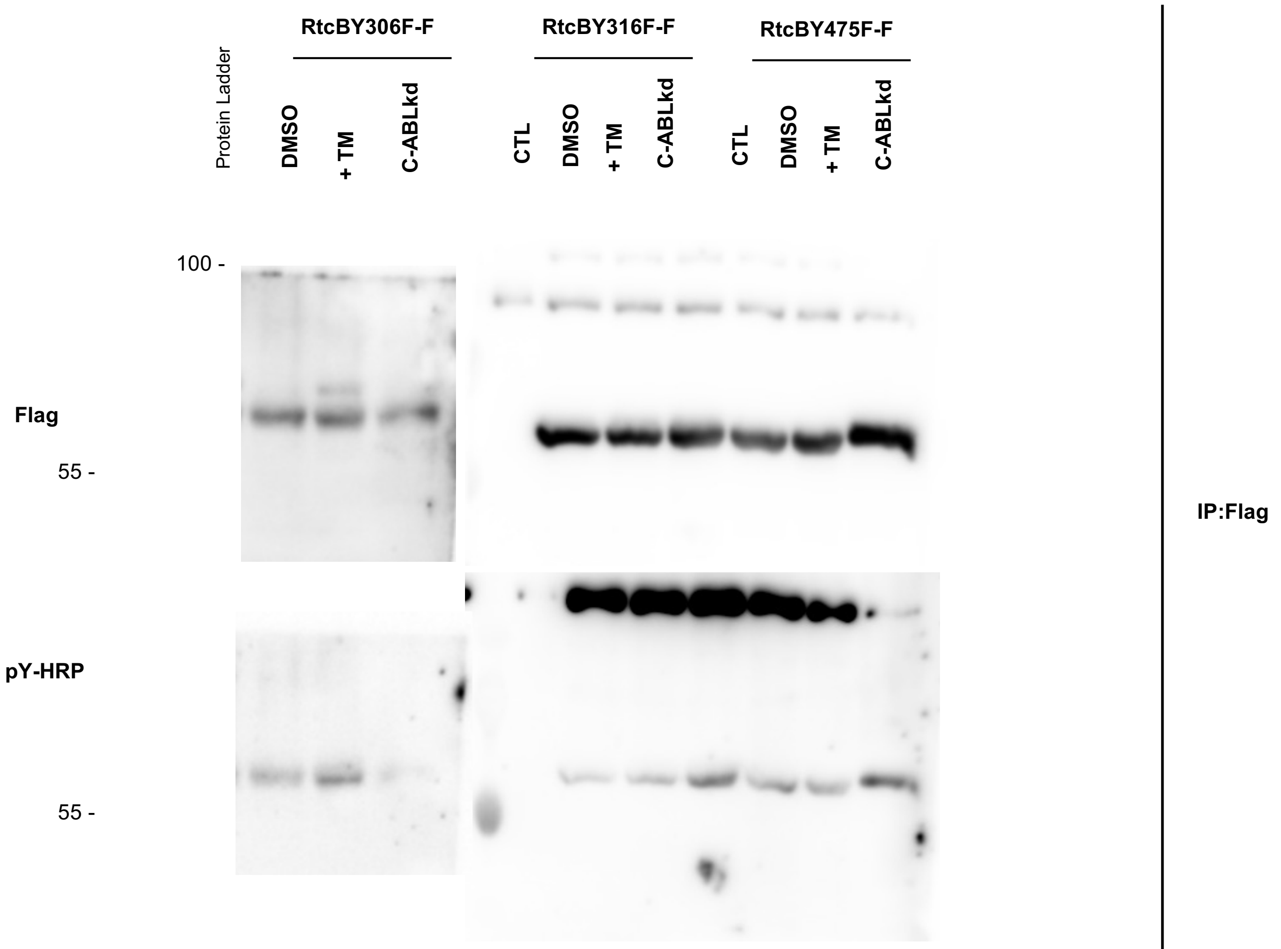

For the repeatitions A.2 and A.3 see Fig.2 F.2 and F.3 (pages 19-21)

B.Repeat n.1

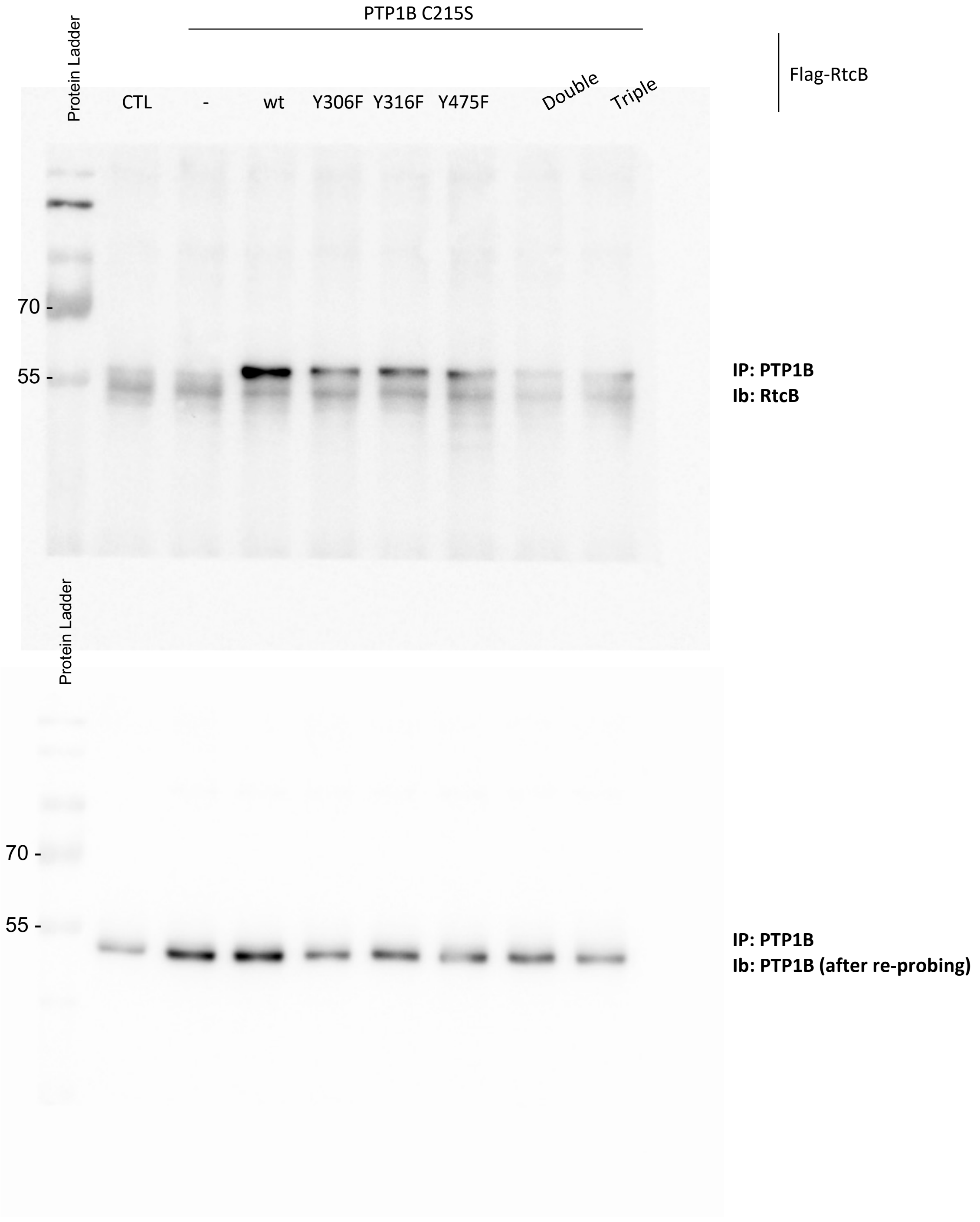
PTP1B C215S
Protein Ladder
CTL
-
wt
Y306F
Y316F
Y475F
Double
Triple
Flag-RtcB
70 -
55 -
IP: PTP1B
Ib: RtcB
Protein Ladder
70 -
55 -
IP: PTP1B
Ib: PTP1B (after re-probing)

Supplementary Figure 6.

B.Repeat n.1

In Fig.S6B

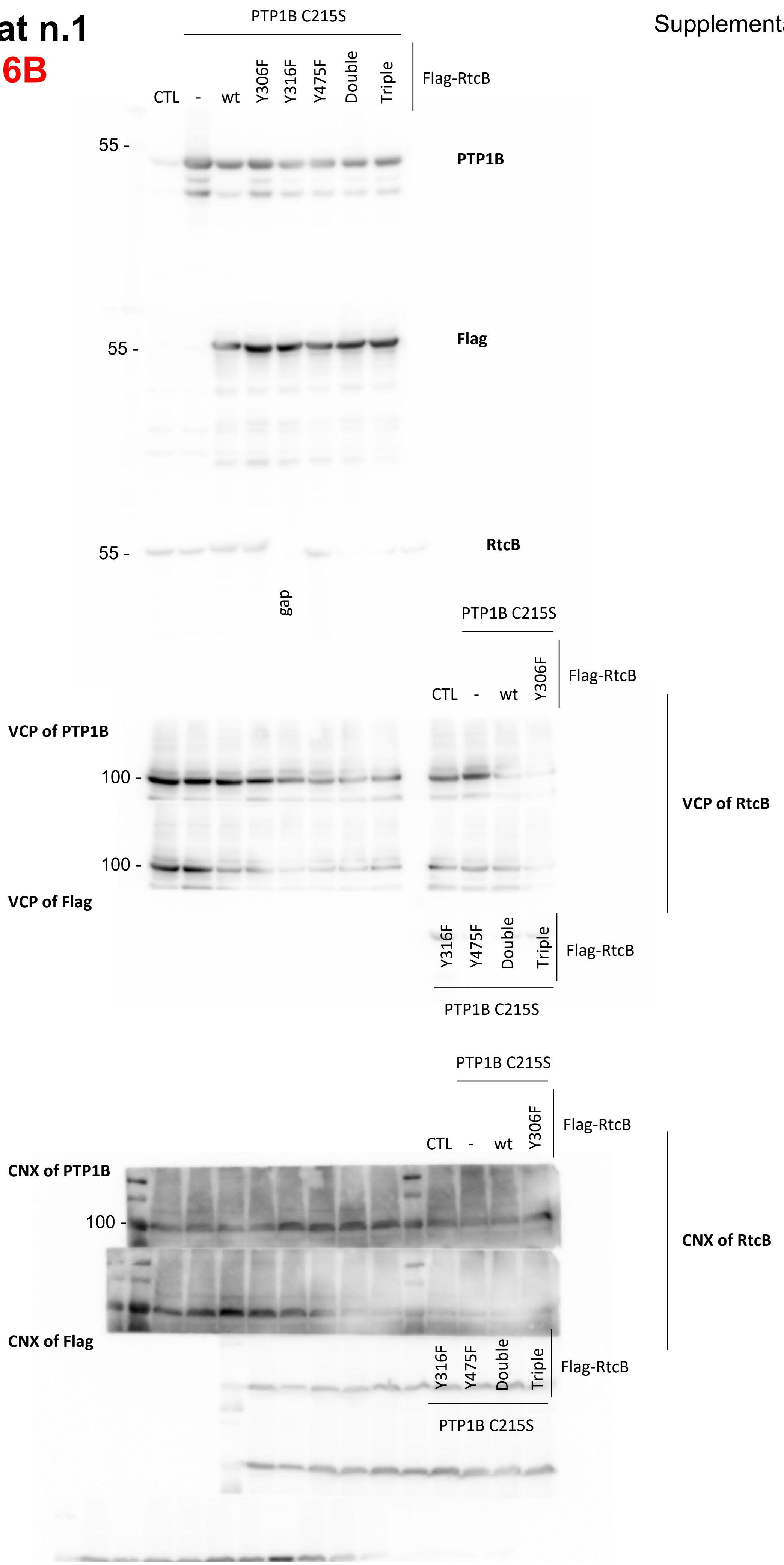

B.Repeat n.2

In Fig.S6B

Supplementary Figure 6.

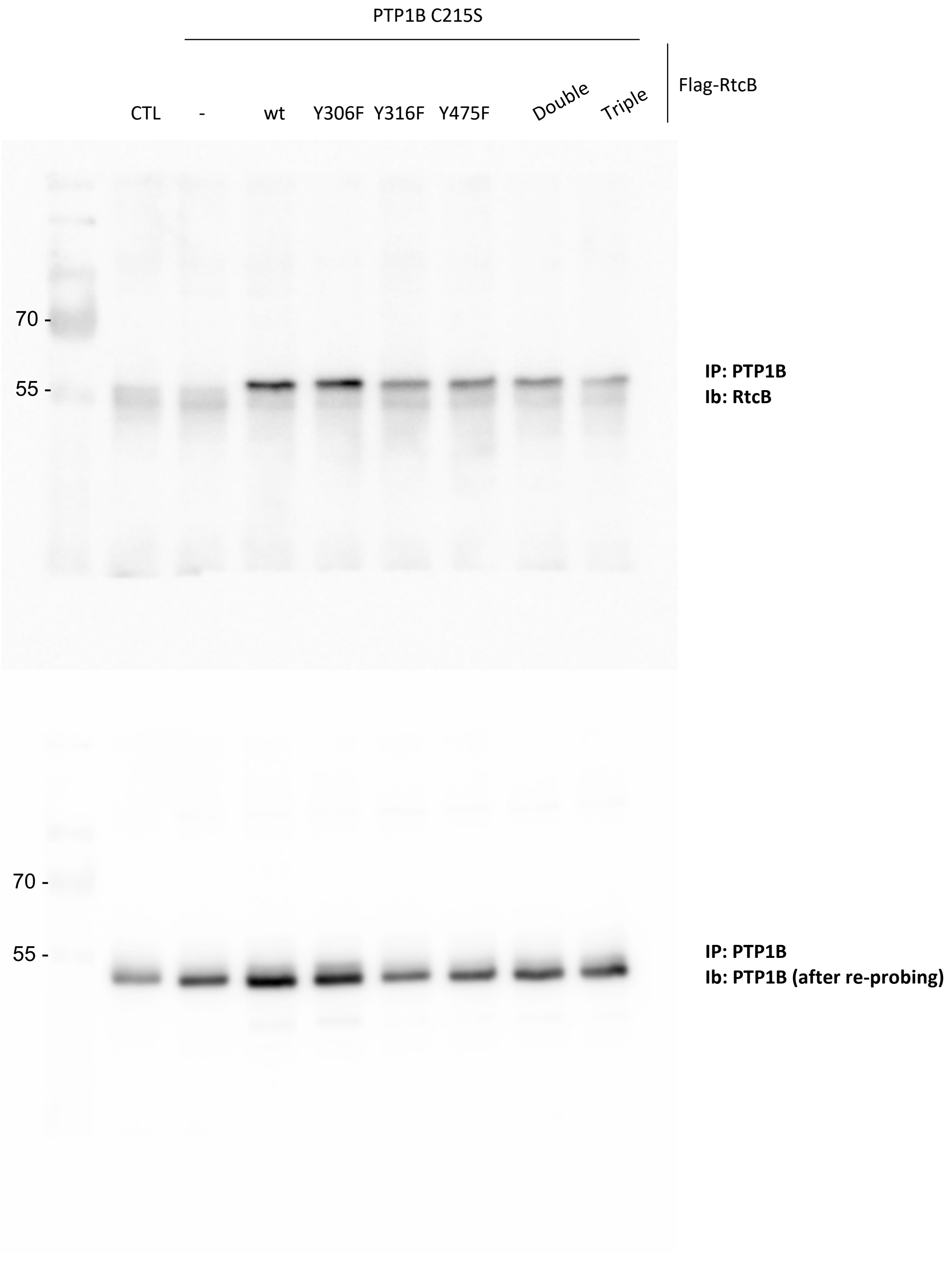

B.Repeat n.2

Supplementary Figure 6.

Inputs

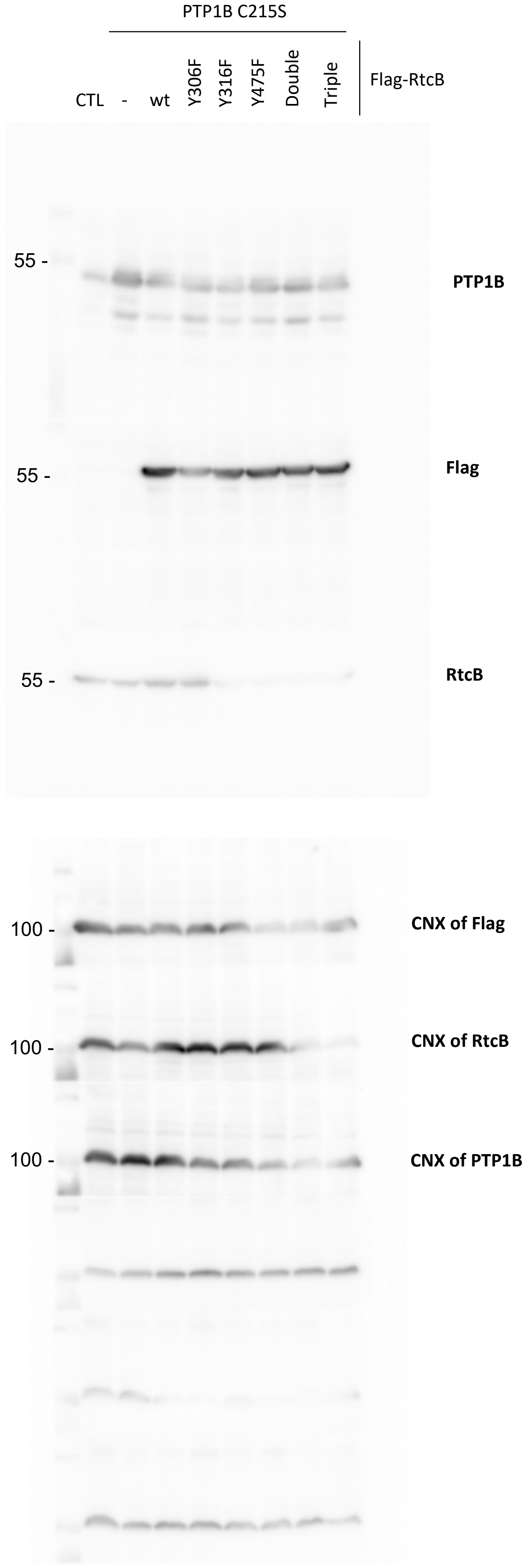

B.Repeat n.3

Supplementary Figure 6.

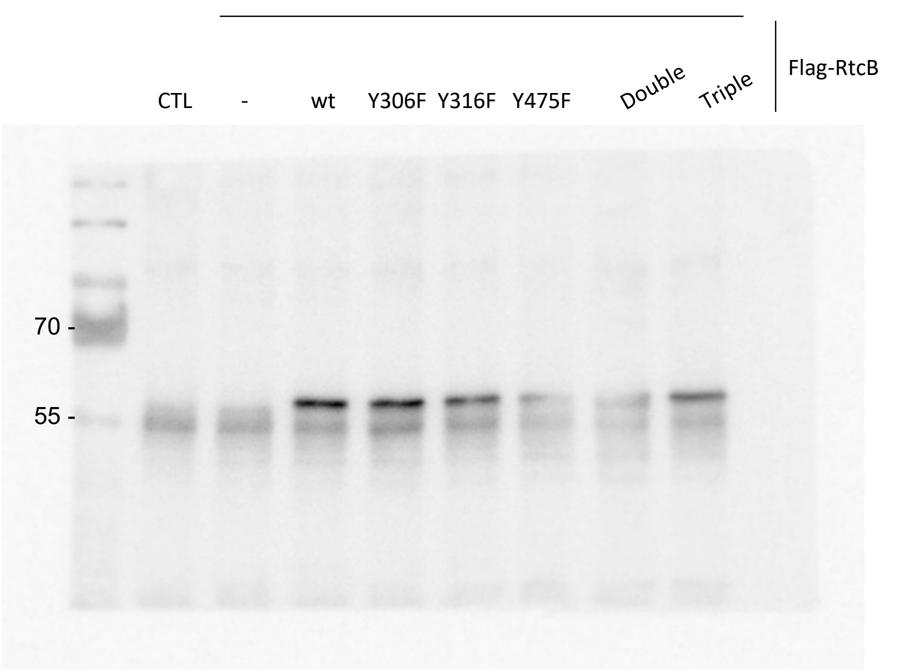

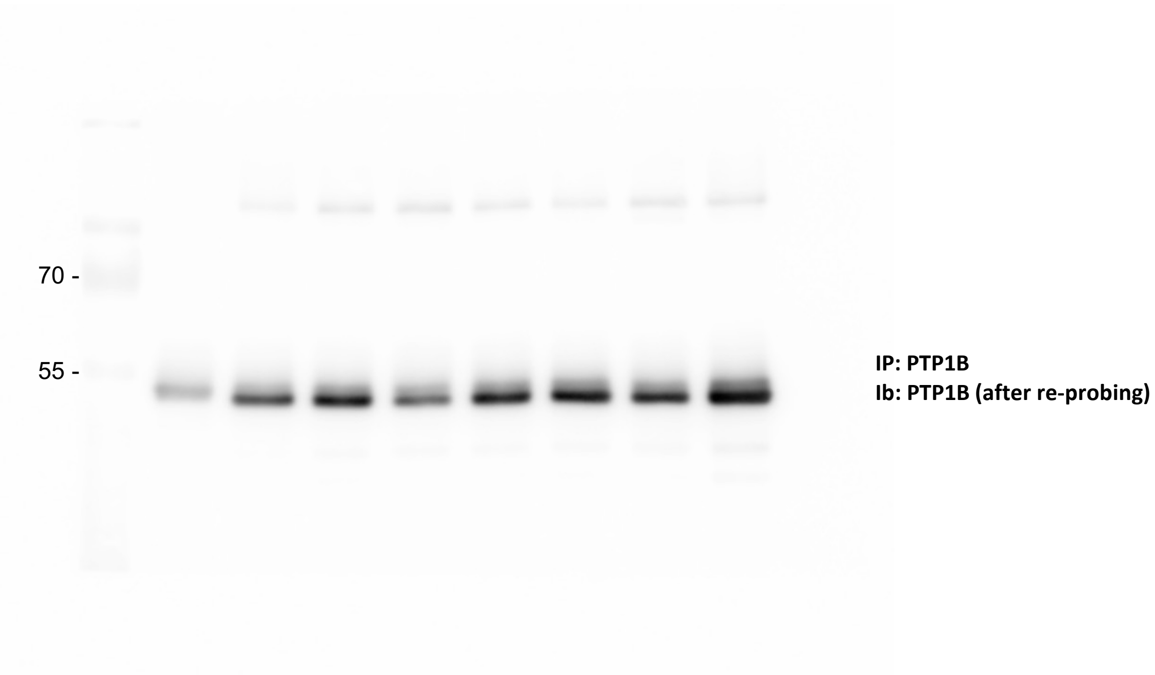

B.Repeat n.3

In Fig.S6B

Supplementary Figure 6.

Inputs

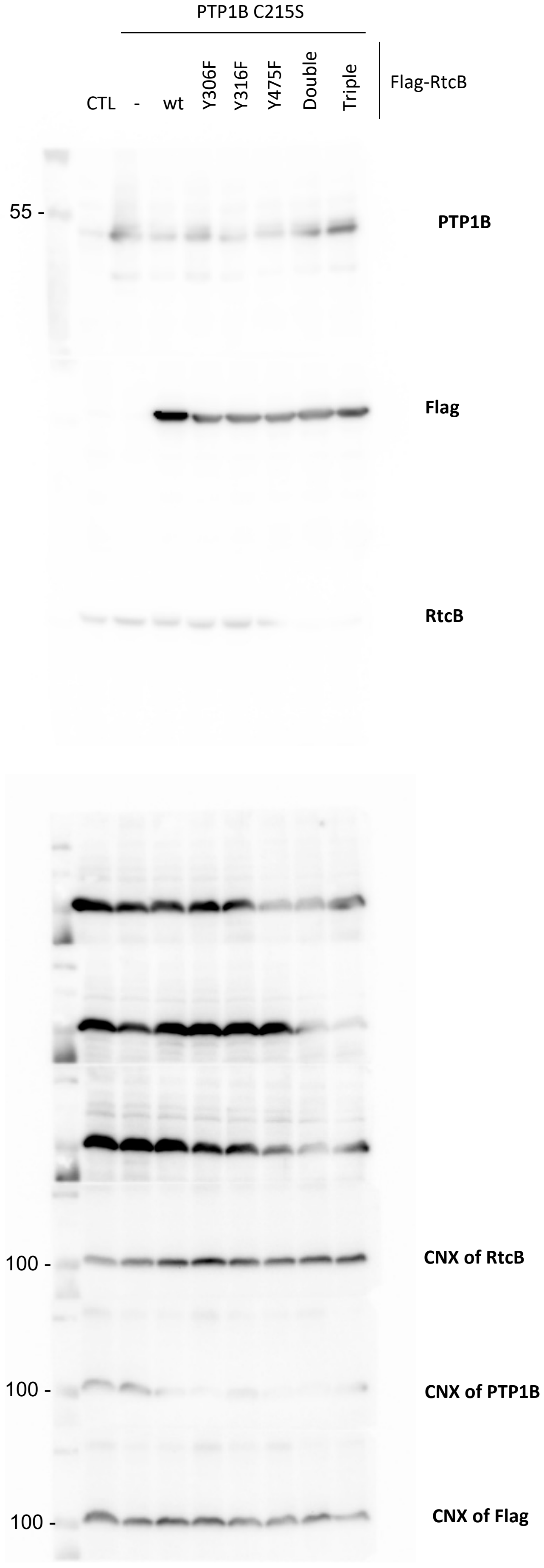

Supplement: Supplementary file 9 [file LSA-2022-01379_SdataFS6.zip › Source data FigS6/Source blots figS6.pdf]
