# Supplementary material for: Stress-induced tyrosine phosphorylation of RtcB modulates IRE1 activity and signaling outputs
Source: Life Sci Alliance. 2022 Feb 22;5(5):e202201379. doi: 10.26508/lsa.202201379 (PMC8899846; doi:10.26508/lsa.202201379)
Supplement: Supplementary file 15 [file LSA-2022-01379_TableS4.pdf]

Table S4: List of primers and new siRNA sequences used in this study.

**Site-directed mutagenesis primers for mouse RTCB**	**Sense (Forward 5'-3')**	**Anti-sense (Reverse 5'-3')**
Y306F - a917t	5'-ccattccctttagaaagtcttgtccctctgggga-3'	5'-tccccagagggacaagactttctaaagggaatgg-3'
Y316F - a947t	5'-acccaggcgaagtttccagctgcagcc-3'	5'-ggctgcagctggaaacttcgcctgggt-3'
Y475F - a1424t	5'-catggggacctgtagtttcgttctgactggca-3'	5'-catggggacctgtagtttcgttctgactggca-3'
H428A - c1282g-a1283c	5'-ggcccgctccagcacaggttgttccaaaggtctc-3'	5'-gagacctttggaacaacctgtgctggagcgggcc-3'
**qPCR primers** (*Homo sapiens*)		
PTPN1	5'-GAACCTTCTGTCTGGCTGATAC-3'	5'-CCCATCCGAAACTTCCTCATT-3'
XBP1s	5'-TGCTGAGTCCGCAGCAGGTG-3'	5'-GCTGGCAGGCTCTGGGGAAG-3'
XBP1 total	5'-CCTGGTTCTCAACTACAAGGC-3'	5'-AGTAGCAGCTCAGACTGCCA-3'
GAPDH	5'-AAGGTGAAGGTCGGAGTCAA-3'	5'-CATGGGTGGAATCATATTGG-3'
ACTB	5'-AGAGCTACGAGCTGCCTGAC-3'	5'-AGCACTGTGTTGCGCTACAG-3'
Tyr-GTA spliced	5'-CCTTCGATAGCTCAGTTGGTAGAGCG-3'	5'-ATTCGAACCAGCGaCCTAAGGATCTACA-3'
Tyr-GTA pre-tRNA unspliced	5'-CCTTCGATAGCTCAGTTGGTAGAGCG-3'	5'-GAGCCGGATTCGAACCAGCGaCCTAAGG-3'
Arg-TCT spliced	5'-GGCTCTGTGGCGCAATGGAtAGC-3'	5'-ACTCGAACCCGCAaCCTTTGAATTAGAA-3'
Arg-TCT pre-tRNA unspliced	5'-GGCTCTGTGGCGCAATGGAtAGC-3'	5'-TGGAGGGACTCGAACCCGCAaCCTTTGA-3'
Ile-TAT spliced	5'-GCTCCAGTGGCGCAATCGGTtAGC-3'	5'-GATCGAACTCACAaCCTCGGCATTATAA-3'
Ile-TAT pre-tRNA unspliced	5'-GCTCCAGTGGCGCAATCGGTtAGC-3'	5'-AGGTGAGGATCGAACTCACAaCCTCGGC-3'
Val-CAC	5'-GTTTCCGTAGTGTAGTGGTtATCACG-3'	5'-TTCGAACCGGGGACCTTTCGCGTGTG-3'
Pro-A/C/TGG	5'-GGCTCGTTGGTCTAGGGGTATGATTC-3'	5'-GGATTTGAACCCGGGACCTCTCGCAC-3'
U6 snRNA	5'-GCTTCGGCAGCACATATACTAAAAT-3'	5'-CGCTTCACGAATTTGCGTGTCAT-3'
3'-UTR-RTCB	5'-CACCTTTGGAAAGGGAGGAATA-3'	5'-AAGATCAGTATGACTGCGTAAGG-3'
**qPCR primers** (*Mus musculus*)		
BiP	5'-TTCAGCCAATTATCAGCAAACTCT-3'	5'-TTTTCTGATGTATCCTCTTCACCAGT-3'
CHOP	5'-TATCTCATCCCCAGGAAACG-3'	5'-CTGCTCCTTCTCCTTCATGC-3'
HERPUD	5'-ACCGCAGTTGGAGTGTGAGT-3'	5'-TGATCCAACAGCAGCTTCC-3'
XBP1s	5'-GAGTCCGCAGCAGGTG-3'	5'-GTGTCAGAGTCCATGGGA-3'
XBP1 total A	5'-GAATGGACACGCTGGATCCT-3'	5'-GCCACCAGCCTTACTCCACTC-3'
XBP1 total B	5'-GAATGGACACGCTGGATCCT-3'	5'-GTGTCAGAGTCCATGGGA-3'
18S	5'-GTAACCCGTTGAACCCCATT-3'	5'-CCATCCAATCGGTAGTAGCG-3'
**PCR primers** (*Mus musculus*)		
XBP1 total	5'-GAGAACCAGGAGTTAAGAACACG-3'	5'-GAAGATGTTCTGGGGAGGTGAC-3'
**siRNA sequences** (*Homo sapiens*)		
hs.Ri.RTCB.13.3 (IDT)	5'-AUCAGUAUGACUGCGUAAGGCAGUUUG-3'	5'-AACUGCCUUACGCAGUCAUACUGAT-3'
